# Does Trait Sexual Desire Predict Subjective Sexual Response to Erotic Stimuli? Effects of Participant Gender, Stimulus Gender, and Relationship Status Among Cisgender Heterosexual Women and Men

**DOI:** 10.1007/s10508-025-03298-w

**Published:** 2025-12-02

**Authors:** Milena Vásquez-Amézquita, Marina Begoña Martínez-González, Meredith L. Chivers, Juan David Leongómez

**Affiliations:** 1 Faculty of Psychology, Universidad El Bosque, Carrera. 9 # 131a- 02, 110121 Bogotá, Colombia; 2 Departamento de Ciencias Sociales, Universidad de la Costa, Barranquilla, Colombia; 3 Department of Psychology, Queen’s University, Kingston, ON Canada

**Keywords:** Trait sexual desire, Subjective sexual arousal, Gender-specific sexual response, Sexual motivation, Relationship status effects, Heterosexuality

## Abstract

Understanding how trait sexual desire (TSD) relates to subjective sexual arousal (SSA) is essential for clarifying how enduring sexual motivation influences momentary arousal responses, especially considering individual differences, gender-specific patterns, and relational contexts. This study examined how trait sexual desire relates to SSA across TSD dimensions, considering gender and relationship status. We tested 323 cisgender participants, 139 gynephilic men and 184 androphilic women, who anonymously assessed their TSD levels and rated their SSA while individually viewing erotic and non-erotic stimuli depicting both sexes. Results showed higher levels of TSD in men than in women. However, these differences were moderated by relationship status and were maintained only between men and women in a stable relationship. In addition, the association between TSD and SSA was gender-specific in men—stronger for sexual response to preferred sexual stimuli—and gender-nonspecific in women. Relationship status influenced TSD-SSA associations, especially in dyadic TSD toward a partner, highlighting the role of sociocontextual factors in the association between trait sexual desire and arousal among cisgender heterosexual individuals. The findings support the association between TSD and SSA, moderated by individual differences such as gender and sociocontextual factors like relationship status. We conclude that TSD affects SSA, and that TSD appears to be dynamic and context-dependent in nature.

**Supplementary Information:**

The online version contains supplementary material available at 10.1007/s10508-025-03298-w.

## Introduction

Understanding the relationship between trait sexual desire (TSD) and subjective sexual arousal (SSA) is crucial for elucidating how sexual motivation influences arousal responses to sexual cues, particularly in relation to individual differences, gender-specific patterns, and relational contexts. While both TSD and SSA reflect aspects of sexual motivation, they operate at different temporal and psychological levels. TSD represents a stable aspect of sexual motivation, whereas SSA reflects a momentary, subjective response to sexual stimuli (Chivers et al., 2010; Handy et al., 2020; Janssen et al., 2000). Investigating how these constructs are related is particularly relevant given the influence of gender and relationship contexts on sexual response patterns (Dawson & Chivers, 2014b; Peixoto et al., 2020; Timmers et al., 2018). Previous research has highlighted the complexity of these interactions, but limited studies have examined how multidimensional aspects of TSD predict SSA in varying relational contexts, especially considering gender differences. The current study focused on whether the relationship between TSD and SSA varies by participant gender, stimulus gender, and relationship status in heterosexual cisgender people.

### Defining Trait Sexual Desire and Subjective Sexual Arousal

Sexual response models, such as the incentive motivation model (IMM; Toates, 2009), suggest that sexual responses emerge from the processing of sexual cues—both internal (e.g., fantasies) and external (e.g., erotic stimuli)—which act as incentives motivating sexual behavior (Laan & Both, 2011). SSA refers to the subjective, emotional-cognitive experience of arousal in response to these cues (Janssen, 2011; Toledano & Pfaus, 2006). In contrast, TSD is conceptualized as a relatively stable cognitive-motivational disposition to engage in sexual activity (Stark et al., 2015). TSD can be measured across three dimensions: solitary TSD, dyadic TSD toward an attractive person, and dyadic TSD toward a partner (Moyano et al., 2017; Sierra et al., 2020). According to trait activation theory (TAT; Tett et al., 2021), TSD is a latent trait that becomes activated in response to relevant situational cues, such as erotic stimuli, which then elicit SSA. However, like other personality traits, the expression of TSD can be influenced by situational factors without compromising its underlying stability (Fleeson, 2001). While individuals with higher TSD are generally more responsive to sexual cues (Peixoto et al., 2020; Sierra et al., 2020), this relationship may be influenced by individual and contextual factors (Baumeister, 2000; Harris et al., 2023; Rosenkrantz & Mark, 2018).

### Gender Influences on Trait Sexual Desire

Gender differences in sexual response patterns are well-documented. Men typically report higher TSD than women across most dimensions (Carvalho & Nobre, 2010; Peixoto et al., 2020), although some studies have found no gender differences (Goldey & van Anders, 2012). In terms of SSA, men tend to exhibit greater gender-specific arousal, responding more intensely to stimuli that align with their sexual preferences, while heterosexual women demonstrate greater flexibility, often showing arousal that is less dependent on the gender of the stimulus (Chivers, 2017; Chivers et al., 2010; Dawson & Chivers, 2014b; Timmers & Chivers, 2018; Vásquez-Amézquita et al., 2019).

Recent evidence suggests that, while short-term fluctuations in sexual desire are similar for men and women, women’s TSD shows greater variability over longer time periods (Harris et al., 2023). These differences may be influenced by relational and emotional factors, with women’s TSD being more sensitive to relational contexts compared to men’s more cue-driven desire (Baumeister, 2000; Rosenkrantz & Mark, 2018).

Sierra et al. (2020) examined the relationship between SSA and TSD and found that only TSD toward an attractive person predicted SSA in men. In contrast, none of the other TSD dimensions—including solitary and partner-directed TSD—predicted SSA in either men or women. These findings suggest potential gender differences in how TSD dimensions predict components of sexual arousal.

### Effect of Relationship Status on Trait Sexual Desire

Relationship status significantly influences TSD and its association with SSA (Dawson & Chivers, 2014b; Peixoto et al., 2020; Timmers et al., 2018). Individuals in stable relationships often report lower solitary TSD but higher dyadic TSD toward their partner compared to single individuals (Moyano et al., 2017; Sierra et al., 2020). Relationship dynamics such as intimacy, novelty, and partner responsiveness further modulate these patterns (McNulty et al., 2019). Studies have also shown that masturbation frequency—closely linked to solitary TSD—is moderated by relationship status (Huang et al., 2022; van Anders, 2012).

Notably, research examining how TSD dimensions predict SSA has yielded mixed findings. For example, Sierra et al. (2020) found that, in individuals currently in a relationship, dyadic TSD toward an attractive person predicted SSA in men but not in women. Carvalho and Nobre (2010), however, found gender effects in TSD regardless of relationship status. Other studies have highlighted that commitment levels (Rodrigues & Lopes, 2017) and relationship satisfaction (Blumenstock et al., 2024; Holmberg & Blair, 2009; Peixoto, 2019) also influence how TSD translates into SSA, underscoring the complexity of relational contexts impacts on sexual response.

The moderating role of relationship status on the association between TSD and SSA remains underexplored. Taken together, the limited evidence highlights the importance of relationship status in shaping the association between TSD and SSA, as it might influence how TSD manifests in different relational contexts.

### The Present Study

The present study aims to empirically test the association between TSD and SSA while examining how gender, stimulus gender, and relationship status moderate this association in cisgender, heterosexual individuals. Building on the trifactorial model of TSD (Moyano et al., 2017; Sierra et al., 2020), we examined how solitary TSD, dyadic TSD toward an attractive person, and dyadic TSD toward a partner predicted SSA in response to erotic stimuli depicting either women or men, among heterosexual cisgender individuals.

Recognizing the inconsistency in prior findings, this study addressed these gaps by considering multidimensional TSD components, gender effects, and relational contexts simultaneously.

We tested two directional hypotheses and one exploratory non-directional hypothesis, considering the potential influence of participant gender, stimulus gender, and relationship status:

#### Hypothesis 1

: Men were expected to report higher TSD than women across all dimensions (**H1a:** Solitary TSD; **H1b:** Dyadic TSD toward an attractive person; **H1c:** Dyadic TSD toward a partner); Carvalho & Nobre, 2010; Peixoto et al., 2020), with relationship status moderating these effects (Moyano et al., 2017; Sierra et al., 2020).

#### Hypothesis 2

: Associations between TSD and SSA were expected to differ by TSD dimension (Sierra et al., 2020), with gender-specific patterns in men and gender-nonspecific patterns in women (Chivers, 2017; Dawson & Chivers, 2014b).

**H2a:** No significant association was expected between solitary TSD and SSA.

**H2b:** A significant association was expected between dyadic TSD toward an attractive person and SSA.

**H2c**: No significant association was expected between dyadic TSD toward a partner and SSA.

#### Hypothesis 3

(Exploratory): Participant Gender and relationship status would moderate the associations between TSD dimensions (**H3a:** Solitary TSD; **H3b:** Dyadic TSD toward an attractive person; **H3c:** Dyadic TSD toward a partner) and SSA, with potential three-way interactions.

To test these hypotheses, we analyzed data from cisgender heterosexual individuals using nine independent models.

## Method

### Participants

Participants were recruited in Colombian through social networks, email, and university student databases. The advertisement read: "People between 18 and 40 years old, men and women, are invited to participate in a completely anonymous online survey on the association between arousal and sexual desire". A total of 681 people opened the link. Participation for people who reported never having been exposed to sexually explicit material or pornography, who reported no sexual activity with a partner during the past 6 months, or women who were pregnant, postpartum or nursing during the first three months, was terminated and participants were thanked for their interest. Among those who were able to advance in the survey, only men with exclusively or principally gynephilic sexual attractions (i.e., sexual attraction to women) (*n* = 237) and exclusively androphilic women (i.e., sexual attraction to men) (*n* = 277), according to the Kinsey scale (Kinsey et al., 2003), were selected for this study in order to control for the effect of variations in gender specificity of sexual responses in women with any degree of gynephilia (Chivers, 2017; Chivers et al., 2015; Dawson et al., 2017; Luoto & Rantala, 2017). Asexual individuals were explicitly excluded by including a specific category for asexuality on the Kinsey scale, ensuring the sample comprised individuals expressing sexual desire for others, consistent with the aims of the study (Table S1; Figure S2 in supplementary material).

In addition, participants who did not complete the sexual desire scales or evaluated less than 80% of the sexual stimuli (n = 164; 72 men and 92 women) were excluded from analyses; and finally, from this selection we excluded those participants who agreed at the end of the survey to have taken the survey without reading the questions or for joking around (n = 17). Finally, given our interest in the effect of relationship status, we excluded the 10 participants (five men and five women) who reported a non-stable relationship (casual or non-committed relationship, e.g., dating without exclusivity or long-term commitment) as they represented a very small sample to avoid biases in the analyses.

A total of 323 participants, 139 men, single = 67; stable relationship exclusive partnership lasting more than 6 months) = 72, with or without cohabitation and 184 women (single = 79; stable relationship = 105), between 18 and 40 years of age were included in the final sample of this study (for more details about sample see Supplementary Tables S1, S2; Figs. S1, S2).

### Measures

*Sexual Desire Inventory* (SDI; Spector et al., 1996). The Spanish-adapted, three-dimensional version (Moyano et al., 2017) was used to measure TSD. It is composed of 13 items measuring Dyadic sexual desire toward a partner (Items 1, 2, 3, 7, 8), dyadic sexual desire toward an attractive person (Items 4, 5, 6, 9), and solitary sexual desire (Items 10, 11, 12, 13). Ten of the items (3–9, and 11) measured the strength and intensity of desire on a continuous scale of 0 to 8, and the remaining three items (1, 2, and 10) were rated on a scale of 0 to 7, where frequency (of desire or thoughts) was assessed: 0 = Never to 7 = Many times a day. The score is obtained from the sum of the items in each dimension. The higher the score, the greater the sexual desire. The maximum scores for each scale are different: Solitary sexual desire = 31; Dyadic sexual desire toward partner = 38; and Dyadic sexual desire toward attractive person = 32.

The internal consistency in the Colombian population has been reported between .80 and .88 for men and women respectively in the sexual desire towards a partner dimension, between .86 and .89 for the sexual desire towards an attractive partner dimension, and between .90 and .96 for the Solitary sexual desire dimension (Moyano et al., 2017). In recent studies in which the relationship between arousal and sexual desire has been measured, the internal consistency was .53 for Sexual desire towards a partner, .73 for Sexual desire towards an attractive person and .85 for Sexual solitary desire (e.g. Sierra et al., 2020). Our internal consistency analyses showed a consistency of .92 for the Dyadic sexual desire (Partner) scale, .93 for the Solitary sexual desire scale, and.91 for the Dyadic sexual desire (Attractive person) scale (Supplementary Table S3).

*Stimuli* Stimuli consisted of 30 erotic and 30 non-erotic stimuli of each sex (male and female) presented individually on a screen. The erotic sexual stimuli were drawn from a stimulus set previously used in experimental studies on sexuality (Dawson & Chivers, 2016). These were pictures of nude males and females in sexually suggestive positions with visibly aroused genitalia (erect penis and exposed vulva). The non-erotic sexual stimuli were downloaded from free internet sites and adjusted in physical characteristics and positions like those of the erotic stimuli. These pictures were of clothed people, standing and mostly with a smiling facial expression. All images were presented without background and were adjusted for size, color, lightness, and brightness using Photoshop. Both erotic and non-erotic pictures were adjusted to a size between 300 × 400 pixels and presented in grayscale. Grayscale images were used to minimize variability in colour perception due to differences in participants' personal screen settings. This ensured uniform brightness and contrast across stimuli, enhancing experimental control and data consistency. Each participant viewed four blocks of stimuli (female-erotic; female-non-erotic; male-erotic; male-non-erotic), with both the order of both the blocks as well as the images within each block fully randomized. Each stimulus was presented in the center of the screen and the participant had the option to evaluate the requested dimensions at his or her own pace. Sexual arousal and sexual attractiveness questions appeared below the presented image.

*Subjective Sexual Arousal (SSA)* Subjective sexual arousal was assessed via self-reported subjective sensations of genital and non-genital sexual arousal (Gómez-Lugo et al., 2016; Vallejo-Medina, 2017) in which participants were asked to rate, "how much genital (penile erection or vaginal lubrication) or non-genital (e.g., warmth, tingling, absorbed or focused on the stimulus, floating sensation) sensations did you experience when you saw the image?" on a scale from 0 (No arousal) to 7 (Extreme arousal).

### Procedure

The study began with the presentation of the research aims, reading and signing of the informed consent form, and confirmation of inclusion criteria for participation. Participants completed a questionnaire of sociodemographic data and sexual and partner history, followed by measures of sexual satisfaction and functioning (Supplementary Table S2), and all dimensions of the Sexual Desire Inventory (SDI; Moyano et al., 2017; Spector et al., 1996), that was the main outcome measure for this study. All scales, including the SDI, were presented to participants prior to the presentation of any sexual stimuli to prevent the effect of stimulus exposure on self-report of trait sexual desire (Goldey & van Anders, 2012).

After viewing an image, all participants individually rated their SSA to erotic and non-erotic sexual stimuli of both genders (women and men), the attractiveness of the image, and classified the image according to age and evoked emotion (disgust, fear, anger, shame, pleasure, satisfaction, none). For the analyses here reported, data collected for attractiveness, age and emotion were not considered.

### Statistical Analyses

All data analysis was performed using R version 4.2.2 (R Core Team, 2024) and coding of all statistical analyses, figures, and tables was created as an R Markdown file. To test hypothesis 1, we fit LMs and for Hypotheses 2 and 3, we fit Linear Mixed-Effects Models (LMM) using the *lmerTest* (Kuznetsova et al., 2017) package.

The results were organized in regression-type tables reporting main effects and interactions, but *Sum-to-zero* contrasts were used to display *p*-values that represent main effects and interactions in an ANOVA-type manner (i.e., the intercept is the grand mean of all cells, and estimates are differences between each category mean and the mean of all categories). Since both fixed and random effects were analyzed, it is not possible to obtain effect sizes for each term, so we report Nakagawa's R^2^ values (both marginal and conditional; Nakagawa & Schielzeth, 2013), which correspond to overall effect sizes for the whole model (excluding and including random effects, respectively). For all models, assumptions and probability distributions were explored using the functions *check_model* and *check_distribution* from the package *performance* (Lüdecke et al., 2021). These results are included for each model in the Supplementary Material.

To control for the effect of partner sexual satisfaction and relationship duration in individuals with a partner, we analyzed the influence of these variables on each TSD dimension. While relationship duration had no significant effect on any TSD dimension, sexual satisfaction was found to affect dyadic TSD toward a partner. To account for these effects, we adjusted dyadic TSD toward a partner by setting its associations with both sexual satisfaction and relationship duration to zero. The transformed scores were then used in all subsequent models.

For Hypothesis 1, we explored the differences in each dimension of TSD between men and women according to relationship status, we performed a linear model (LM) for each dimension of TSD using participant gender and relationship status as predictors and analyzed both main effects and their interactions. However, in all cases models did not meet the requirement of normality of residuals, so the dependent variable was transformed to a normal distribution using Ordered Quantile (ORQ) normalization transformations (Peterson & Cavanaugh, 2020) through the function *orderNorm* from the package *bestNormalize* (Peterson, 2021).

For all models in Hypotheses 2 and 3, the outcome variable was Subjective sexual arousal, which was measured in a 7-point scale. Given the discrete and ordinal nature of this scale, we fitted each model as a Cumulative Link Mixed Model (CLMM, using the *clmm* function from the package ordinal; Christensen, 2023) to account for the ordinal nature of the scale, as a Generalized Linear Mixed Model (GLMM, using the *glmer* function from the lme4 package; Bates et al., 2015) with a Poisson distribution to account for the discrete nature of the data, and as a LMM, given its advantages in simplicity and ability to get information from the model, including performing simple slope analyses. In all cases, the pattern of results was quite robust, so inferences were drawn and are reported from the LMMs. GLMMs and CLMMs are reported and compared in the Supplementary Materials.

For Hypotheses 2 and 3, we avoided complex interactions that would increase the error rate and underpower the study, answering the main hypotheses. First, we performed a GLMM to predict SSA that included participant gender, stimulus gender, and stimuli content as fixed factors. We found that as expected, erotic stimuli produced an SSA in the same direction, but significantly greater than non-erotic stimuli. Given this, we included only erotic stimuli in the analysis (Table S17; Figure S9).

To address Hypothesis 2 about gender-specificity in the association between TSD and SSA toward erotic stimuli, we performed mixed models for each TSD dimension. The models included participant gender and stimulus gender as fixed effects, with TSD dimension included as a covariate. Random intercepts effects included participant and stimulus code to make results generalizable to a population of both participants and stimuli. We analyzed the main effects and their interactions.

Finally, for Hypothesis 3, on exploring whether the predictive association between each dimension of TSD and SSA toward erotic stimuli of the preferred gender would be modulated by relationship status, we performed a GLMM with each dimension of TSD as a covariate, including only self-reported stimuli as preferred, and taking participant gender and relationship status as fixed factors, and keeping stimulus and participant as random intercepts.

Subsequent to each model, depending on the significant interactions of interest and whether an interaction included a covariate, we performed different *post-hoc* analyses: for interactions including a covariate we used simple slope analysis using the package *interactions* (Long, 2019), whereas for interactions between factors we used contrasts of estimated marginal means with Bonferroni correction, using the package *emmeans* (Lenth, 2022). It is important to note that these should be considered as unstandardized effect sizes. We have not reported standardized effect sizes because there is no universally agreed-upon method for calculating standard effect sizes for individual model terms, including main effects or interactions, given the way variance is partitioned in linear mixed models (Rights & Sterba, 2019).

All figures were created using *ggplot2* (Wickham, 2009), and all tables were created with *knitr* (Xie, 2018) and *kableExtra* (Zhu, 2021).

## Results

All data and code used to perform these analyses are openly available from the Open Science Framework (OSF) project (10.17605/OSF.IO/3V2E7). The code for all analyses, tables and figures is available not only as PDF ('Supplementary_Material.pdf'), but also in R Markdown ('Supplementary_Material.Rmd') format, so that it can be fully reproduced.

All descriptive statistics (Supplementary Table S1; Figs. S1, S2) and correlations (Supplementary Table S2) between all variables such as age, sexual functioning, TSD, SSA toward each type of stimulus (erotic vs. non-erotic; male vs. female) according to participant gender, are reported in the Supplementary Material.

### Participant Gender and Relationship Status Effects on Trait Sexual Desire (H1)

**Fig. 1:**
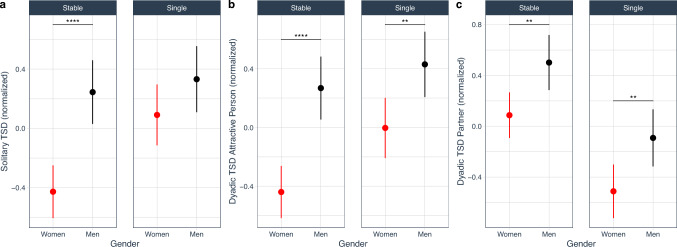
Effects of gender and relationship type on dimensions of Trait Sexual Desire (TSD). All dimensions of TSD were transformed using ordered quantile normalization (Peterson & Cavanaugh, 2020). **a** Solitary Trait Sexual Desire (for details, see Tables S6, S7, S8); **b** Dyadic Trait Sexual Desire—Attractive Person (for details, see Tables S10, S11, S12); **c** Dyadic Trait Sexual Desire—Partner (for details, see Tables S14, S15, S16). Dots and bars represent estimated marginal means and 95% CI. In all cases, significant effects are represented with lines and stars: *p < .05, **p < .01, ***p < .001, ****p < .0001

We found main effects of gender and relationship status on all dimensions of the TSD (Table 1, Fig. 1). Overall, men had reported higher scores than women on all three TSD dimensions (H1a, Solitary TSD: Mean_difference = − 0.46, SD = 0.1, *t*[319] =  − 4.36, *p* < .0001, Fig. 1a; H1b, Dyadic TSD towards attractive person: Mean_difference = − 0.57, SD = 0.1, *t*[319] =  − 5.46, *p* < .0001, Fig. 1b; H1c, Dyadic TSD towards partner: Mean_difference = − 0.42, SD = 0.11, *t*[316] =  − 3.94, *p* < .001, Fig. 1c).

**Table 1:** Effects of relationship type and gender on trait sexual desire dimensions

Effect	Trait sexual desire dimensions											
	Solitary trait sexual desire				Dyadic trait sexual desire attractive person				Dyadic trait sexual desire partner			
	df	*F*	*p*	*ϵ*^2^_*p*_	df	*F*	*p*	*ϵ*^2^_*p*_	df	*F*	*p*	*ϵ*^2^_*p*_
Gender	1319	22.42	**< .0001**	0.06	1319	29.85	**< .0001**	0.09	1316	15.49	**< .0001**	0.04
Relationship	1319	14.07	**< .0001**	0.03	1319	8.2	**.0004**	0.03	1316	31.6	**< .0001**	0.09
Gender × Relationship	1319	4.23	**.04**	0.01	1319	1.73	.19	0.00	1316	0.00	.98	< 0.001

Sexual desire was transformed using an ordered quantile normalization (Peterson & Cavanaugh, 2020). Results are type III ANOVA. Solitary Trait Sexual Desire: *R*^*2*^ = 0.103, *R*^*2*^_*adjusted*_ = 0.095; Dyadic Trait Sexual Desire—Attractive Person: *R*^*2*^ = 0.122, *R*^*2*^_*adjusted*_ = 0.114; Dyadic Trait Sexual Desire Partner: *R*^2^ = 0.125, *R*^2^_adjusted_ = 0.117. Gender = participants gender (women, men); Relationship = relationship type (stable, single). As effect size, we report partial epsilon squared (ϵ^2^_*p*_), which provides a less biases estimate than *η*^2^ (see Albers & Lakens, 2018). Significant effects are in bold

We found only one interaction between participant gender and relationship status for solitary TSD (Table 1). Among people in a relationship, men reported higher solitary TSD than women did (Mean_difference = − 0.67, SD = 0.14, *t*[319] =  − 4.74, *p* < .0001); among single people, there was no difference between men and women in solitary TSD (Mean_difference = − 0.24, SD = 0.15, *t*[319] = -1.57, *p* = 0.12). No significant interactions were found between gender and relationship status on any of the dimensions of dyadic TSD (towards attractive person or partner; Table 1; Fig. 1a).

### Trait Sexual Desire and Subjective Sexual Arousal: Gender and Stimulus Gender Effect (H2)

**Fig. 2:**
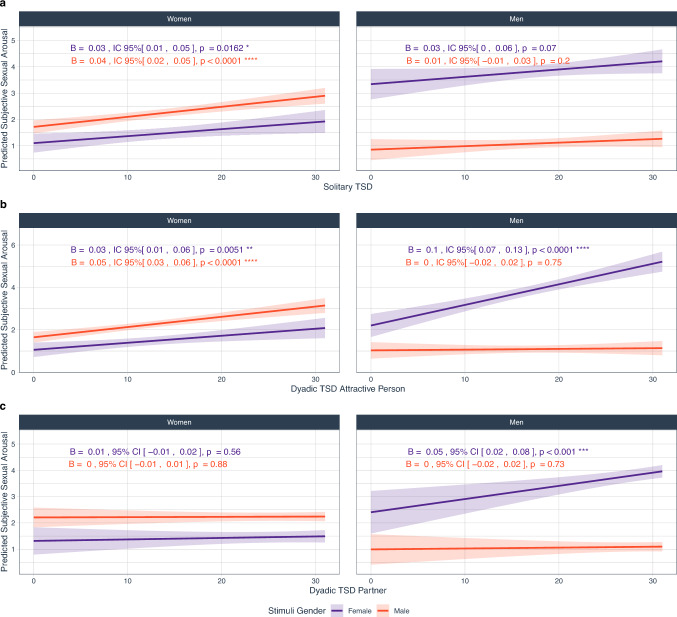
Slopes of trait sexual desire dimensions on sexual subjective arousal, by gender and stimulus gender. **a** Solitary trait sexual desire (for details, see TableS21); **b** Dyadic trait sexual desire—attractive person (for details, see Table S24); **c** Dyadic trait sexual desire—partner (for details, see Table S27). Lines represent simple slopes and 95% CI. Significant effects are represented with stars alongside slope details: *p < 0.05, **p < .01, ***p < .001, ****p < .0001

We found a main effect of each TSD dimension on SSA. Furthermore, significant two-way interactions emerged between TSD dimension and participant gender, as well as between TSD dimension and stimulus gender, but only for the dyadic TSD dimensions. Finally, three-way interactions involving TSD dimension, gender, and stimulus gender were also observed in the dyadic dimensions (toward an attractive person and toward a partner), but not in the solitary TSD dimension (Table 2, Fig. 2).

**Table 2:** Effects of trait sexual desire dimensions on subjective sexual arousal across gender and stimulus gender

	Effect	LMM			*ϵ*^2^_*p*_
		*df*	*F*	*p*	
Solitary TSD	Solitary trait sexual desire	1319	17.46	**< .0001**	0.0489
	Gender	1319	8.84	**.0032**	0.0239
	Stimulus gender	1369	24.71	**< .0001**	0.06
	Solitary sexual desire × Gender	1319	0.85	0.36	0.0001
	Solitary sexual desire × Stimulus gender	1319	0.02	0.88	0.0001
	Gender × Stimulus gender	1319	74.79	**< .0001**	0.19
	Solitary sexual desire × Gender × Stimulus gender	1319	1.78	0.18	0.0024
Dyadic TSD attractive person	Dyadic TSD—Attractive Person (AP)	1319	48.49	**< .0001**	0.13
	Gender	1319	1.446	0.23	0.0014
	Stimulus gender	1374	2.689	0.101	0.0045
	Dyadic TSD Attractive person × Gender	1319	0.53	0.467	0.001
	Dyadic TSD Attractive person × Stimulus gender	1319	15.428	**< .0001**	0.0431
	Gender × Stimulus gender	1319	27.445	**< .0001**	0.08
	Dyadic TSD Attractive person × Gender × Stimulus gender	1319	29.689	**< .0001**	0.08
Dyadic TSD partner	Dyadic TSD—Partner (P)	1316	6.589	**0.01**	0.0173
	Gender	1316	0.034	0.853	< 0.0001
	Stimulus gender	1344	0.991	0.32	< 0.0001
	Dyadic TSD Partner × Gender	1316	3.97	**0.047**	0.0093
	Dyadic TSD Partner × Stimulus gender	1316	4.854	**0.003**	0.0203
	Gender × Stimulus gender	1316	20.55	**< .0001**	0.06
	Dyadic TSD Partner × Gender × Stimulus gender	1316	5.7	**0.018**	0.0146

Results are type III ANOVA. Solitary Trait Sexual Desire: *R*^*2*^_*conditional*_ = 0.745, *R*^*2*^_*marginal*_ = 0.335; Dyadic Trait Sexual Desire—Attractive Person: *R*^*2*^_*conditional*_ = 0.745, *R*^*2*^_*marginal*_ = 0.367; Dyadic Trait Sexual Desire—Partner: *R*^*2*^_*conditional*_ = 0.745, *R*^*2*^_*marginal*_ = 0.329. Gender = participant gender (women, men); Stimulus gender = gender of stimuli (male, female). Partial epsilon squared (ϵ^2^_*p*_) is used as effect size, as it provides a less biased estimate than *η*^2^ (see Albers & Lakens, 2018). Significant effects are in bold

The association between solitary TSD and SSA (**H2a**) was significant in the case of women responding to both stimuli depicting men (*B* = 0.04, 95% CIs [0.02, 0.05], *p* < .001) and depicting women (*B* = 0.03, 95% CIs [0.01, 0.04], *p* < .001), but non-significant for men responding to stimuli depicting women (*B* = 0.03, 95% CIs [0.00, 0.06], *p* = .07), or men (*B* = 0.01, 95% CIs [− 0.01, 0.03], *p* = .20; Fig. 2a).

Similarly, the association between dyadic TSD toward an attractive person and SSA (**H2b**) was significant in the case of women responding to both stimuli depicting men (*B* = 0.05, 95% CIs [0.03, 0.06], *p* < .001) and women (*B* = 0.03, 95% CIs [0.01, 0.06], *p* = .005), and in the case of men responding to stimuli depicting women (*B* = 0.10, 95% CIs [0.07, 0.13], *p* < .001), but not to stimuli depicting men (*B* = 0.003, 95% CIs [− 0.02, 0.02], *p* = .75; Fig. 2b).

Finally, the association between dyadic TSD toward a partner and SSA (**H2c**) was not significant in the case of men responding to stimuli depicting men (*B* = 0.00, 95% CIs [− 0.02, 0.02], *p* = .73), nor women when presented with stimuli depicting men (*B* = 0.00, 95% CIs [− 0.01, 0.01], *p* = .88) or women (*B* = 0.010, 95% CIs [− 0.01, 0.02], *p* = .56) stimuli. There was, however, a significant slope for men responding to stimuli depicting women (*B* = 0.05, 95% CIs [0.02, 0.08], *p* < .001; Fig. 2c).

### Trait Sexual Desire and Subjective Sexual Arousal: Participant Gender and Relationship Status Effects (H3)

**Fig. 3:**
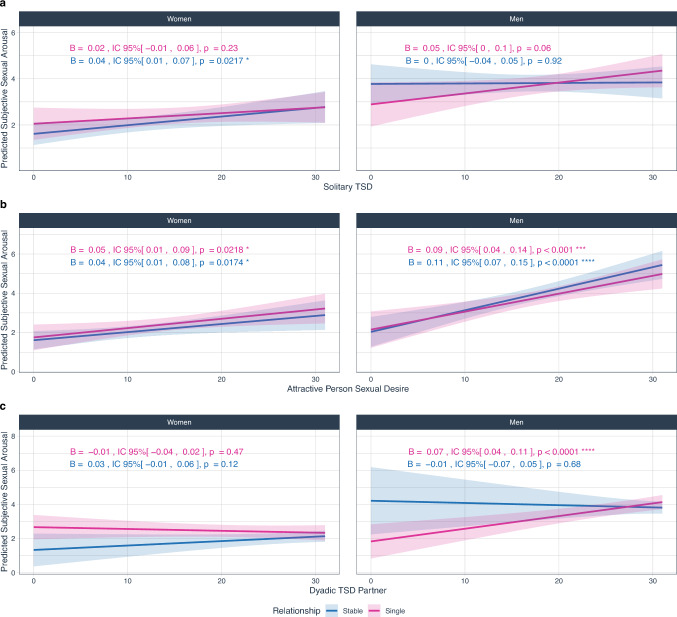
Slopes of trait sexual desire dimensions on subjective sexual arousal, by gender and relationship status. **a** Solitary trait sexual desire (for details, see Tables S30); **b** Dyadic trait sexual desire—attractive person (for details, see Tables S33); **c** Dyadic trait sexual desire—partner (for details, see Tables S35). Lines represent simple slopes and 95% CI. Significant effects are represented with stars alongside slope details: *p < .05, **p < .01, ***p < .001, ****p < .0001

When relationship status was included as a factor, the main effects of solitary TSD and dyadic TSD to an attractive person on SSA remained significant, whereas a three-way interaction involving relationship status was found only for the dyadic TSD dimension directed toward a partner (Table 3, Fig. 3).

**Table 3:** Effects of trait sexual desire dimensions on subjective sexual arousal across gender and relationship status

	Effect	LMM			*ϵ*^2^_p_
		df	*F*	*p*	
Solitary TSD	Solitary sexual desire	1315	6.88	**.01**	0.0183
	Gender	1355	14.1	**< .0001**	0.0335
	Relationship status	1315	0.3368	.56	< 0.0001
	Solitary sexual desire × Gender	1315	0.0712	.79	< 0.0001
	Solitary sexual desire × Relationship status	1315	0.5309	.47	< 0.0001
	Gender × Relationship status	1315	2.9531	**.09**	0.0061
	Solitary sexual desire × Gender × Relationship status	1315	2.0233	.16	0.0032
Dyadic TSD—attractive person	Dyadic TSD—Attractive Person (AP)	1315	46.8	**< .0001**	0.13
	Gender	1355	1.207	.27	< 0.0001
	Relationship status	1315	1.126	.72	< 0.0001
	Dyadic TSD Attractive person × Gender	1315	7.064	**.008**	0.0188
	Dyadic TSD Attractive person × Relationship status	1315	0.083	.77	< 0.0001
	Gender × Relationship status	1315	0.014	.97	< 0.0001
	Dyadic TSD Attractive person × Gender × Relationship status	1315	0.339	.56	< 0.0001
Dyadic TSD—partner	Dyadic TSD—Partner (P)	1312	3.163	.08	0.0069
	Gender	1328	2.500	.11	0.0045
	Relationship status	1312	0.670	.41	< 0.0001
	Dyadic TSD Partner × Gender	1312	1.153	.28	< 0.0001
	Dyadic TSD Partner × Relationship status	1312	1.374	.24	0.0012
	Gender × Relationship status	1312	8.505	**.004**	0.0234
	Dyadic TSD Partner × Gender × Relationship status	1312	8.308	**.004**	0.0228

Results are type III ANOVA. Solitary Trait Sexual Desire: *R*^*2*^_*conditional*_ = 0.72, *R*^*2*^_*marginal*_ = 0.171; Dyadic Trait Sexual Desire—Attractive Person: *R*^*2*^_*conditional*_ = 0.719, *R*^*2*^_*marginal*_ = 0.225; Dyadic Trait Sexual Desire—Partner: *R*^*2*^_*conditional*_ = 0.719, *R*^*2*^_*marginal*_ = 0.182. Gender = participants’ gender (women, men); Relationship = relationship type (stable, single). As effect size, we report partial epsilon squared (*ϵ*^*2*^_*p*_), which provides a less biased estimate than *η*^*2*^ (see Albers & Lakens, 2018). Significant effects are in bold

The association between solitary TSD and SSA (H3a) did not interact with participant gender or relationship status (Table 3). Simple slopes revealed that the slope of solitary TSD was significant for women in stable relationships (*B* = 0.04, 95% CIs [0.01, 0.07], *p* = .02), but not for single women (*B* = 0.02, 95% CIs [− 0.01, 0.06], *p* = .23), or men in a stable relationship (*B* = 0.002, 95% CIs [− 0.04, 0.05], *p* = .92); effects were marginally significant for single men (*B* = 0.05, 95% CIs [0.00, 0.10], *p* = .06; Fig. 3a).

Again, no significant interaction between dyadic TSD toward an attractive person, participant gender and relationship status were found (Table 3). The association between dyadic TSD toward an attractive person and SSA (H2b) were, however, significant in all cases; that is, a positive and significant slope was found for partnered women (*B* = 0.04, 95% CIs [0.01, 0.08], *p* = .02) and single women(*B* = 0.05, 95% CIs [0.01, 0.09], *p* = .02), and for partnered (*B* = 0.11, 95% CIs [0.07, 0.15], *p* < .0001), and single (*B* = 0.09, 95% CIs [0.04, 0.14], *p* < .001; Fig. 3b) men.

Finally, dyadic TSD toward a partner did significantly interact with participant gender and relationship status to predict SSA (H3c; Table 3). This interaction emerged because the slope of dyadic TSD showed a non-significant positive trend for women in stable relationships (*B* = 0.03, 95% CIs [− 0.01, 0.06], *p* = .12), but a non-significant negative trend for single women (*B* = − 0.01, 95% CIs [− 0.04, 0.02], *p* = .46). For men, these trends were reversed; the slope was showed a non-significant negative tendency for men in stable relationships (*B* = − 0.01, 95% CIs [− 0.07, 0.05], *p* = .68), but a significant and positive association for single men (*B* = 0.07, 95% CIs [0.04, 0.11], *p* < .001; Fig. 3c).

## Discussion

### Trait Sexual Desire: Gender Differences and Relationship Status

In the current study, we tested the association between TSD and SSA while examining how participant gender, stimulus gender, and relationship status moderated these association in cisgender, heterosexual individuals.

First, we assessed whether gender effects on TSD were modulated by relationship status (**H1**). We expected that all dimensions of TSD would be higher in men than in women but would vary by relationship status. The results supported our hypothesis; men reported higher levels of TSD than women on all dimensions (H1a, H1b and H1c), supporting previous studies reporting of gender effects in sexual motivation between men and women (Baumeister et al., 2001; Goldey & van Anders, 2012; Holmberg & Blair, 2009; Peixoto et al., 2020; Regan & Atkins, 2006; van Anders, 2012).

We also found that relationship status moderated the association between gender and solitary TSD, effects that had not been explored. While previous studies have indicated that solitary TSD tended to be higher in men (Peixoto, 2023), our results suggested that this gender effect holds only for people with partners, not in singles. This suggests that relationship dynamics play a key role in the expression of solitary TSD in men, while other factors may modulate the relationship between gender and solitary TSD among single individuals (Park & MacDonald, 2022).

One possible explanation for these effects is that, in heterosexual relationships, men often report higher levels of sexual desire than their women partners (Baumeister et al., 2001; van Anders et al., 2022), leading to discrepancies in desire that could result in greater solitary sexual activity as a compensatory mechanism (Carvalheira et al., 2015; Mark et al., 2014; Peixoto, 2019). In addition, gender norms around masturbation may contribute to these findings, as men tend to report more frequent solitary sexual activity even when in relationships, while women may experience greater social inhibition regarding self-stimulation (Kaestle & Allen, 2011). Our findings emphasize the importance of considering relationship status as a contextual moderator in studies of TSD and suggest that differences in solitary TSD are shaped not only by gender, but also by relational and socio-cultural factors (Dawson & Chivers, 2014a; Mark, 2015; van Anders et al., 2022).

### Trait Sexual Desire Linked to Subjective Sexual Arousal: Gender and Stimulus Gender Effects

Second, we examined the predictive association between dimensions of TSD and SSA and explored whether this association was gender-specific for men and gender-nonspecific for women (**H2**). Based on previous findings (Sierra et al., 2020), we expected a positive association between TSD toward an attractive person and SSA, but no significant relationship with the dimensions of solitary TSD or toward a partner. Contrary to expectations, all TSD dimensions positively predicted SSA towards erotic stimuli (H2a, H2b and H2c) and, furthermore, this association followed a gender-specific pattern, aligning with previous findings on category-specific sexual response in men and gender nonspecific effects in women (Dawson & Chivers, 2014b; Spape et al., 2014; Vásquez-Amézquita et al., 2019).

TSD toward a partner was not significantly associated with SSA in women, suggesting that women’s TSD in committed relationships may be less tied to immediate arousal responses and more influenced by relational and affective dynamics (Birnbaum et al., 2016). Conversely, the significant association observed in men toward stimuli depicting women suggests that men's dyadic TSD toward a partner remains more consistently linked to SSA elicited by the sexual stimuli (Dawson & Chivers, 2018; Timmers et al., 2018), and that this link persists regardless of whether they are in a committed relationship.

These findings contribute to a nuanced understanding of gender effects in the association between TSD and SSA among cisgender individuals, emphasizing the importance of considering both individual differences and contextual factors in sexual response models.

### Trait Sexual Desire Linked Subjective Sexual Arousal: Participant Gender and Relationship Status Moderation

Finally, we investigated whether the association between TSD dimensions and SSA was moderated by participant gender in interaction with relationship status (**H3**). We found that participant gender and relationship status moderated the association between TSD and SSA, although the extent and direction of this moderation varied by TSD dimension; a significant three-way interaction was found between dyadic TSD toward partner, participant gender, and relationship status.

In the association between solitary TSD and SSA (H3a), women in stable relationships with higher solitary TSD reported higher SSA, which might suggest that solitary TSD may contribute to managing desire discrepancies or sexual self-regulation within couples (Birnbaum et al., 2016; Cervilla et al., 2023; Mark, 2015). In contrast, single women showed no association, highlighting the role of relationship context. Single men showed a marginally significant relationship, which might indicate that men’s solitary desire becomes more prominent when there are no partnered sexual opportunities (Huang et al., 2022; Kaestle & Allen, 2011; van Anders et al., 2022).

Dyadic TSD toward an attractive person (H3b) was significantly associated with SSA across participant gender and relationship groups, with no interaction effects. This suggests that dyadic TSD consistently predicts SSA, regardless of relational context, contrasting with prior findings in smaller samples (Sierra et al., 2020). These results align with research showing that external sexual cues strongly activate sexual response mechanisms regardless of relationship status (Carvalho & Nobre, 2010). While men showed slightly stronger associations than women, the findings highlight a broad sexual responsiveness to potential sexual partners.

The most notable moderation effect emerged with dyadic TSD toward a partner (H3c), showing a significant interaction with participant gender and relationship status. Single men displayed a positive association with SSA, suggesting that partner-directed desire enhances arousal when unattached (Stone et al., 2011; Wisman & Thomas, 2022). In contrast, partnered men and women, regardless of relationship status, showed no significant associations. For partnered women, this aligns with research highlighting the influence of emotional closeness and relational satisfaction over external erotic stimuli (Birnbaum et al., 2016; Sierra et al., 2020).

Overall, these findings highlight the complexity of how TSD translates into sexual response, shaped by the interplay of individual traits, relational dynamics, and sociocontextual factors.

### Limitations and Strengths

This study’s online, anonymous design—amid the COVID-19 pandemic—limited the use of physiological (e.g., Dawson & Chivers, 2014b; Sierra et al., 2020; Timmers et al., 2018) or indirect sexual arousal measures (Bolmont et al., 2014, 2017, 2019), constraining inferences about the TSD-SSA relationship. Nonetheless, this format likely elicited more genuine responses by reducing social desirability bias (Milani et al., 2019; Sierra et al., 2018) and creating a more sexually relaxing environment than laboratory settings participants (Goldey & van Anders, 2012; Paterson et al., 2014). It may have also broadened participation, mitigating the typical sample bias of sexuality studies favoring sexually experienced individuals (Dawson & Chivers, 2019).

Importantly, online data collection minimized observer effects (Milani et al., 2019), potentially reducing gender effects on TSD and SSA. Women, often underrepresented in laboratory-based sexual research (Dawson & Chivers, 2019), may have felt more comfortable disclosing sexual responses in this format, decreasing cognitive biases and underreporting (Huang et al., 2022; Nobre, 2009; Stone et al., 2011), particularly among partnered women.

Lastly, the use of static stimuli may have influenced SSA responses, particularly in women, who are more sensitive to stimulus intensity and prepotency (Dawson & Chivers, 2018; Spape et al., 2014). Unlike sexual films, which reduce gender effects on SSA (Sierra et al., 2017, 2019), images may produce less differentiation between erotic and non-erotic stimuli, however in analyses of the effect of stimulus content, the strength of erotic stimuli in producing a subjective sexual arousal response was clear (Table S17, Figure S9).

While our sample was large compared to similar previous studies (Goldey & van Anders, 2012; Sierra et al., 2020), the inability to perform an a priori power analysis warrants cautious interpretation of non-significant effects, acknowledging potential limitations in statistical power.

Looking ahead, and given that our data and codes are openly available, future research could conduct a-priori power analyses, and also broaden the scope to include sexual and gender- diverse populations, and different types of relationships (e.g., exclusive, non-exclusive, etc.), which is essential to improve the generalizability of findings in studies examining individual differences such as gender and relational contexts (Mark et al., 2018; Peixoto et al., 2020).

Future research should incorporate physiological and comprehensive measures of sexual arousal (Cervilla et al., 2023; Sierra et al., 2017), explore influences on TSD like commitment levels (Rodrigues & Lopes, 2017), and examine how relational dynamics affect SSA. Addressing these directions may contribute to the development of comprehensive models of sexual motivation, fostering a more inclusive understanding of desire dynamics across relational and individual contexts.

### Conclusions

This study examined how trait sexual desire (TSD) relates to subjective sexual arousal (SSA) as a function of participant gender and relationship status across the three dimensions of TSD. The findings support the relationship between TSD and SSA (Sierra et al., 2020), moderated by individual differences such as gender and socio-contextual factors like relationship status. These relationships vary depending on the TSD dimension and seem to reflect a gender nonspecific pattern in heterosexual women and a gender-specific pattern in heterosexual men. Finally, we found that relationship status plays a significant role in modulating how TSD is expressed in SSA in response to erotic stimuli, particularly in the dyadic dimension directed toward a partner.

From a theoretical perspective, our results reinforce the notion that sexual desire is not a fixed trait but a dynamic and context-dependent construct. This aligns with the IMM, which posits that trait-like psychological predispositions influence how individuals process and respond to sexual stimuli (Toates, 2009), challenging traditional models and providing a foundation for future longitudinal research.

Our findings support the tridimensionality of TSD (Moyano et al., 2017) and extend the existing literature on its interaction with SSA (Sierra et al., 2020) across different gender and relational contexts, offering a more nuanced rather than generalized understanding of sexual desire as a trait.

This study highlights the importance of integrating trait-based perspectives on sexual desire, reinforcing the need for multidimensional models that consider personality traits, relational factors, and context-specific triggers.

These results also carry relevant clinical implications, highlighting the need for personalized assessments and interventions in addressing sexual desire concerns. Understanding how TSD varies across dimensions and is modulated by gender and relationship status can help clinicians explore desire discrepancies within couples more effectively, particularly in partner-focused dimensions. Recognizing sexual desire as dynamic rather than static may prevent the pathologization of normal variations in desire, allowing people to reframe their experiences within a broader context. Patterns observed in this study suggest that interventions should be sensitive to the diverse expressions of gender without confining them to rigid categories, acknowledging the complex interplay between sociocultural expectations and relational dynamics that influence sexual desire and arousal.

## Supplementary Information

Below is the link to the electronic supplementary material.

Supplementary file 1 (PDF 1673 KB)

## Data Availability

The data are openly accessible to the public from the Open Science Framework: 10.17605/OSF.IO/3V2E7.
