# Supplementary material for: Does Trait Sexual Desire Predict Subjective Sexual Response to Erotic Stimuli? Effects of Participant Gender, Stimulus Gender, and Relationship Status Among Cisgender Heterosexual Women and Men
Source: Arch Sex Behav. 2025 Dec 2;54(10):4209–23. doi: 10.1007/s10508-025-03298-w (PMC12753724; doi:10.1007/s10508-025-03298-w)
Supplement: Supplementary file 1 — (PDF 1673 KB) [file 10508_2025_3298_MOESM1_ESM.pdf]

Does Trait Sexual Desire Predict Subjective Sexual Response to Erotic Stimuli? Effects of Participant Gender, Stimulus Gender, and Relationship Status among Cisgender Heterosexual Women and Men

Code and analyses

Milena Vásquez-Amézquita [1,2,*] Marina Begoña Martínez-González [2]
Meredith L. Chivers [3] Juan David Leongómez [1]

21 August, 2025

[1] Faculty of Psychology, Universidad El Bosque, Bogota 110121, Colombia.
[2] Departamento de Ciencias Sociales, Universidad de la Costa, Barranquilla 080002, Colombia.
[3] Department of Psychology, Queen's University, Kingston ON K7L 3N6, Canada.

[*] Correspondence: mvasquezam@unbosque.edu.co

Description

This document contains all code, and step by step explanations for all analyses, figures and tables (including supplementary figures and tables) for:

Vásquez-Amézquita, M., Martínez-González, M. B., Chivers, M. L., & Leongómez, J. D. (2025). Does Trait Sexual Desire Predict Subjective Sexual Response to Erotic Stimuli? Effects of Participant Gender, Stimulus Gender, and Relationship Status among Cisgender Heterosexual Women and Men. *Archives of Sexual behavior.*

Data are available on the Open Science Framework (OSF): https://doi.org/10.17605/OSF.IO/3V2E7. The analyses were designed by Milena Vásquez-Amézquita and Juan David Leongómez. This document and its underlying code were created in R Markdown by Juan David Leongómez using R and LaTeX, ensuring full reproducibility. Code comments were refined with the assistance of R Wizard, a customized R-focused version of ChatGPT by OpenAI. More information at: https://exploregpt.ai/gpt/r-wizard-gpt.

Contents

1 Summary of the analytical approach **4**

2 Preliminaries **5**
2.1 Load packages . . . 5
2.2 Define color palettes . . . 5
2.3 Custom functions . . . 6
2.3.1 `pval.lev`, `pe2.lev` and `pval.stars` . . . 6
2.3.2 `corr.stars` . . . 6
2.3.3 `anova.sig.lm` and `anova.sig.lmer` . . . 7
2.3.4 `anova.comp` . . . 9
2.3.5 `contr.stars` . . . 10
2.4 Load and wrangle data . . . 10

3 Descriptives **13**
3.0.1 Figure S1. Demographic chacarteristics of the sample 13
3.1 Descriptive statistics of the participants by gender 15
3.1.1 Table S1. Descriptive statistics of the participants by gender 15
3.1.2 Figure S2. Distribution of participants' measured variables by gender 17
3.2 Correlations between measured variables 19
3.2.1 Table S2. Correlations between measured variables 19
3.3 Internal consistency 22
3.3.1 Table S3. Internal consistency of construct variables 23
3.4 Controlling for Relationship Duration and MGSS Sexual Satisfaction (Partner) in Sexual Desire Dimensions 24

4 Hypothesis tests **27**
4.1 Hypothesis 1: All dimensions of trait sexual desire (TSD) will be higher in men than in women, and the differences will be stronger or weaker according to relationship status 27
4.1.1 Data 27
4.1.2 Hypothesis 1a: Solitary TSD 28
4.1.2.1 Model the effects of relationship type and gender on Solitary TSD 28
4.1.2.1.1 Figure S3: Posterior predictive checks (PPCs) for Hypothesis 1a. 28
4.1.2.2 Table S5. ANOVA-type table for the interaction between `Relationship type`, and `Gender` 29
4.1.2.3 *Post-hoc* comparisons 29
4.1.2.3.1 Table S6. Estimated marginal means and contrasts between participants' gender. 29
4.1.2.3.2 Table S7. Estimated marginal means and contrasts between relationship status. 30
4.1.2.3.3 Table S8. Estimated marginal means and contrasts between gender by relationship status. 31
4.1.2.4 Figure S4. Effects of gender and relationship type on Solitary TSD 32
4.1.3 Hypothesis 1b: Dyadic TSD (Attractive person) 34
4.1.3.1 Model the effects of relationship type and gender on Dyadic TSD: Attractive person 34
4.1.3.1.1 Figure S5: Posterior predictive checks (PPCs) for Hypothesis 1b. 34
4.1.3.2 Table S9. ANOVA-type table for the interaction between `Relationship type`, and `Gender` 35
4.1.3.3 *Post-hoc* comparisons 36
4.1.3.3.1 Table S10. Estimated marginal means and contrasts between participants' gender. 36
4.1.3.3.2 Table S11. Estimated marginal means and contrasts between relationship status. 37
4.1.3.3.3 Table S12. Estimated marginal means and contrasts between gender by relationship status. 38
4.1.3.4 Figure S6. Effects of gender and relationship type on Dyadic TSD Attractive Person 39
4.1.4 Hypothesis 1c: Dyadic TSD (Partner) 40
4.1.4.1 Model the effects of relationship type and gender on Dyadic TSD: Partner 40
4.1.4.1.1 Figure S7: Posterior predictive checks (PPCs) for Hypothesis 1c. 41
4.1.4.2 Table S13. ANOVA-type table for the interaction between `Relationship type`, and `Gender` 42
4.1.4.3 *Post-hoc* comparisons 42
4.1.4.3.1 Table S14. Estimated marginal means and contrasts between participants' gender. 42
4.1.4.3.2 Table S15. Estimated marginal means and contrasts between relationship status. 43
4.1.4.3.3 Table S16. Estimated marginal means and contrasts between gender by relationship status. 44
4.1.4.4 Figure S8. Effects of gender and relationship type on Dyadic TSD Partner 45
4.2 Data filtering for hypotheses 2 and 3. 47

4.2.1 Table S17. ANOVA-type table for the effects of stimuli content, gender and stimuli gender on Subjective sexual arousal . . . 47
4.2.2 Table S18. Estimated marginal means and contrasts between subjective sexual arousal depending on stimuli gender, by stimuli content and participant gender. . . . 48
4.2.3 Figure S9. Effects of stimuli content (erotic, non-erotic) on subjective sexual arousal . . . 50
4.3 Hypothesis 2: The association between trait sexual desire (TSD) and subjective sexual arousal (SSA) will vary by TSD dimension, with these associations being gender-specific in men and gender-non-specific in women. . . . 52
4.3.1 Modeling Approach . . . 52
4.3.2 Data . . . 53
4.3.3 Hypothesis 2a: Solitary TSD . . . 53
4.3.3.1 Model Robustness: Examining the Effects of Solitary TSD on SSA Across Gender and Stimuli Gender . . . 53
4.3.3.1.1 Table S19. ANOVA-type table of fixed effects (main effects and interactions) across the three fitted models . . . 54
4.3.3.1.2 Figure S10: Model-based predictions for Hypothesis 2a. . . . 54
4.3.3.2 Final Model: Effects of Solitary TSD on SSA Across Gender and Stimuli Gender . . . 56
4.3.3.2.1 Table S20. ANOVA-type table for the interaction between `Relationship type`, and `Gender` . . . 56
4.3.3.2.2 *Post-hoc* tests . . . 57
4.3.3.3 Figure S11. Subjective sexual arousal to erotic stimuli: Main effects and interactions 58
4.3.4 Hypothesis 2b: Dyadic TSD Attractive Person . . . 59
4.3.4.1 Model Robustness: Examining the Effects of Dyadic TSD Attractive Person on SSA Across Gender and Stimuli Gender . . . 59
4.3.4.1.1 Table S22. ANOVA-type table of fixed effects (main effects and interactions) across the three fitted models . . . 60
4.3.4.1.2 Figure S12: Model-based predictions for Hypothesis 2b. . . . 60
4.3.4.2 Final Model: Effects of Dyadic TSD Attractive Person on SSA Across Gender and Stimuli Gender . . . 62
4.3.4.2.1 Table S23. ANOVA-type table for the interaction between `Relationship type`, and `Gender` . . . 63
4.3.4.2.2 *Post-hoc* tests . . . 63
4.3.4.3 Figure S13. Subjective sexual arousal to erotic stimuli: Main effects and interactions 64
4.3.5 Hypothesis 2c: Dyadic TSD Partner . . . 65
4.3.5.1 Model Robustness: Examining the Effects of Dyadic TSD Partner on SSA Across Gender and Stimuli Gender . . . 65
4.3.5.1.1 Table S25. ANOVA-type table of fixed effects (main effects and interactions) across the three fitted models . . . 66
4.3.5.1.2 Figure S14: Model-based predictions for Hypothesis 2c. . . . 66
4.3.5.2 Final Model: Effects of Dyadic TSD Partner on SSA Across Gender and Stimuli Gender . . . 68
4.3.5.2.1 Table S26. ANOVA-type table for the interaction between `Relationship type`, and `Gender` . . . 69
4.3.5.2.2 *Post-hoc* tests . . . 69
4.3.5.3 Figure S15. Subjective sexual arousal to erotic stimuli: Main effects and interactions 70
4.4 Hypothesis 3: The associations between TSD dimensions and SSA toward stimuli of self-reported preferred gender will be moderated by gender and relationship status. . . . 71
4.4.1 Modeling Approach . . . 72
4.4.2 Data . . . 72
4.4.3 Hypothesis 3a: Solitary TSD . . . 72
4.4.3.1 Model Robustness: Examining the Effects of Solitary TSD on SSA Across Gender and Stimuli Gender . . . 72
4.4.3.1.1 Table S28. ANOVA-type table of fixed effects (main effects and interactions) across the three fitted models . . . 73
4.4.3.1.2 Figure S16: Model-based predictions for Hypothesis 3a. . . . 74
4.4.3.2 Final Model: Effects of Solitary TSD on SSA Across Gender and Stimuli Gender . . . 75

4.4.3.2.1 Table S29. ANOVA-type table for the interaction between `Relationship type`, and `Gender` . . . 75
4.4.3.2.2 *Post-hoc* tests . . . 76
4.4.3.3 Figure S17. Subjective sexual arousal to erotic stimuli: Main effects and interactions 77
4.4.4 Hypothesis 3b: Dyadic TSD Attractive Person . . . 79
4.4.4.1 Model Robustness: Examining the Effects of Dyadic TSD Attractive Person on SSA Across Gender and Stimuli Gender . . . 79
4.4.4.1.1 Table S31. ANOVA-type table of fixed effects (main effects and interactions) across the three fitted models . . . 79
4.4.4.1.2 Figure S18: Model-based predictions for Hypothesis 3b. . . . 80
4.4.4.2 Final Model: Effects of Dyadic TSD Attractive Person on SSA Across Gender and Stimuli Gender . . . 82
4.4.4.2.1 Table S32. ANOVA-type table for the interaction between `Relationship type`, and `Gender` . . . 82
4.4.4.2.2 *Post-hoc* tests . . . 83
4.4.4.3 Figure S19. Subjective sexual arousal to erotic stimuli: Main effects and interactions 84
4.4.5 Hypothesis 3c: Dyadic TSD Partner . . . 85
4.4.5.1 Model Robustness: Examining the Effects of Dyadic TSD Partner on SSA Across Gender and Stimuli Gender . . . 85
4.4.5.1.1 Table S34. ANOVA-type table of fixed effects (main effects and interactions) across the three fitted models . . . 86
4.4.5.1.2 Figure S20: Model-based predictions for Hypothesis 3c. . . . 87
4.4.5.2 Final Model: Effects of Dyadic TSD Partner on SSA Across Gender and Stimuli Gender . . . 89
4.4.5.2.1 Table S35. ANOVA-type table for the interaction between `Relationship type`, and `Gender` . . . 89
4.4.5.2.2 *Post-hoc* tests . . . 89
4.4.5.3 Figure S21. Subjective sexual arousal to erotic stimuli: Main effects and interactions 90

5 Final figures and tables **91**
5.1 Table 1. Hypothesis 1 . . . 92
5.2 Figure 1. Hypothesis 1 . . . 93
5.3 Table 2. Hypothesis 2 . . . 94
5.4 Figure 2. Hypothesis 2 . . . 96
5.5 Table 3. Hypothesis 3 . . . 97
5.6 Figure 3. Hypothesis 3 . . . 99

6 Session info (for reproducibility) **100**

7 Supplementary references **101**

---

1 Summary of the analytical approach

This document presents the full statistical analyses conducted for the study, outlining the methodology, modeling approaches, and results for each hypothesis.

Hypothesis 1: Effects of Gender and Relationship Type on Trait Sexual Desire (TSD)

To examine differences in three dimensions of TSD (Solitary TSD, Dyadic TSD toward an attractive person, and Dyadic TSD toward a partner) as a function of gender and relationship status, we fitted linear models (LMs). These models included main effects and interaction terms, allowing for comparisons between men and women in stable relationships and single participants. Post-hoc comparisons were conducted where necessary, and effect sizes were estimated using partial epsilon squared.

Hypotheses 2 and 3: Associations Between TSD and Subjective Sexual Arousal (SSA)

To test the relationships between TSD dimensions and SSA, as well as their interactions with gender, relationship status, and stimulus characteristics, we employed three complementary modeling strategies to ensure robustness:

1. Cumulative Link Mixed Models (CLMM) for ordinal outcomes using a probit link function.
2. Generalized Linear Mixed Models (GLMM) with a Poisson family, treating SSA as count data.
3. Linear Mixed Models (LMM) approximating SSA as a continuous variable.

Key predictors included gender, relationship status, stimuli gender, and TSD dimensions, with interactions tested where applicable. Random effects were specified for stimuli and participants to account for within-subject dependencies. *Post-hoc* simple slope analyses were conducted to explore significant interactions, and effect sizes were estimated using partial epsilon squared.

---

2 Preliminaries

2.1 Load packages

This file was created using `knitr` (Xie, 2014), mostly using `tidyverse` (Wickham et al., 2019) syntax. As such, data wrangling was mainly done using packages such as `dplyr` (Wickham et al., 2023), and most figures were created or modified using `ggplot2` (Wickham, 2016). Tables were created using `knitr::kable` and `kableExtra` (Zhu, 2021).

Linear mixed models were fitted using `lmerTest` (Kuznetsova et al., 2017), cumulative link models using `ordinal` (Christensen, 2023), and generalized linear models were fitted using `lme4` (Bates et al., 2015). Assumptions were performed using `performance` (Lüdecke et al., 2021), contrasts and interactions were explored using `emmeans` (Lenth, 2023), and imple slopes were investigated using the package `interactions` (Long, 2019).

All packages used in this file can be directly installed from the Comprehensive R Archive Network (CRAN). For a complete list of packages used to create this file, and their versions, see section 6, at the end of the document.

```
library(readxl) # Read Excel files (.xls and .xlsx)
library(lme4) # Fit linear and generalized linear mixed-effects models
library(ordinal) # Analyze ordinal response data with cumulative link models
library(lmerTest) # Enhances lme4 by adding p-values for mixed-effects models
library(ltm) # Latent trait models, useful for item response theory (IRT)
library(car) # Companion to Applied Regression, includes various statistical tests
library(tidyquant) # Extends the tidyverse for quantitative financial analysis
library(performance) # Assesses model fit, assumptions, and performance diagnostics
library(kableExtra) # Enhances `kable()` for creating stylish tables in reports
library(psych) # Tools for psychological and psychometric analysis
library(scales) # Scaling functions for visualization (e.g., rescaling axes)
library(emmeans) # Estimated marginal means (EMMs) for contrasts and post-hoc comparisons
library(berryFunctions) # Various utility functions (e.g., data manipulation, visualization)
library(bestNormalize) # Implements normalization techniques for data transformation
library(rstatix) # Streamlines statistical tests and effect size calculations
library(effectsize) # Computes effect sizes, confidence intervals, and standardized measures
library(ggpubr) # Publication-ready figures, extends `ggplot2`
library(interactions) # Facilitates interaction plots and effect visualization
library(tidyverse) # Data manipulation and visualization packages (e.g., dplyr, ggplot2)
```

2.2 Define color palettes

Individual color palettes for figures by gender, stimuli gender, or relationship type.

```
# Palette to color figures by gender
color.Gender <- c("red", "black")
# Palette to color figures by stimuli gender
color.StimuliSex <- c("#54278F", "#FC4E2A")
# Palette to color figures by relationship type
color.Relationship <- c("#2171B5", "#DD3497")
```

```
# Palette to color figures by stimuli content
color.Content <- c("#41AB5D", "navyblue")
```

2.3 Custom functions

2.3.1 pval.lev, pe2.lev and pval.stars

This functions take p-values and epsilon squared effect sizes and formats them in LaTeX, highlighting significant p-values in bold and representing all in an appropriate level.

```
# Function to format p-values for LaTeX output, highlighting significant values in bold
pval.lev <- function(pvals) {
  ifelse(pvals < 0.0001, "\\textbf{< 0.0001}", # Highlight very small p-values
    ifelse(pvals < 0.001, "\\textbf{< 0.001}", # Bold p-values < 0.001
      ifelse(pvals < 0.05, paste0("\\textbf{", round(pvals, 4), "}"), # Bold p-values < 0.05
        round(pvals, 2) # Round non-significant values to two decimal places
      )
    )
  )
}

# Function to format partial epsilon squared (pe2) values for reporting
pe2.lev <- function(pvals) {
  ifelse(pvals < 0.0001, "< 0.0001", # Represent very small values as "< 0.0001"
    ifelse(pvals < 0.001, "< 0.001", # Represent values < 0.001 in scientific format
      ifelse(pvals < 0.05, round(pvals, 4), # Round values < 0.05 to four decimal places
        round(pvals, 2) # Round all other values to two decimal places
      )
    )
  )
}

# Function to add significance stars based on p-value thresholds
pval.stars <- function(pvals) {
  ifelse(pvals < 0.0001, "****", # Four stars for p < 0.0001
    ifelse(pvals < 0.001, "***", # Three stars for p < 0.001
      ifelse(pvals < 0.01, "**", # Two stars for p < 0.01
        ifelse(pvals < 0.05, "*", NA) # One star for p < 0.05, NA otherwise
      )
    )
  )
}
```

2.3.2 corr.stars

This function creates a correlation matrix, and displays significance (function `corr.stars` modified from http://myowelt.blogspot.com/2008/04/beautiful-correlation-tables-in-r.html).

```
# Function to create a correlation matrix with significance levels in LaTeX format
corr.stars <- function(x) {
  require(Hmisc) # Load Hmisc package for correlation and p-value calculations
  x <- as.matrix(x) # Ensure input is a matrix
  R <- rcorr(x)$r # Compute correlation coefficients
  p <- rcorr(x)$P # Extract p-values for significance testing
  # Define symbols for significance levels, using LaTeX formatting for bold and stars
  mystars <- ifelse(p < .001, paste0("\\textbf{", round(R, 2), "***}"), # p < 0.001
    ifelse(p < .01, paste0("\\textbf{", round(R, 2), "**}"), # p < 0.01
      ifelse(p < .05, paste0("\\textbf{", round(R, 2), "*}"), # p < 0.05
```

```
        ifelse(p < .10, paste0(round(R, 2), "$^{\\dagger}$"), # p < 0.10 (trend level)
          format(round(R, 2), nsmall = 2) # Format non-significant values with two decimals
        )
      )
    )
  )
  # Construct a new matrix with correlation values and significance symbols
  Rnew <- matrix(mystars, ncol = ncol(x))
  # Ensure diagonal values remain the original correlation values (without significance symbols)
  diag(Rnew) <- paste(diag(R), " ", sep = "")
  # Assign row and column names for the formatted matrix
  rownames(Rnew) <- colnames(x)
  colnames(Rnew) <- paste(colnames(x), "", sep = "")
  # Remove the upper triangle of the matrix (including the diagonal) for a clean presentation
  Rnew <- as.matrix(Rnew)
  Rnew[upper.tri(Rnew, diag = TRUE)] <- ""
  # Convert to a data frame for better handling and remove the last empty column
  Rnew <- as.data.frame(Rnew)
  Rnew <- cbind(Rnew[1:length(Rnew) - 1])
  return(Rnew) # Return formatted correlation table
}
```

2.3.3 `anova.sig.lm` and `anova.sig.lmer`

Functions to bold significant *p* values from `anova` or `car:Anova` model tables. It highlights significant *p* values, and formats the output in LaTeX, ready to be used with `kable`.

```
# Function to format ANOVA results for linear models (lm) in LaTeX format
anova.sig.lm <- function(model, custom_caption) {
  aovTab <- bind_cols(
    anova_summary(Anova(model, type = 3)), # Perform Type III ANOVA
    epsilon_squared(model) # Compute partial epsilon squared effect sizes
  ) |>
    unite(col = "df", DFn:DFd, sep = ", ") |> # Combine numerator and denominator df
    select(Effect, df, F, p, Epsilon2_partial) |> # Select relevant columns
    mutate(
      p = pval.lev(p), # Format p-values in LaTeX, bolding significant ones
      Epsilon2_partial = pe2.lev(Epsilon2_partial) # Format epsilon squared values
    ) |>
    mutate_at("Effect", str_replace_all, ":", " × ") |> # Replace ":" with "×" for LaTeX
    kable(
      digits = 2, booktabs = TRUE, align = c("l", rep("c", 4)),
      linesep = "",
      caption = custom_caption,
      col.names = c("Effect", "$df$", "$F$", "$p$", "$\\epsilon^2_p$"),
      escape = FALSE
    ) |>
    kable_styling(latex_options = c("HOLD_position", "scale_down")) |>
    footnote(
      general = paste0(
        "Sexual desire was transformed using an ordered quantile normalization ",
        "(\\\\cite{petersonOrderedQuantileNormalization2020a}). ",
        "Results are Type III ANOVA. ",
        "$R^2$ = ", round(r2(model)$R2, 3),
        ", $R^2_{adjusted}$ = ", round(r2(model)$R2_adjusted, 3), ". ",
        "Gender = participant's gender (women, men); ",
        "Relationship = relationship type (stable, single). ",
```

```
        "As effect size, we report partial epsilon squared ",
        "($\\\\epsilon^2_p$), a less biased estimate than $\\\\eta^2$ ",
        "(see \\\\cite{albersWhenPowerAnalyses2018}). ",
        "Significant effects are in bold."
      ),
      escape = FALSE, threeparttable = TRUE, footnote_as_chunk = TRUE
    )
  return(aovTab)
}

# Function to format ANOVA results for linear mixed models (lmer) in LaTeX format
anova.sig.lmer <- function(model, custom_caption) {
  aovTab <- bind_cols(
    anova(model), # Perform ANOVA on the mixed-effects model
    epsilon_squared(model) # Compute partial epsilon squared effect sizes
  ) |>
    mutate(DenDF = round(DenDF, 2)) |> # Round denominator degrees of freedom
    unite(col = "df", NumDF:DenDF, sep = ", ") |> # Combine numerator & denominator df
    rownames_to_column(var = "Effect") |> # Convert row names to a column
    rename(
      "F" = "F value", # Rename columns for consistency
      "p" = "Pr(>F)"
    ) |>
    select(Effect, df, F, p, Epsilon2_partial) |> # Select relevant columns
    mutate(
      p = pval.lev(p), # Format p-values in LaTeX, bolding significant ones
      Epsilon2_partial = pe2.lev(Epsilon2_partial) # Format epsilon squared values
    ) |>
    mutate(Effect = str_replace_all(Effect, "\\.", " ")) |> # Replace dots with spaces
    mutate(Effect = str_replace_all(Effect, ":", " × ")) |> # Replace ":" with "×" for LaTeX
    mutate(Effect = str_remove_all(Effect, "`")) |> # Remove "`"
    kable(
      digits = 2, booktabs = TRUE, align = c("l", rep("c", 4)),
      linesep = "",
      caption = custom_caption,
      col.names = c("Effect", "$df$", "$F$", "$p$", "$\\epsilon^2_p$"),
      escape = FALSE
    ) |>
    kable_styling(latex_options = c("HOLD_position", "scale_down")) |>
    footnote(
      general = paste0(
        "Results are Type III ANOVA. ",
        "$R^2_{conditional}$ = ", round(r2_nakagawa(model)$R2_conditional, 3),
        ", $R^2_{marginal}$ = ", round(r2_nakagawa(model)$R2_marginal, 3), ". ",
        "As effect size, we report partial epsilon squared ",
        "($\\\\epsilon^2_p$), a less biased estimate than $\\\\eta^2$ ",
        "(see \\\\cite{albersWhenPowerAnalyses2018}). ",
        "Significant effects are in bold."
      ),
      escape = FALSE, threeparttable = TRUE, footnote_as_chunk = TRUE
    )
  return(aovTab)
}
```

2.3.4 `anova.comp`

Function to compare ANOVA-type tables from hypotheses 2 and 3 models fitted with different techniques: `ordinal::clmm` (Cumulative Link Mixed Models, CLMM), `lme4::glmer` (Generalized Linear Mixed-Effects Models; GLMER) and `lmerTest::lmer` (Linear Mixed-Effects Models, LMER). The function bold significant p values from `anova` or `car:Anova` tables. It highlights significant p values, and formats the output in LaTeX, ready to be used with `kable`.

```
# Function to compare ANOVA results across CLMM, GLMER (Poisson), and LMM models
anova.comp <- function(CLMMmod, GLMERmod, LMERmod, hypothesis) {
  compTab <-
    reduce(
      list(
        # ANOVA results for the Cumulative Link Mixed Model (CLMM)
        Anova(CLMMmod, type = 3) |>
          as.data.frame() |>
          mutate(`Pr(>Chisq)` = pval.lev(`Pr(>Chisq)`)) |> # Format p-values
          rownames_to_column("Effect"),
        # ANOVA results for the Generalized Linear Mixed Model (GLMM, Poisson),
        # selecting relevant columns & removing the intercept row
        Anova(GLMERmod, type = 3) |>
          as.data.frame() |>
          mutate(`Pr(>Chisq)` = pval.lev(`Pr(>Chisq)`)) |> # Format p-values
          rownames_to_column("Effect") |>
          select(Effect, Df, Chisq, `Pr(>Chisq)`) |> # Keep relevant columns
          slice_tail(n = -1), # Remove intercept row
        # ANOVA results for the Linear Mixed Model (LMM),
        # rounding denominator DF and formatting df column
        anova(LMERmod, type = 3) |>
          as.data.frame() |>
          rownames_to_column("Effect") |>
          mutate(
            DenDF = round(DenDF, 2), # Round denominator df
            `Pr(>F)` = pval.lev(`Pr(>F)`) # Format p-values
          ) |>
          unite(col = "df", NumDF:DenDF, sep = ", ") |> # Combine numerator & denominator df
          select(Effect, df, `F value`, `Pr(>F)`) # Keep relevant columns
      ),
      full_join,
      by = "Effect" # Merge tables by "Effect"
    ) |>
    # Improve readability of effect names
    mutate(Effect = str_replace_all(Effect, "\\.", " ")) |> # Replace dots with spaces
    mutate(Effect = str_replace_all(Effect, ":", " × ")) |> # Replace colons with × for LaTeX
    # Generate formatted table
    kable(
      booktabs = TRUE, align = c("l", rep("c", 9)), digits = 3,
      linesep = "",
      caption = paste0(
        "Comparison of fixed effects across the three models for Hypothesis ",
        hypothesis, ": CLMM, GLMM (Poisson), and LMM."
      ),
      col.names = c(
        "Effect", rep(c("$df$", "$\\chi^2$", "$p$"), times = 2), "$df$", "$F$", "$p$"
      ),
      escape = FALSE
    ) |>
```

```
    # Apply LaTeX styling
    kable_styling(latex_options = c("HOLD_position", "scale_down")) |>
    # Add section headers for each model
    add_header_above(c(
      " " = 1, "CLMM" = 3, "GLMER (Poisson)" = 3, "LMM" = 3
    )) |>
    # Add footnote explaining statistical details
    footnote(
      general = "For CLMM and GLMER (Poisson) models, results are
              Analysis of Deviance (Type III Wald chi-square tests),
              while for LMM, results are from an Analysis of Variance
              (Type III ANOVA with Satterthwaite's method).
              Significant effects are in bold.",
      threeparttable = TRUE, footnote_as_chunk = TRUE, escape = FALSE
    )
  return(compTab) # Return the formatted comparison table
}
```

2.3.5 `contr.stars`

Function to create a data frame of model contrasts, representing significance levels from an `emmeans::emmeans` output. These data frames are formatted to be called by the `ggpubr::stat_pvalue_manual` function used in model figures.

```
# Function to format contrast results from emmeans for ggpubr::stat_pvalue_manual
contr.stars <- function(emms) {
  require(emmeans) # Load emmeans package for pairwise comparisons
  # Compute pairwise contrasts and convert output to a data frame
  x <- as.data.frame(contrast(emms, interaction = "pairwise"))
  # Separate the first column into two groups (group1 and group2)
  x <- separate(
    x,
    col = 1, into = c("group1", "group2"), sep = " - ", remove = TRUE
  )
  # Assign significance stars based on p-value thresholds
  x$p.signif <- ifelse(x$p.value < 0.0001, "****", # p < 0.0001
    ifelse(x$p.value < 0.001, "***", # p < 0.001
      ifelse(x$p.value < 0.01, "**", # p < 0.01
        ifelse(x$p.value < 0.05, "*", NA) # p < 0.05, otherwise NA
      )
    )
  )
  # Remove parentheses from group names for cleaner labeling
  x <- x |>
    mutate_at("group1", str_replace_all, "[()]", "") |>
    mutate_at("group2", str_replace_all, "[()]", "")
  return(x) # Return formatted contrast data frame
}
```

2.4 Load and wrangle data

Change necessary variables to factor, sort levels, and rename variables

```
# Load data
dat <- read.csv("data/BD_Heterosexuales_Vertical_BIG.csv") |>
  # Remove rows with missing values for Solitary sexual desire (SD_solitario)
  drop_na(SD_solitario) |>
```

```r
# Change variables to factor and sort their levels
mutate_at(c(
  "Contenido_Estimulo", "Sexo", "Sexo_Estimulo", "PrefSex", "EstRel", "Escolaridad",
  "Religion", "TiempoRP"
), as.factor) |>
# Rename variables to English
rename(
  Participant = Participante,
  Age = EdadParticipante,
  `Preferred gender` = PrefSex,
  Gender = Sexo,
  `Contraceptive uso` = Anticoncep,
  `Last period` = UltimoPer,
  `Period day` = Dia_ciclo,
  Education = Escolaridad,
  Location = Residencia,
  `Location (other)` = Residencia_3_TEXT,
  `Medical history` = AntMed,
  `Sexual orientation` = OS,
  `Relationship status` = EstRel,
  `Relationship duration` = TiempoRP,
  `Partner gender` = SexPareja,
  `Relationship type` = TipoRel,
  `Age at first intercourse` = Primera.ExpSex,
  `Consented to first intercourse` = ConExpSex,
  `Number of sexual partners` = Numero.Parejas,
  `Pornography consumed last month` = Pornografia_ultimo_mes,
  Relationship = TieneRelacion,
  `MGH-SFQ (total)` = MGH.SFQ_Total,
  `Dyadic sexual desire (Partner)` = SD_Diadico_pareja,
  `Solitary sexual desire` = SD_solitario,
  `Dyadic sexual desire (Attractive person)` = SD_Diadico_p_atractiva,
  `MGSS sexual satisfaction (General)` = Satisfaccion.Sexual..MGSS_general.,
  `MGSS sexual satisfaction (Partner)` = Satisfaccion.Sexual..MGSS_Pareja.,
  `Stimuli code` = Codigo_Estimulo,
  `Stimuli gender` = Sexo_Estimulo,
  `Stimuli content` = Contenido_Estimulo,
  `Subjective sexual attractiveness` = Atractivo,
  `Subjective sexual arousal` = Excitacion
) |>
# Recode factor levels
mutate(`Stimuli content` = recode_factor(`Stimuli content`,
  Erotico = "Erotic",
  No_erotico = "Non-erotic"
)) |>
mutate(Gender = recode_factor(Gender,
  Femenino = "Women",
  Masculino = "Men"
)) |>
mutate(`Stimuli gender` = recode_factor(`Stimuli gender`,
  Femenino = "Female",
  Masculino = "Male"
)) |>
mutate(`Preferred gender` = recode_factor(`Preferred gender`,
  Hombre = "Male",
  Mujer = "Female"
```

```
)) |>
mutate(Education = recode(Education,
  "Bachillerato" = "High school",
  "Universitario" = "University",
  "Postgrado" = "Postgraduate"
)) |>
mutate(Religion = recode(Religion,
  "1" = "Religious",
  "0" = "Non-religious"
)) |>
mutate(`Pornography consumed last month` = recode(`Pornography consumed last month`,
  "Nunca" = "None",
  "Una o dos veces" = "1-2 times",
  "Tres a cinco veces" = "3-5 times",
  "Mas de 5 veces" = "5 times or more"
)) |>
# Recode relationship duration
# mutate(`Relationship duration` = replace_na(`Relationship duration`, "Single"))
mutate(
  `Relationship duration` = recode(`Relationship duration`,
    "Sin pareja actual" = "Single",
    "Menor a 6 meses" = "Less that 6 months",
    "Entre 6 meses y 2 anos" = "Between 6 months and 2 years",
    "Entre 2 y 5 anos" = "Between 2 and 5 years",
    "MÃ¡s de 5 anos" = "More than 5 years"
  ),
  `Relationship duration` = replace_na(`Relationship duration`, "Single")
) |>
# Recode relationship type
mutate(Relationship = recode(`Relationship status`,
  "Exclusiva/No viven juntos" = "Stable",
  "Exclusiva/Matrimonio" = "Stable",
  "No exclusiva" = "Non-stable",
  "Soltero/sin contactos sexuales en un ano" = "Single",
  "Soltero/contactos sexuales en un ano" = "Single"
)) |>
# Relevel factors
mutate(
  Education = fct_relevel(
    Education,
    c("High school", "University", "Postgraduate")
  ),
  `Pornography consumed last month` = fct_relevel(
    `Pornography consumed last month`,
    c(
      "None", "1-2 times",
      "3-5 times", "5 times or more"
    )
  ),
  `Relationship duration` = fct_relevel(
    `Relationship duration`,
    c(
      "Single", "Less that 6 months",
      "Between 6 months and 2 years",
      "Between 2 and 5 years",
      "More than 5 years"
```

```
      )
    )
  ) |>
  mutate(
    `Stimuli content` = as.factor(`Stimuli content`),
    `Stimuli gender` = as.factor(`Stimuli gender`)
  ) |>
  # Filter participants in non-stable relationships
  filter(Relationship != "Non-stable") |>
  droplevels()
```

---

3 Descriptives

3.0.1 Figure S1. Demographic chacarteristics of the sample

Number of participants by demographic category.

```
# Prepare data for demographic characteristics plot
dat.demog <- dat |>
  select(
    Participant, Gender, Relationship, Education, Religion, `Pornography consumed last month`
  ) |>
  group_by(Participant) |>
  filter(row_number() == 1) |> # Keep only one row per participant
  ungroup() |>
  group_by(Gender, Relationship, Education, Religion, `Pornography consumed last month`) |>
  rename(Porn = `Pornography consumed last month`) |> # Shorten column name
  tally() |> # Count occurrences
  drop_na(Religion) |> # Remove missing values in Religion column
  ungroup()

# Create separate datasets by gender
dat.demog.W <- filter(dat.demog, Gender == "Women") # Women data
dat.demog.M <- filter(dat.demog, Gender == "Men") # Men data

# Plot for Women
samp.w <- ggballoonplot(dat.demog.W,
  x = "Education", y = "Porn", size = "n", fill = "n",
  facet.by = c("Relationship", "Religion") # Separate facets by relationship & religion
) +
  scale_fill_viridis_c(option = "C", limits = c(1, max(dat.demog$n))) + # Color scale
  scale_size_continuous(range = c(1, 7), limits = c(1, max(dat.demog$n))) + # Size scale
  guides(fill = guide_legend(face = "italic"), size = guide_legend(face = "italic")) +
  labs(title = "Women", y = "Pornography consumed last month") + # Labels
  geom_text(aes(label = n), size = 3, nudge_x = 0.3, nudge_y = 0.1) + # Add counts
  geom_text(
    aes(label = paste0("\n(", percent(n / sum(dat.demog$n), accuracy = 0.1), ")")),
    size = 2.5, nudge_x = 0.3, nudge_y = -0.05 # Add percentage below counts
  ) +
  theme_tq() + # Apply tidyquant theme
  theme(
    axis.text.x = element_text(angle = 45, hjust = 1), # Rotate x-axis text
    axis.text.y = element_text(angle = 45, vjust = 0.5) # Rotate y-axis text
  )
```

```
# Plot for Men
samp.m <- ggballoonplot(dat.demog.M,
  x = "Education", y = "Porn", size = "n", fill = "n",
  facet.by = c("Relationship", "Religion") # Separate facets by relationship & religion
) +
  scale_fill_viridis_c(option = "C", limits = c(1, max(dat.demog$n))) + # Color scale
  scale_size_continuous(range = c(1, 7), limits = c(1, max(dat.demog$n))) + # Size scale
  guides(fill = guide_legend(face = "italic"), size = guide_legend(face = "italic")) +
  labs(title = "Men", y = NULL) + # Labels
  geom_text(aes(label = n), size = 3, nudge_x = 0.3, nudge_y = 0.1) + # Add counts
  geom_text(
    aes(label = paste0("\n(", percent(n / sum(dat.demog$n), accuracy = 0.1), ")")),
    size = 2.5, nudge_x = 0.3, nudge_y = -0.05 # Add percentage below counts
  ) +
  theme_tq() + # Apply tidyquant theme
  theme(
    axis.text.x = element_text(angle = 45, hjust = 1), # Rotate x-axis text
    axis.text.y = element_text(angle = 45, vjust = 0.5) # Rotate y-axis text
  )

# Combine Women & Men plots into a single figure
ggarrange(
  samp.w, samp.m,
  widths = c(1.1, 1), # Adjust panel widths
  common.legend = TRUE, legend = "bottom" # Use a common legend at the bottom
)
```

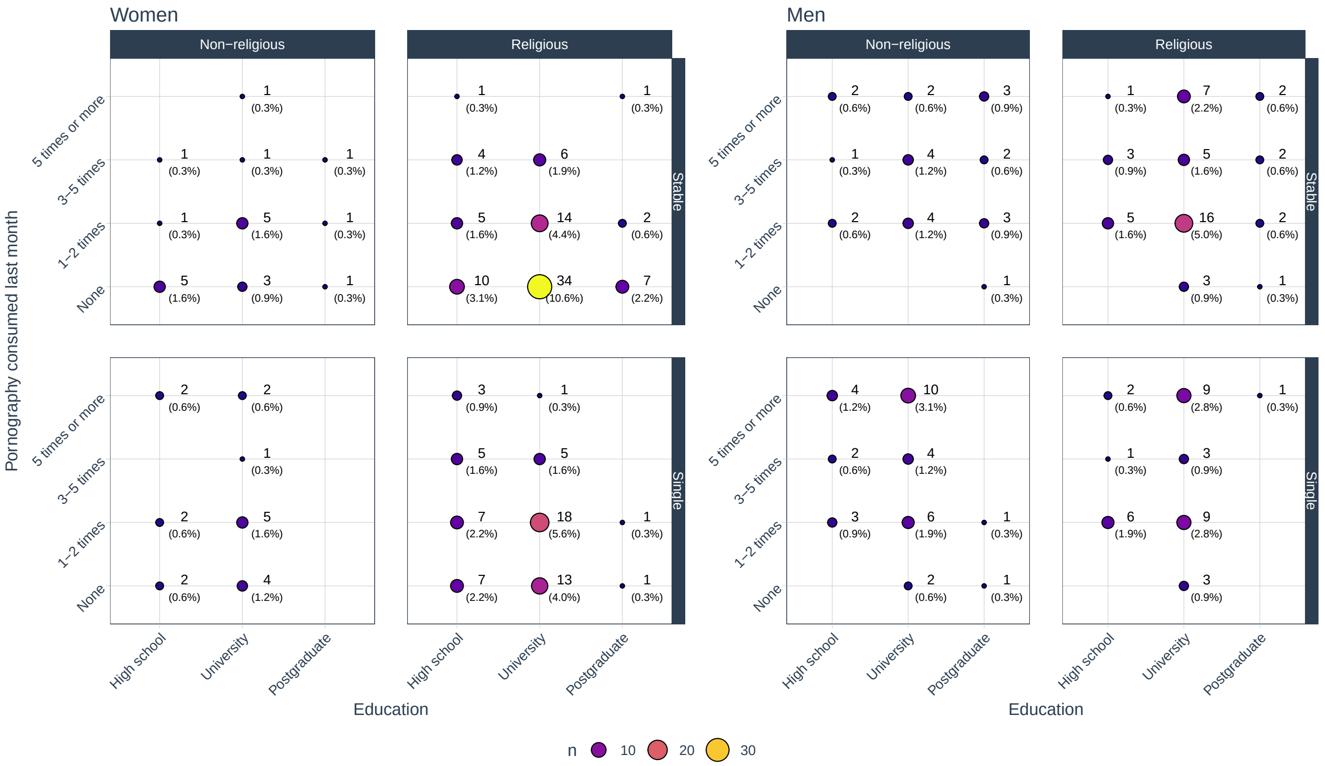

Figure S1. Number of participants by gender (left = women, rigth = men), Relationship (stable = top panels, single = bottom panels), Religion (non-religious = left panels by gender, religious = right panels by gender), Education (X axis), and pornography consumed during the last month (Y axis). The number of participants for each combination of these five variables is displayed as numbers (percentage in brackets), as well as by the color and size of the bubbles .

3.1 Descriptive statistics of the participants by gender

Calculate mean values per participant for relevant, numeric variables.

```
# Compute mean values per participant for selected numeric variables
dat.desc <- dat |>
  select(
    Participant, Gender, Age, Relationship, `Number of sexual partners`,
    `MGH-SFQ (total)`,
    `MGSS sexual satisfaction (General)`, `MGSS sexual satisfaction (Partner)`,
    `Subjective sexual attractiveness`, `Subjective sexual arousal`,
    `Solitary sexual desire`,
    `Dyadic sexual desire (Attractive person)`, `Dyadic sexual desire (Partner)`
  ) |>
  group_by(Participant, Gender, Relationship) |> # Group by participant, gender, relationship
  summarize_if(is.numeric, mean, na.rm = TRUE) # Compute mean for numeric variables
```

3.1.1 Table S1. Descriptive statistics of the participants by gender

Table of descriptives by gender.

```
# Generate a table of descriptive statistics by gender and relationship status
describeBy(dat.desc ~ Relationship + Gender,
  mat = TRUE, digits = 2 # Convert output to a matrix, round to 2 decimal places
) |>
  rownames_to_column("Measured characteristic") |> # Move row names to a column
  select(1, 3:4, 6:9, 12:13) |> # Select relevant columns
```

```
slice(-(1:12)) |> # Remove unnecessary rows
select(1, 3, 2, 4:9) |> # Reorder columns for better readability
# Remove numeric suffixes from row names (now in the "Measured characteristic" column)
mutate("Measured characteristic" = str_replace_all(
  `Measured characteristic`, c("1" = "", "2" = "", "3" = "", "4" = "")
)) |>
# Create formatted table
kable(
  digits = 2, booktabs = TRUE, align = c("l", "l", rep("c", 7)),
  linesep = "",
  caption = "Descriptive statistics of the participants by gender and relationship status",
  col.names = c(
    "Measured characteristic", "Gender", "Relationship status",
    "$n$", "Mean", "$SD$", "Median", "Min", "Max"
  ),
  longtable = TRUE, escape = FALSE
) |>
# Apply LaTeX styling
kable_styling(latex_options = c("HOLD_position"), font_size = 8.2) |>
# Collapse duplicate values in the first three columns for clarity
collapse_rows(columns = 1:3, valign = "middle") |>
# Add a footnote explaining within-subject descriptives
footnote(
  general = "Because for \\\\textit{Subjective sexual attractiveness} and
         \\\\textit{Subjective sexual arousal}, there are multiple within-subject
         observations, descriptives are calculated from mean values per participant.",
  threeparttable = TRUE, footnote_as_chunk = TRUE, escape = FALSE
)
```

Table S1. *Descriptive statistics of the participants by gender and relationship status*

Measured characteristic	Gender	Relationship status	n	Mean	SD	Median	Min	Max
Age	Women	Stable	105	24.51	5.58	23.00	18.00	40.00
		Single	79	22.27	3.84	21.00	18.00	36.00
	Men	Stable	72	26.72	5.64	25.00	19.00	40.00
		Single	67	24.24	4.58	23.00	18.00	39.00
Number of sexual partners	Women	Stable	103	4.41	3.77	3.00	1.00	22.00
		Single	76	5.74	8.85	3.00	0.00	63.00
	Men	Stable	72	8.72	11.36	5.00	1.00	70.00
		Single	66	7.30	8.06	4.00	0.00	40.00
MGH-SFQ (total)	Women	Stable	104	3.31	0.96	3.75	0.00	4.00
		Single	79	2.80	1.23	3.50	0.00	4.00
	Men	Stable	72	3.59	0.62	3.90	0.60	4.00
		Single	67	3.38	0.83	3.80	0.60	4.00
MGSS sexual satisfaction (General)	Women	Stable	100	25.88	5.67	28.00	6.00	30.00
		Single	10	26.90	3.11	27.00	22.00	30.00
	Men	Stable	70	26.43	4.54	29.00	12.00	30.00
		Single	12	23.58	5.14	24.50	14.00	29.00
MGSS sexual satisfaction (Partner)	Women	Stable	100	28.13	4.20	30.00	8.00	30.00
		Single	10	28.10	2.13	29.00	25.00	30.00
	Men	Stable	70	28.49	3.48	30.00	6.00	30.00
		Single	12	26.08	4.85	27.50	15.00	30.00

Subjective sexual attractiveness	Women	Stable	105	2.94	1.11	2.78	1.00	5.49
		Single	79	3.19	1.06	3.11	1.44	6.77
	Men	Stable	72	3.27	0.94	3.24	1.11	6.20
		Single	67	3.20	0.90	3.18	1.09	5.72
Subjective sexual arousal	Women	Stable	105	1.59	0.68	1.39	1.00	4.21
		Single	79	1.75	0.71	1.52	1.00	4.39
	Men	Stable	72	2.24	0.83	2.07	1.00	4.57
		Single	67	2.16	0.78	2.05	1.00	4.09
Solitary sexual desire	Women	Stable	105	11.53	8.59	12.00	0.00	29.00
		Single	79	16.03	8.35	17.00	0.00	31.00
	Men	Stable	72	17.47	7.51	17.50	0.00	31.00
		Single	67	18.25	7.10	19.00	1.00	31.00
Dyadic sexual desire (Attractive person)	Women	Stable	105	10.55	7.64	10.00	0.00	30.00
		Single	79	14.06	7.39	15.00	0.00	32.00
	Men	Stable	72	16.21	7.44	15.50	0.00	32.00
		Single	67	17.57	6.66	17.00	2.00	30.00
Dyadic sexual desire (Partner)	Women	Stable	105	27.53	8.50	30.00	0.00	38.00
		Single	76	21.33	10.91	23.00	0.00	38.00
	Men	Stable	72	31.35	5.33	32.00	15.00	38.00
		Single	67	25.81	9.40	28.00	0.00	38.00

Note: Because for *Subjective sexual attractiveness* and *Subjective sexual arousal*, there are multiple within-subject observations, descriptives are calculated from mean values per participant.

3.1.2 Figure S2. Distribution of participants' measured variables by gender

Kernel density distributions by gender.

```
# Convert dat.desc to long format for easier plotting
datp <- dat.desc |>
  pivot_longer(
    cols = Age:`Dyadic sexual desire (Partner)`, # Convert selected columns to long format
    names_to = "Variable", values_to = "Value"
  ) |>
  mutate(Variable = str_wrap(Variable, width = 30)) # Wrap variable names for better display

# Panel 1: Age, Sexual Partners, and Subjective Sexual Measures
fs2a <- ggplot(
  datp |> filter(Variable %in% c(
    "Age", "Number of sexual partners",
    "Subjective sexual\nattractiveness", "Subjective sexual arousal"
  )),
  aes(Value, fill = Gender, colour = Gender)
) +
  geom_density(alpha = 0.3) + # Density plot with transparency
  geom_vline(
    data = datp |>
      filter(Variable %in% c(
        "Age", "Number of sexual partners",
        "Subjective sexual\nattractiveness", "Subjective sexual arousal"
      )) |>
      group_by(Variable, Gender) |>
```

```
      summarise(mean = mean(Value, na.rm = TRUE)), # Compute mean per group
    size = 1, aes(xintercept = mean, color = Gender, linetype = Gender)
  ) +
  scale_color_manual(values = color.Gender) + # Custom gender colors
  scale_fill_manual(values = color.Gender) +
  facet_wrap(~Variable, scales = "free", ncol = 4) + # Facet by variable
  labs(y = "Density", x = NULL) +
  theme_tq() # Apply tidyquant theme

# Panel 2: Sexual Function and Satisfaction
fs2b <- ggplot(
  datp |> filter(Variable %in% c(
    "MGH-SFQ (total)", "MGSS sexual satisfaction\n(General)",
    "MGSS sexual satisfaction\n(Partner)"
  )),
  aes(Value, fill = Gender, colour = Gender)
) +
  geom_density(alpha = 0.3) +
  geom_vline(
    data = datp |>
      filter(Variable %in% c(
        "MGH-SFQ (total)", "MGSS sexual satisfaction\n(General)",
        "MGSS sexual satisfaction\n(Partner)"
      )) |>
      group_by(Variable, Gender) |>
      summarise(mean = mean(Value, na.rm = TRUE)),
    size = 1, aes(xintercept = mean, color = Gender, linetype = Gender)
  ) +
  scale_color_manual(values = color.Gender) +
  scale_fill_manual(values = color.Gender) +
  facet_wrap(~Variable, scales = "free", ncol = 3) +
  labs(y = "Density", x = NULL) +
  theme_tq()

# Panel 3: Sexual Desire Measures
fs2c <- ggplot(
  datp |> filter(Variable %in% c(
    "Solitary sexual desire", "Dyadic sexual desire\n(Attractive person)",
    "Dyadic sexual desire (Partner)"
  )),
  aes(Value, fill = Gender, colour = Gender)
) +
  geom_density(alpha = 0.3) +
  geom_vline(
    data = datp |>
      filter(Variable %in% c(
        "Solitary sexual desire", "Dyadic sexual desire\n(Attractive person)",
        "Dyadic sexual desire (Partner)"
      )) |>
      group_by(Variable, Gender) |>
      summarise(mean = mean(Value, na.rm = TRUE)),
    size = 1, aes(xintercept = mean, color = Gender, linetype = Gender)
  ) +
  scale_color_manual(values = color.Gender) +
  scale_fill_manual(values = color.Gender) +
  facet_wrap(~Variable, scales = "free", ncol = 3) +
```

```
  labs(y = "Density", x = NULL) +
  theme_tq()

# Combine the three panels into a single figure
ggarrange(fs2a, fs2b, fs2c,
  common.legend = TRUE, legend = "bottom", nrow = 3 # Common legend below, 3-row layout
)
```

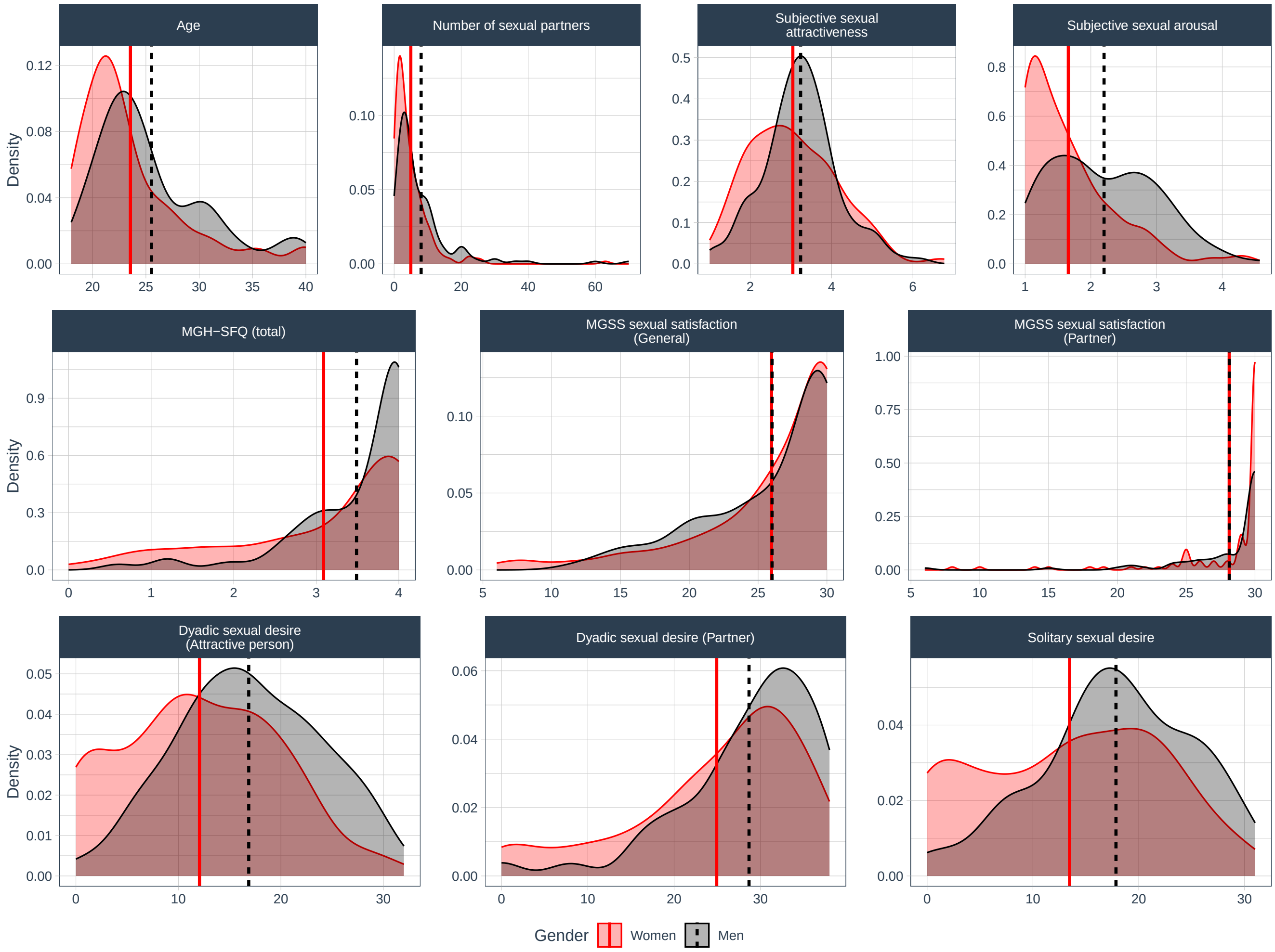

Figure S2. Distribution of measured variables by gender. Coloured vertical lines represent mean values by gender. Detailed descriptives are found in Table S1. Because for *Subjective sexual attractiveness* and *Subjective sexual arousal* there are are multiple within-subject observations, densities calculated from mean values per participant.

3.2 Correlations between measured variables

Correlation between numeric variables for women, men, and all participants combined, are reported in Table S2.

3.2.1 Table S2. Correlations between measured variables

Correlation matrix table.

```
# Compute correlations for women
dat.corr.W <- dat.desc |>
  ungroup() |>
  filter(Gender == "Women") |> # Select only women
  select(Age:`Dyadic sexual desire (Partner)`) |> # Select numeric variables
```

```
  corr.stars() |> # Compute correlation matrix with significance stars
  rownames_to_column(var = " ") # Move row names to a column

# Compute correlations for men
dat.corr.M <- dat.desc |>
  ungroup() |>
  filter(Gender == "Men") |> # Select only men
  select(Age:`Dyadic sexual desire (Partner)`) |> # Select numeric variables
  corr.stars() |>
  rownames_to_column(var = " ")

# Compute correlations for all participants combined
dat.corr.All <- dat.desc |>
  ungroup() |>
  select(Age:`Dyadic sexual desire (Partner)`) |>
  corr.stars() |>
  rownames_to_column(var = " ")

# Format and combine the correlation tables
bind_rows(dat.corr.W, dat.corr.M, dat.corr.All) |>
  kable(
    digits = 2, booktabs = TRUE, align = c("l", rep("c", 9)),
    linesep = "",
    caption = "Correlations between measured variables", escape = FALSE
  ) |>
  # Add grouped row labels for each participant group
  pack_rows("Women",
    start_row = 1, end_row = 10, bold = FALSE,
    background = "lightgray"
  ) |>
  pack_rows("Men",
    start_row = 11, end_row = 20, bold = FALSE,
    background = "lightgray"
  ) |>
  pack_rows("All participants",
    start_row = 21, end_row = 30, bold = FALSE,
    background = "lightgray"
  ) |>
  # Apply LaTeX styling
  kable_styling(latex_options = c("HOLD_position", "scale_down")) |>
  column_spec(2:10, width = "2.2cm") |> # Adjust column widths
  # Add footnote explaining correlation significance levels
  footnote(
    general = paste0(
      "Values represent Pearson correlation coefficients ($r$). ",
      "For significance, $^{\\\\dagger}p$ < 0.1, *$p$ < 0.05, ",
      "**$p$ < 0.01, ***$p$ < 0.001. ",
      "Significant correlations are in bold."
    ),
    threeparttable = TRUE, footnote_as_chunk = TRUE, escape = FALSE
  ) |>
  landscape() # Rotate table for better readability in LaTeX
```

Table S2. *Correlations between measured variables*

	Age	Number of sexual partners	MGH-SFQ (total)	MGSS sexual satisfaction (General)	MGSS sexual satisfaction (Partner)	Subjective sexual attractiveness	Subjective sexual arousal	Solitary sexual desire	Dyadic sexual desire (Attractive person)
**Women**									
Age									
Number of sexual partners	**0.24****								
MGH-SFQ (total)	-0.05	-0.07							
MGSS sexual satisfaction (General)	**-0.21***	0.02	**0.46*****						
MGSS sexual satisfaction (Partner)	-0.16†	-0.14	**0.32*****	**0.73*****					
Subjective sexual attractiveness	0.11	**0.18***	-0.04	**-0.22***	-0.18†				
Subjective sexual arousal	0.00	**0.17***	-0.13†	-0.18†	-0.16†	**0.54*****			
Solitary sexual desire	-0.14†	**0.28*****	0.05	-0.06	-0.18†	**0.31*****	**0.33*****		
Dyadic sexual desire (Attractive person)	0.06	**0.32*****	**-0.17***	-0.04	-0.17†	**0.34*****	**0.36*****	**0.44*****	
Dyadic sexual desire (Partner)	0.00	**0.21****	**0.43*****	**0.44*****	**0.27****	0.13†	0.04	**0.31*****	0.13†
**Men**									
Age									
Number of sexual partners	**0.23****								
MGH-SFQ (total)	0.04	0.02							
MGSS sexual satisfaction (General)	**-0.24***	-0.08	**0.36*****						
MGSS sexual satisfaction (Partner)	-0.13	-0.01	0.10	**0.63*****					
Subjective sexual attractiveness	0.10	-0.05	-0.08	-0.10	-0.02				
Subjective sexual arousal	**0.2***	0.07	0.05	-0.14	-0.09	**0.46*****			
Solitary sexual desire	-0.16†	0.00	0.09	0.10	0.17	**0.26****	0.11		
Dyadic sexual desire (Attractive person)	0.12	**0.29*****	0.03	-0.13	-0.08	**0.25****	**0.43*****	**0.25****	
Dyadic sexual desire (Partner)	0.11	0.07	**0.36*****	**0.55*****	**0.22***	0.14	**0.24****	**0.17***	**0.2***
**All participants**									
Age									
Number of sexual partners	**0.26*****								
MGH-SFQ (total)	0.02	0.01							
MGSS sexual satisfaction (General)	**-0.22****	-0.03	**0.42*****						
MGSS sexual satisfaction (Partner)	**-0.14***	-0.07	**0.24*****	**0.69*****					
Subjective sexual attractiveness	**0.12***	0.08	-0.03	**-0.18***	-0.12				
Subjective sexual arousal	**0.15****	**0.17****	0.01	**-0.15***	-0.12†	**0.5*****			
Solitary sexual desire	-0.09	**0.17****	0.11†	0.00	-0.05	**0.31*****	**0.3*****		
Dyadic sexual desire (Attractive person)	**0.14***	**0.33*****	-0.04	-0.07	-0.12†	**0.32*****	**0.45*****	**0.42*****	
Dyadic sexual desire (Partner)	0.08	**0.16****	**0.43*****	**0.46*****	**0.25*****	**0.15****	**0.18****	**0.3*****	**0.21*****

Note: Values represent Pearson correlation coefficients (r). For significance, $^{\dagger}p < 0.1$, $^{*}p < 0.05$, $^{**}p < 0.01$, $^{***}p < 0.001$. Significant correlations are in bold.

3.3 Internal consistency

Six variables were calculated from multiple items (1. MGH-SFQ, 2. Dyadic sexual desire (Partner), 3. Solitary sexual desire, 4. Dyadic sexual desire (Attractive person), 5. MGSS sexual satisfaction (General) and 6. MGSS sexual satisfaction (Partner)).

Data by item, for each participant, is included in the following data base, loaded as `dat.reli`:

```
# Load dataset containing item-level data for internal consistency analysis
dat.reli <- read_excel("data/BD_ConsistenciaInterna.xlsx") |>
  # Recode gender variable: 1 = Men, 2 = Women
  mutate(Sex = recode_factor(Sex, "2" = "Women", "1" = "Men")) |>
  rename(Gender = Sex) |> # Rename column for consistency with other datasets
  filter(Participante != 122) # Remove participant 122 from the dataset
```

Participant 122 was excluded because they did not respond the psychological scales.

To measure the internal consistency of these tests, we used standardized Cronbach's alpha (α or Tau-equivalent reliability: ρ_T) coefficients, using the function `cronbach.alpha` from the package `ltm` (Rizopoulos, 2006).

Importantly, given that for MGH-SFQ one item was answered only by men, the internal consistency of this variable was measured independently for each gender.

```
# Compute standardized Cronbach's alpha (Tau-equivalent reliability) for each measure

# MGH-SFQ for men (selects items 3 to 7, excludes missing values)
MGH.m <- dat.reli |>
  filter(Gender == "Men") |>
  select(3:7) |>
  drop_na() |>
  cronbach.alpha(CI = TRUE, standardized = TRUE)

# MGH-SFQ for women (excludes item 6, which was answered only by men)
MGH.w <- dat.reli |>
  filter(Gender == "Women") |>
  select(3:5, 7) |>
  drop_na() |>
  cronbach.alpha(CI = TRUE, standardized = TRUE)

# Dyadic sexual desire (Partner) - items 9 to 13
DSD.p <- dat.reli |>
  select(9:13) |>
  drop_na() |>
  cronbach.alpha(CI = TRUE, standardized = TRUE)

# Solitary sexual desire - items 15 to 18
SSD.p <- dat.reli |>
  select(15:18) |>
  drop_na() |>
  cronbach.alpha(CI = TRUE, standardized = TRUE)

# Dyadic sexual desire (Attractive person) - items 20 to 23
DSD.a <- dat.reli |>
  select(20:23) |>
  drop_na() |>
  cronbach.alpha(CI = TRUE, standardized = TRUE)

# MGSS sexual satisfaction (General) - items 26 to 30
MGSS.g <- dat.reli |>
  select(26:30) |>
```

```
  drop_na() |>
  cronbach.alpha(CI = TRUE, standardized = TRUE)

# MGSS sexual satisfaction (Partner) - items 32 to 36
MGSS.p <- dat.reli |>
  select(32:36) |>
  drop_na() |>
  cronbach.alpha(CI = TRUE, standardized = TRUE)
```

3.3.1 Table S3. Internal consistency of construct variables

Table of Cronbach's α for construct variables.

```
# Create a table of Cronbach's alpha values for internal consistency analysis
tibble(
  Variable = c(
    "MGH-SFQ", "MGH-SFQ",
    "MGSS sexual satisfaction (General)",
    "MGSS sexual satisfaction (Partner)",
    "Dyadic TSD Partner", "Solitary TSD",
    "Dyadic TSD Attractive Person"
  ),
  Gender = c("Men", "Women", rep(" ", 5)), # Report gender only for MGH-SFQ
  p = c(
    MGH.m$p, MGH.w$p, MGSS.g$p, MGSS.p$p,
    DSD.p$p, SSD.p$p, DSD.a$p
  ),
  n = c(
    MGH.m$n, MGH.w$n, MGSS.g$n, MGSS.p$n,
    DSD.p$n, SSD.p$n, DSD.a$n
  ),
  alpha = c(
    MGH.m$alpha, MGH.w$alpha, MGSS.g$alpha, MGSS.p$alpha,
    DSD.p$alpha, SSD.p$alpha, DSD.a$alpha
  ),
  ci2.5 = c(
    MGH.m$ci[1], MGH.w$ci[1], MGSS.g$ci[1], MGSS.p$ci[1],
    DSD.p$ci[1], SSD.p$ci[1], DSD.a$ci[1]
  ),
  ci97.5 = c(
    MGH.m$ci[2], MGH.w$ci[2], MGSS.g$ci[2], MGSS.p$ci[2],
    DSD.p$ci[2], SSD.p$ci[2], DSD.a$ci[2]
  )
) |>
  kable(
    digits = 2, booktabs = TRUE, align = c("l", "l", rep("c", 5)),
    linesep = "",
    caption = "Internal consistency of measured variables", escape = FALSE,
    col.names = c(
      "Variable", "Gender", "Items", "$n$", "$\\alpha$", "$2.5\\% CI$", "$97.5\\% CI$"
    )
  ) |>
  # Merge repeated values in the "Variable" column for better readability
  collapse_rows(columns = 1, valign = "middle") |>
  # Apply LaTeX styling for table positioning
  kable_styling(latex_options = "HOLD_position") |>
  # Add footnote explaining confidence intervals and reporting choices
```

```
  footnote(
    general = "95\\\\% confidence intervals were calculated with 1,000 bootstrap samples.
           Standardized Cronbach's alpha ($\\\\alpha$) coefficients were computed.
           MGH-SFQ is reported by gender, because one item was answered only by men.",
    threeparttable = TRUE, footnote_as_chunk = TRUE, escape = FALSE
  )
```

Table S3. *Internal consistency of measured variables*

Variable	Gender	Items	n	α	2.5%CI	97.5%CI
MGH-SFQ	Men	5	139	0.82	0.71	0.88
	Women	4	181	0.86	0.82	0.90
MGSS sexual satisfaction (General)		5	188	0.92	0.88	0.94
MGSS sexual satisfaction (Partner)		5	187	0.91	0.85	0.95
Dyadic TSD Partner		5	309	0.90	0.88	0.92
Solitary TSD		4	314	0.91	0.89	0.93
Dyadic TSD Attractive Person		4	320	0.89	0.87	0.91

Note: 95% confidence intervals were calculated with 1,000 bootstrap samples. Standardized Cronbach's alpha (α) coefficients were computed. MGH-SFQ is reported by gender, because one item was answered only by men.

3.4 Controlling for Relationship Duration and MGSS Sexual Satisfaction (Partner) in Sexual Desire Dimensions

To **ensure that the three sexual desire dimensions were not influenced by Relationship Duration or MGSS sexual satisfaction (Partner)**, we applied a three-step adjustment process:

1. **Estimating the effects:**
   - We performed separate **linear regressions** where each sexual desire dimension was predicted by **Relationship Duration** and **MGSS sexual satisfaction (Partner)**.
   - This allowed us to quantify how much these external factors influence each dimension.
2. **Evaluating statistical significance:**
   - We conducted **Type III ANOVA** to determine which predictors had a significant effect on each sexual desire dimension.
   - Only **MGSS sexual satisfaction (Partner) significantly predicted Dyadic Sexual Desire (Partner)**.
3. **Removing the effects:**
   - We adjusted **only Dyadic Sexual Desire (Partner)** by extracting the **residuals** from the regression model.
   - These residuals represent the **variation independent of MGSS sexual satisfaction (Partner)** and were then standardized for comparability.

Additionally, **MGSS sexual satisfaction (Partner) was mean-centered** before analysis.

Step 1: Estimating the Effects of Relationship Duration & Partner Satisfaction

```
# Select participants in stable relationships and ensure required variables are available
dat_ctl <- dat |>
  group_by(Participant) |>
  slice_head() |> # Retain only the first occurrence per participant
  filter(Relationship == "Stable") |> # Include only participants in stable relationships
  ungroup()
```

```
# Fit linear models predicting each sexual desire dimension

# Model predicting Solitary TSD
ctl_SSD <- lm(
  `Solitary sexual desire` ~ `Relationship duration` + `MGSS sexual satisfaction (Partner)`,
  data = dat_ctl
)

# Model predicting Dyadic Sexual Desire (Partner)
ctl_PD <- lm(
  `Dyadic sexual desire (Partner)` ~ `Relationship duration` +
    `MGSS sexual satisfaction (Partner)`,
  data = dat_ctl
)

# Model predicting Dyadic Sexual Desire (Attractive Person)
ctl_APD <- lm(
  `Dyadic sexual desire (Attractive person)` ~ `Relationship duration` +
    `MGSS sexual satisfaction (Partner)`,
  data = dat_ctl
)
```

Step 2: Displaying ANOVA Results for Each Model

The table below presents Type III ANOVA results for each model. Significant effects indicate that Relationship Duration or Partner Satisfaction meaningfully predict the corresponding sexual desire dimension.

```
# Compute ANOVA results and effect sizes for all models
anova_results <- bind_cols(
  bind_cols(
    anova_summary(Anova(ctl_SSD, type = 3)), # Type III ANOVA for Solitary TSD
    epsilon_squared(ctl_SSD) # Compute partial epsilon squared
  ) |>
    unite(col = "df", DFn:DFd, sep = ", "), # Combine numerator and denominator df
  bind_cols(
    anova_summary(Anova(ctl_PD, type = 3)), # Type III ANOVA for Dyadic Sexual Desire (Partner)
    epsilon_squared(ctl_PD)
  ) |>
    unite(col = "df", DFn:DFd, sep = ", "),
  bind_cols(
    anova_summary(Anova(ctl_APD, type = 3)), # Type III ANOVA for Dyadic Sexual Desire (Attractive)
    epsilon_squared(ctl_APD)
  ) |>
    unite(col = "df", DFn:DFd, sep = ", ")
) |>
  # Remove unnecessary columns related to Sum of Squares and CI
  select(-starts_with(c("p<.05", "ges...", "Parameter...", "CI"))) |>
  # Format p-values for LaTeX
  mutate(across(starts_with("p..."), pval.lev)) |>
  rename(Effect = Effect...1) |> # Rename effect column
  # Remove redundant effect columns and clean effect names
  select(-starts_with("Effect...")) |>
  mutate_at("Effect", str_replace_all, "`", "")

# Create a formatted ANOVA results table
anova_results |>
  kable(
```

```
  booktabs = TRUE, align = c("l", rep("c", 9)), digits = 3, escape = FALSE,
  caption = "Effects of relationship duration and MGSS sexual satisfaction (Partner) in
               sexual desire dimensions",
  col.names = c("Effect", rep(c("$df$", "$F$", "$p$", "$\\epsilon^2_p$"), times = 3))
) |>
# Apply LaTeX styling for table positioning
kable_styling(latex_options = c("HOLD_position", "scale_down")) |>
# Add section headers for the three models
add_header_above(c(
  " " = 1,
  "Solitary sexual desire" = 4,
  "Dyadic sexual desire\n(Partner)" = 4,
  "Dyadic sexual desire\n(Attractive person)" = 4
)) |>
# Add footnote explaining effect sizes and significance
footnote(
  general = "As effect size, we report partial epsilon squared
               ($\\\\epsilon^2_p$), which provides a less biased
               estimate than $\\\\eta^2$ (see
               \\\\cite{albersWhenPowerAnalyses2018}).
               Significant effects are in bold.",
  threeparttable = TRUE, footnote_as_chunk = TRUE, escape = FALSE
)
```

Table S4. *Effects of relationship duration and MGSS sexual satisfaction (Partner) in sexual desire dimensions*

	Solitary sexual desire				Dyadic sexual desire (Partner)				Dyadic sexual desire (Attractive person)			
Effect	*df*	*F*	*p*	ϵ^2_p	*df*	*F*	*p*	ϵ^2_p	*df*	*F*	*p*	ϵ^2_p
Relationship duration	3, 165	0.482	0.70	0	3, 165	2.081	0.1	0.041	3, 165	0.095	0.96	0
MGSS sexual satisfaction (Partner)	1, 165	0.029	0.86	0	1, 165	8.875	**0.003**	0.045	1, 165	0.884	0.35	0

Note: As effect size, we report partial epsilon squared (ϵ^2_p), which provides a less biased estimate than η^2 (see Albers and Lakens, 2018). Significant effects are in bold.

Step 3: Controlling Scores Based on ANOVA Results

From the ANOVA results, only the effect of MGSS sexual satisfaction (Partner) on Dyadic sexual desire (Partner) was significant. Thus, only Dyadic Sexual Desire (Partner) scores were adjusted, while the other dimensions remained unchanged.

```
# Prepare dataset with relevant variables and remove missing values
dat_tl_PD_fin <- dat_ctl |>
  select(
    Participant, `Dyadic sexual desire (Partner)`, `MGSS sexual satisfaction (Partner)`
  ) |>
  drop_na() # Remove rows with missing values

# Fit a linear model predicting Dyadic Sexual Desire (Partner) using partner satisfaction
ctl_PD_fin <- lm(
  `Dyadic sexual desire (Partner)` ~ `MGSS sexual satisfaction (Partner)`,
  data = dat_tl_PD_fin
)

# Adjust Dyadic Sexual Desire (Partner) scores using residuals from the regression model
dat_ctl <- dat_tl_PD_fin |>
  mutate(
```

```
    `Dyadic sexual desire (Partner)` =
      mean(`Dyadic sexual desire (Partner)`) + resid(ctl_PD_fin) # Centered residuals
  )

# Update the original dataset with adjusted scores for Dyadic Sexual Desire (Partner)
dat <- dat |>
  mutate(`Dyadic sexual desire (Partner)` = as.numeric(`Dyadic sexual desire (Partner)`)) |>
  rows_update(
    dat_ctl |> select(-`MGSS sexual satisfaction (Partner)`), # Remove predictor column
    by = "Participant", unmatched = "ignore" # Match by participant ID
  )
```

---

4 Hypothesis tests

4.1 Hypothesis 1: All dimensions of trait sexual desire (TSD) will be higher in men than in women, and the differences will be stronger or weaker according to relationship status

We tested whether relationship type and gender interact as predictors of sexual desire (H1a: Solitary TSD; H1b: Dyadic TSD toward an attractive person; H1c: Dyadic TSD toward a partner). To examine this hypothesis, we modeled the effects of relationship type and gender on each of the three TSD scores.

However, models using the original TSD scores did not meet the assumption of normally distributed residuals. To address this, we applied an ordered normalization transformation to each TSD variable. We then fitted and compared models predicting both the original (as a proportion, to make scores comparable) and transformed (normalized) TSD dimensions. In all three cases, models using the normalized variables provided a better fit, so all inferences are based on these models.

4.1.1 Data

A data frame was created with one row per participant, where sexual desire variables were normalized as proportions. An ordered quantile normalization transformation (Peterson & Cavanaugh, 2020) was then applied using the `orderNorm` function from the `bestNormalize` package (Peterson, 2021), and the transformed values were added as new variables.

```
# Process the dataset and create transformed variables
dat_m1 <- dat |>
  group_by(Participant) |>
  slice_head() |> # Retain only the first observation per participant
  ungroup() |>
  # Create proportion variables to normalize each sexual desire measure
  mutate(
    "Solitary sexual desire (proportion)" = `Solitary sexual desire` / 31,
    "Dyadic TSD Attractive Person (proportion)" =
      `Dyadic sexual desire (Attractive person)` / 32,
    "Dyadic TSD Partner (proportion)" = `Dyadic sexual desire (Partner)` / 38
  )

# Apply ordered normalization transformations to the proportion variables
trs_SSD <- orderNorm(dat_m1$`Solitary sexual desire (proportion)`)
trs_DSDat <- orderNorm(dat_m1$`Dyadic TSD Attractive Person (proportion)`)
trs_DSDpt <- orderNorm(dat_m1$`Dyadic TSD Partner (proportion)`)

# Add the transformed variables back into the dataset
dat_m1 <- dat_m1 |>
```

```
  mutate(
    "Solitary TSD (normalized)" = predict(trs_SSD), # Transformed Solitary TSD
    "Dyadic TSD Attractive Person (normalized)" = predict(trs_DSDat),
    "Dyadic TSD Partner (normalized)" = predict(trs_DSDpt)
  )
```

4.1.2 Hypothesis 1a: Solitary TSD

4.1.2.1 Model the effects of relationship type and gender on Solitary TSD We fitted models with both the original (proportion; `m1a_prop`) and transformed (normalized; `m1a_norm`) TSD scores, and performed posterior predictive checks (PPCs). As shown elsewhere (e.g., Gabry et al., 2019), if simulated data from one model are more similar to the observed outcome, that model is likely to be preferred.

```
# Fit a linear model using the original proportion-based Solitary TSD scores
m1a_prop <- lm(
  `Solitary sexual desire (proportion)` ~ Gender * Relationship,
  data = dat_m1
)

# Fit a linear model using the normalized Solitary TSD scores
m1a_norm <- lm(
  `Solitary TSD (normalized)` ~ Gender * Relationship,
  data = dat_m1
)
```

4.1.2.1.1 Figure S3: Posterior predictive checks (PPCs) for Hypothesis 1a. PPCs were performed using the `check_model` function from the `performance` package (Lüdecke et al., 2021), and reported in Fig. S3. Simulated data from the normalized Solitary TSD model (Fig. S3b) are more similar to the observed outcome, so this model is preferred.

```
# Perform posterior predictive checks (PPC) and arrange them into a single figure
ppc_m1a <- ggarrange(
  # PPC plot for the original (proportion) Solitary TSD model
  plot(
    check_model(m1a_prop, panel = FALSE, check = "pp_check")$PP_CHECK,
    colors = c("red", "grey30") # Red for observed data, grey for simulated data
  ) +
    labs(title = NULL, subtitle = NULL) +
    theme_tq() + # Apply tidyquant theme
    facet_wrap(~1, labeller = as_labeller(c(
      "1" = "Original (proportion) Solitary TSD"
    ))),
  # PPC plot for the transformed (normalized) Solitary TSD model
  plot(
    check_model(m1a_norm, panel = FALSE, check = "pp_check")$PP_CHECK,
    colors = c("red", "grey30")
  ) +
    labs(title = NULL, subtitle = NULL) +
    theme_tq() +
    facet_wrap(~1, labeller = as_labeller(c(
      "1" = "Transformed (normalized) Solitary TSD"
    ))),
  labels = "auto", # Automatically label subplots (a, b)
  common.legend = TRUE, legend = "bottom" # Use a common legend at the bottom
)

# Display the final PPC figure
```

```
ppc_m1a
```

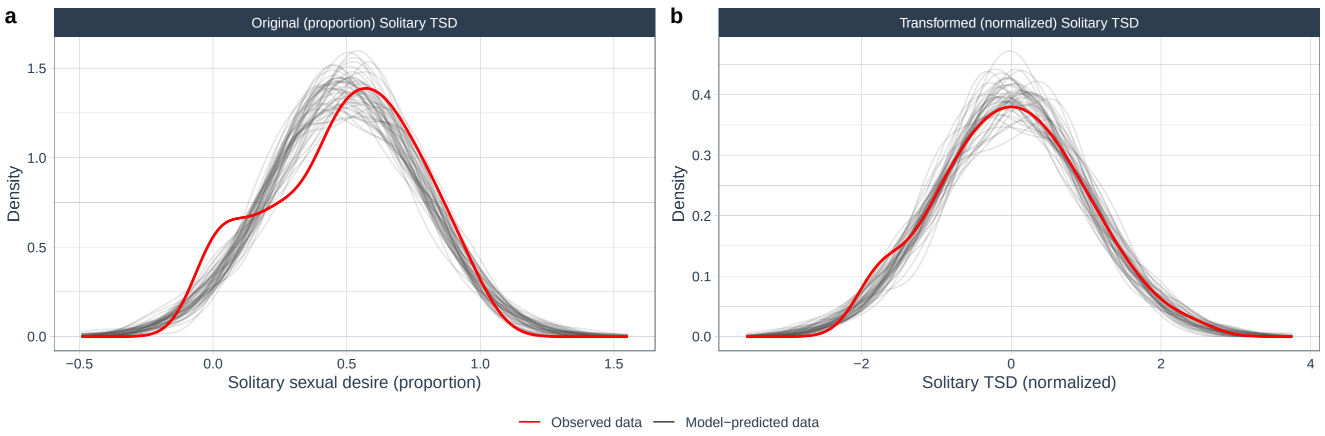

Figure S3. Posterior predictive check. **(a)** Original (proportion) Solitary TSD; **(b)** Transformed (normalized) Solitary TSD. In both panels, red lines represent the observed data, and thin black lines represent 50 iterations of simulated data from each model.

4.1.2.2 Table S5. ANOVA-type table for the interaction between `Relationship type`, and `Gender`

This tables summarizes the results of the model.

```
# Generate an ANOVA table summarizing the effects of Relationship Type and Gender on Solitary TSD
anova.sig.lm(
  model = m1a_norm,
  custom_caption = "Effects of relationship type and gender on Solitary TSD"
)
```

Table S5. *Effects of relationship type and gender on Solitary TSD*

Effect	df	F	p	ϵ_p^2
Gender	1, 319	22.42	$<$ **0.0001**	0.06
Relationship	1, 319	14.07	$<$ **0.001**	0.03
Gender × Relationship	1, 319	4.23	**0.04**	0.01

Note:
Sexual desire was transformed using an ordered quantile normalization (Peterson and Cavanaugh, 2020). Results are Type III ANOVA. $R^2 = 0.103$, $R^2_{adjusted} = 0.095$. Gender = participant's gender (women, men); Relationship = relationship type (stable, single). As effect size, we report partial epsilon squared (ϵ_p^2), a less biased estimate than η^2 (see Albers and Lakens, 2018). Significant effects are in bold.

4.1.2.3 *Post-hoc* comparisons

Because the main effects of gender, relationship type, and their interaction are significant, we explored these effects using estimated marginal means.

4.1.2.3.1 Table S6. Estimated marginal means and contrasts between participants' gender.

Table of estimated marginal means and contrasts between genders. All estimated marginal means and contrasts were calculated using the `emmeans` function from the `emmeans` package (Lenth, 2023).

```
# Compute estimated marginal means (EMMs) for Gender from the model
emms.m1a1 <- emmeans(m1a_norm, ~Gender)

# Convert EMM results to a tibble for easier manipulation
```

```
emms.m1a1.tab <- tibble(data.frame(emms.m1a1))

# Compute post-hoc contrasts and format p-values
t.m1a1 <- contr.stars(emms.m1a1) |>
  mutate(p.value = pval.lev(p.value)) # Format p-values for LaTeX

# Merge EMM and contrast results, clean column names, and format output
merge(emms.m1a1.tab, t.m1a1, by = 0, all = TRUE) |>
  select(-c(1, 15)) |> # Remove unnecessary columns
  unite(Contrast, group1, group2, sep = " - ") |> # Create contrast labels
  mutate_at("Contrast", str_replace_all, "NA - NA", " ") |> # Replace missing contrasts
  # Create formatted table
  kable(
    digits = 2, booktabs = TRUE, align = c("l", rep("c", 5), "l", rep("c", 5)),
    linesep = "",
    caption = "Estimated marginal means and contrasts between participants' gender",
    escape = FALSE,
    col.names = c(
      "Gender", "EMM", "$SE$", "$df$", "$2.5\\% CI$", "$97.5\\% CI$",
      "Contrast", "Difference", "$SE$", "$df$", "$t$", "$p$"
    )
  ) |>
  # Add a header separating EMMs and contrasts
  add_header_above(c(" " = 6, "Contrasts" = 6)) |>
  # Apply LaTeX styling for table positioning
  kable_styling(latex_options = c("HOLD_position", "scale_down")) |>
  # Add footnote explaining significance formatting
  footnote(
    general = "Significant effects are in bold.", threeparttable = TRUE,
    footnote_as_chunk = TRUE, escape = FALSE
  )
```

Table S6. *Estimated marginal means and contrasts between participants' gender*

						Contrasts					
Gender	EMM	*SE*	*df*	2.5%*CI*	97.5%*CI*	Contrast	Difference	*SE*	*df*	*t*	*p*
Women	-0.17	0.07	319	-0.30	-0.03	Women - Men	-0.46	0.1	319	-4.36	$<$ **0.0001**
Men	0.29	0.08	319	0.13	0.44						

Note: Significant effects are in bold.

4.1.2.3.2 Table S7. Estimated marginal means and contrasts between relationship status.

Table of estimated marginal means and contrasts between relationship status. All estimated marginal means and contrasts were calculated using the `emmeans` function from the `emmeans` package (Lenth, 2023).

```
# Compute estimated marginal means (EMMs) for Relationship Status from the model
emms.m1a2 <- emmeans(m1a_norm, ~Relationship)

# Convert EMM results to a tibble for easier manipulation
emms.m1a2.tab <- tibble(data.frame(emms.m1a2))

# Compute post-hoc contrasts and format p-values
t.m1a2 <- contr.stars(emms.m1a2) |>
  mutate(p.value = pval.lev(p.value)) # Format p-values for LaTeX

# Merge EMM and contrast results, clean column names, and format output
```

```
merge(emms.m1a2.tab, t.m1a2, by = 0, all = TRUE) |>
  select(-c(1, 15)) |> # Remove unnecessary columns
  unite(Contrast, group1, group2, sep = " - ") |> # Create contrast labels
  mutate_at("Contrast", str_replace_all, "NA - NA", " ") |> # Replace missing contrasts
  # Create formatted table
  kable(
    digits = 2, booktabs = TRUE, align = c("l", rep("c", 5), "l", rep("c", 5)),
    linesep = "",
    caption = "Estimated marginal means and contrasts between relationship status",
    escape = FALSE,
    col.names = c(
      "Relationship type", "EMM", "$SE$", "$df$", "$2.5\\% CI$", "$97.5\\% CI$",
      "Contrast", "Difference", "$SE$", "$df$", "$t$", "$p$"
    )
  ) |>
  # Add a header separating EMMs and contrasts
  add_header_above(c(" " = 6, "Contrasts" = 6)) |>
  # Apply LaTeX styling for table positioning
  kable_styling(latex_options = c("HOLD_position", "scale_down")) |>
  # Add footnote explaining significance formatting
  footnote(
    general = "Significant effects are in bold.", threeparttable = TRUE,
    footnote_as_chunk = TRUE, escape = FALSE
  )
```

Table S7. *Estimated marginal means and contrasts between relationship status*

						Contrasts					
Relationship type	EMM	*SE*	*df*	2.5%*CI*	97.5%*CI*	Contrast	Difference	*SE*	*df*	*t*	*p*
Stable	-0.09	0.07	319	-0.23	0.05	Stable - Single	-0.3	0.1	319	-2.89	**0.0041**
Single	0.21	0.08	319	0.06	0.36						

Note: Significant effects are in bold.

4.1.2.3.3 Table S8. Estimated marginal means and contrasts between gender by relationship status.

Table of estimated marginal means and contrasts between gender by relationship status. All estimated marginal means and contrasts were calculated using the `emmeans` function from the `emmeans` package (Lenth, 2023).

```
# Compute estimated marginal means (EMMs) for Gender within each Relationship Status
emms.m1a3 <- emmeans(m1a_norm, ~ Gender | Relationship)

# Convert EMM results to a tibble for easier manipulation
emms.m1a3.tab <- tibble(data.frame(emms.m1a3))

# Compute post-hoc contrasts and format p-values
t.m1a3 <- contr.stars(emms.m1a3) |>
  mutate(p.value = pval.lev(p.value)) # Format p-values for LaTeX

# Insert NA rows to maintain table structure for grouped relationship status display
t.m1a3.f <- t.m1a3 |>
  insertRows(2, new = NA) |> # Insert empty row after first contrast
  insertRows(4, new = NA) # Insert empty row after second contrast

# Merge EMM and contrast results, clean column names, and format output
merge(emms.m1a3.tab, t.m1a3.f, by = 0, all = TRUE) |>
  select(-c(1, 3, 11, 17)) |> # Remove unnecessary columns
  drop_na(Gender) |> # Ensure Gender column is complete
```

```
  unite(Contrast, group1, group2, sep = " - ") |> # Create contrast labels
  mutate_at("Contrast", str_replace_all, "NA - NA", "") |> # Replace missing contrasts
  # Create formatted table
  kable(
    digits = 2, booktabs = TRUE, align = c("l", "l", rep("c", 5), "l", rep("c", 5)),
    linesep = "",
    caption = "Estimated marginal means and contrasts between gender by relationship status",
    escape = FALSE,
    col.names = c(
      "Gender", "EMM", "$SE$", "$df$", "$2.5\\% CI$", "$97.5\\% CI$",
      "Contrast", "Difference", "$SE$", "$df$", "$t$", "$p$"
    )
  ) |>
  # Add grouped row labels for Relationship Status
  pack_rows("Relationship status: Stable",
    start_row = 1, end_row = 2,
    bold = FALSE, background = "lightgray"
  ) |>
  pack_rows("Relationship status: Single",
    start_row = 3, end_row = 4,
    bold = FALSE, background = "lightgray"
  ) |>
  # Add a header separating EMMs and contrasts
  add_header_above(c(" " = 6, "Contrasts" = 6)) |>
  # Apply LaTeX styling for table positioning
  kable_styling(latex_options = c("HOLD_position", "scale_down")) |>
  # Add footnote explaining significance formatting
  footnote(
    general = "Significant effects are in bold.", threeparttable = TRUE,
    footnote_as_chunk = TRUE, escape = FALSE
  )
```

Table S8. *Estimated marginal means and contrasts between gender by relationship status*

						Contrasts					
Gender	EMM	*SE*	*df*	2.5%*CI*	97.5%*CI*	Contrast	Difference	*SE*	*df*	*t*	*p*
Relationship status: Stable											
Women	-0.43	0.09	319	-0.61	-0.25	Women - Men	-0.67	0.14	319	-4.74	**$<$ 0.0001**
Men	0.24	0.11	319	0.03	0.46						
Relationship status: Single											
Women	0.09	0.10	319	-0.11	0.30	Women - Men	-0.24	0.15	319	-1.57	0.12
Men	0.33	0.11	319	0.11	0.55						

Note: Significant effects are in bold.

4.1.2.4 Figure S4. Effects of gender and relationship type on Solitary TSD

This figure summarizes the results of hypothesis 1a.

```
# Plot (a): Main effect of Gender on Solitary TSD
h1a1 <- ggplot(dat_m1, aes(
  x = Gender, y = `Solitary TSD (normalized)`, color = Gender
)) +
  scale_color_manual(values = color.Gender) +
  scale_fill_manual(values = color.Gender) +
  geom_linerange(
    data = emms.m1a1.tab |> rename("Solitary TSD (normalized)" = emmean),
```

```
    mapping = aes(ymin = lower.CL, ymax = upper.CL)
  ) +
  geom_point(
    data = emms.m1a1.tab |> rename("Solitary TSD (normalized)" = emmean),
    position = position_dodge(0.1), size = 3
  ) +
  stat_pvalue_manual(t.m1a1, label = "p.signif", y.position = 0.55, tip.length = 0) +
  guides(color = "none") +
  theme_tq()

# Plot (b): Main effect of Relationship on Solitary TSD
h1a2 <- ggplot(dat_m1, aes(
  x = Relationship, y = `Solitary TSD (normalized)`, color = Relationship
)) +
  scale_color_manual(values = color.Relationship) +
  scale_fill_manual(values = color.Relationship) +
  geom_linerange(
    data = emms.m1a2.tab |> rename("Solitary TSD (normalized)" = emmean),
    mapping = aes(ymin = lower.CL, ymax = upper.CL)
  ) +
  geom_point(
    data = emms.m1a2.tab |> rename("Solitary TSD (normalized)" = emmean),
    position = position_dodge(0.1), size = 3
  ) +
  stat_pvalue_manual(t.m1a2, label = "p.signif", y.position = 0.45, tip.length = 0) +
  guides(color = "none") +
  theme_tq()

# Plot (c): Gender × Relationship Interaction on Solitary TSD
h1a3 <- ggplot(dat_m1, aes(
  x = Gender, y = `Solitary TSD (normalized)`, color = Gender
)) +
  scale_color_manual(values = color.Gender) +
  scale_fill_manual(values = color.Gender) +
  facet_wrap(~Relationship) +
  geom_linerange(
    data = emms.m1a3.tab |> rename("Solitary TSD (normalized)" = emmean),
    mapping = aes(ymin = lower.CL, ymax = upper.CL)
  ) +
  geom_point(
    data = emms.m1a3.tab |> rename("Solitary TSD (normalized)" = emmean),
    position = position_dodge(0.1), size = 3
  ) +
  stat_pvalue_manual(t.m1a3, label = "p.signif", y.position = 0.7, tip.length = 0) +
  guides(color = "none") +
  theme_tq()

# Combine the three plots into a single figure
p1a <- ggarrange(h1a1, h1a2 + labs(y = NULL), h1a3 + labs(y = NULL),
  ncol = 3, labels = "auto", widths = c(1, 1, 1.5) # Adjust widths for better alignment
)

# Display the final figure
p1a
```

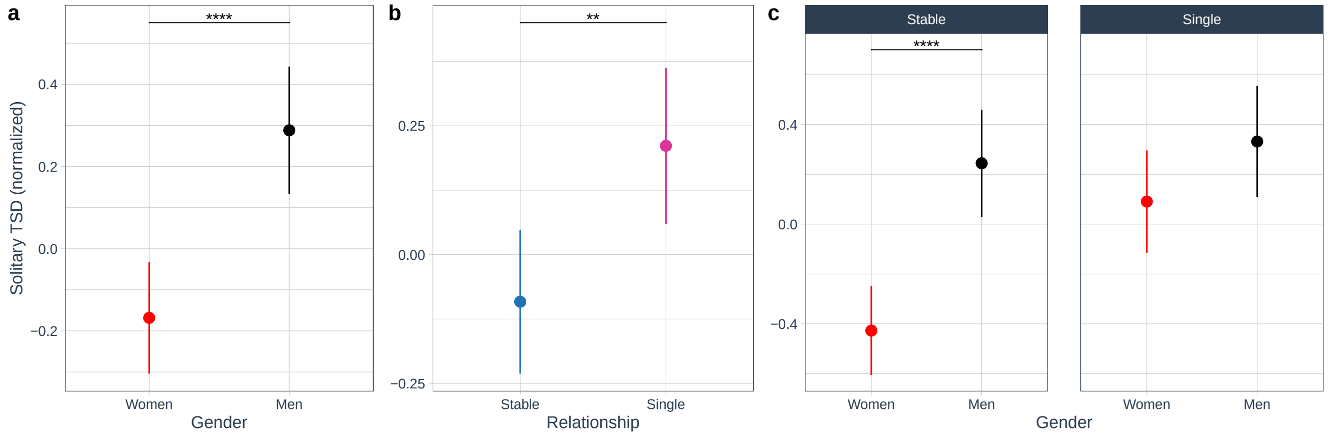

Figure S4. Effects of gender and relationship type on Solitary TSD. Solitary sexual desire was transformed using ordered quantile normalization (Peterson & Cavanaugh, 2020). **(a)** Simple comparison between sexual desire by gender (for detailed results, see Table S6); **(b)** Simple comparison between relationship status levels (for detailed results, see Table S7); **(c)** Interaction between relationship type and relationship status (see Table S5; for detailed results, see Table S8). Dots and bars represent estimated marginal means and 95% CI. In all cases, significant effects are represented with lines and stars: *$p < 0.05$, **$p < 0.01$, ***$p < 0.001$, ****$p < 0.0001$.

4.1.3 Hypothesis 1b: Dyadic TSD (Attractive person)

4.1.3.1 Model the effects of relationship type and gender on Dyadic TSD: Attractive person We fitted models with both the original (proportion; `m1b_prop`) and transformed (normalized; `m1b_norm`) TSD scores, and performed posterior predictive checks (PPCs). As shown elsewhere (e.g., Gabry et al., 2019), if simulated data from one model are more similar to the observed outcome, that model is likely to be preferred.

```
# Set contrast options for sum coding (useful for ANOVA-like interpretation)
options(contrasts = c("contr.sum", "contr.poly"))

# Fit a linear model using the original proportion-based Dyadic TSD (Attractive Person) scores
m1b_prop <- lm(
  `Dyadic TSD Attractive Person (proportion)` ~ Gender * Relationship,
  data = dat_m1
)

# Fit a linear model using the normalized Dyadic TSD (Attractive Person) scores
m1b_norm <- lm(
  `Dyadic TSD Attractive Person (normalized)` ~ Gender * Relationship,
  data = dat_m1
)
```

4.1.3.1.1 Figure S5: Posterior predictive checks (PPCs) for Hypothesis 1b. PPCs were performed using the `check_model` function from the `performance` package (Lüdecke et al., 2021), and reported in Fig. S5. Simulated data from the normalized Solitary TSD model (Fig. S5b) are more similar to the observed outcome, so this model is preferred.

```
# Perform posterior predictive checks (PPC) and arrange them into a single figure
ppc_m1b <- ggarrange(
  # PPC plot for the original (proportion) Dyadic TSD: Attractive Person model
  plot(
    check_model(m1b_prop, panel = FALSE, check = "pp_check")$PP_CHECK,
    colors = c("red", "grey30") # Red for observed data, grey for simulated data
  ) +
    labs(title = NULL, subtitle = NULL) +
    theme_tq() +
```

```
    facet_wrap(~1, labeller = as_labeller(c(
      "1" = "Original (proportion) Dyadic TSD: Attractive person"
    ))),
  # PPC plot for the transformed (normalized) Dyadic TSD: Attractive Person model
  plot(
    check_model(m1b_norm, panel = FALSE, check = "pp_check")$PP_CHECK,
    colors = c("red", "grey30")
  ) +
    labs(title = NULL, subtitle = NULL) +
    theme_tq() +
    facet_wrap(~1, labeller = as_labeller(c(
      "1" = "Transformed (normalized) Dyadic TSD: Attractive person"
    ))),
  labels = "auto", # Automatically label subplots (a, b)
  common.legend = TRUE, legend = "bottom" # Use a common legend at the bottom
)

# Display the final PPC figure
ppc_m1b
```

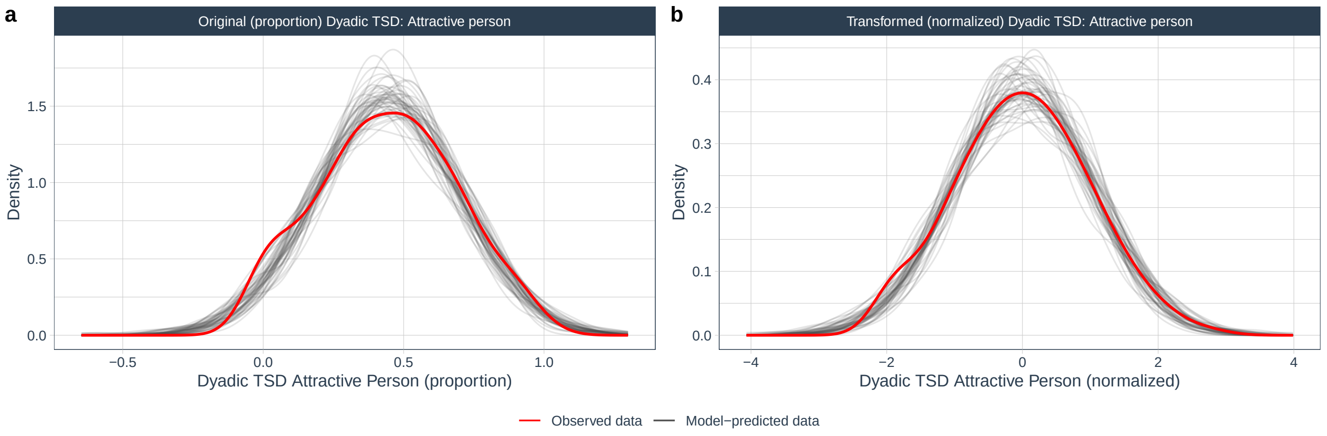

Figure S5. Posterior predictive check. **(a)** Original (proportion) Solitary TSD; **(b)** Transformed (normalized) Solitary TSD. In both panels, red lines represent the observed data, and thin black lines represent 50 iterations of simulated data from each model.

4.1.3.2 Table S9. ANOVA-type table for the interaction between `Relationship type`, and `Gender`

This tables summarizes the results of the model.

```
# Generate an ANOVA table summarizing the effects of Relationship Type and Gender on
# Dyadic TSD Attractive Person
anova.sig.lm(
  model = m1b_norm,
  custom_caption = "Effects of relationship type and gender on Dyadic TSD Attractive Person"
)
```

Table S9. *Effects of relationship type and gender on Dyadic TSD Attractive Person*

Effect	*df*	*F*	*p*	ϵ_p^2
Gender	1, 319	29.85	**< 0.0001**	0.09
Relationship	1, 319	8.20	**0.004**	0.03
Gender × Relationship	1, 319	1.73	0.19	0.00

Note:
Sexual desire was transformed using an ordered quantile normalization (Peterson and Cavanaugh, 2020). Results are Type III ANOVA. $R^2 = 0.122$, $R^2_{adjusted} = 0.114$. Gender = participant's gender (women, men); Relationship = relationship type (stable, single). As effect size, we report partial epsilon squared (ϵ_p^2), a less biased estimate than η^2 (see Albers and Lakens, 2018). Significant effects are in bold.

4.1.3.3 *Post-hoc* comparisons Because the main effects of gender and relationship type, but not their interaction, are significant, we explored these effects using estimated marginal means.

4.1.3.3.1 Table S10. Estimated marginal means and contrasts between participants' gender. Table of estimated marginal means and contrasts between genders. All estimated marginal means and contrasts were calculated using the `emmeans` function from the `emmeans` package (Lenth, 2023).

```
# Compute estimated marginal means (EMMs) for Gender from the model
emms.m1b1 <- emmeans(m1b_norm, ~Gender)

# Convert EMM results to a tibble for easier manipulation
emms.m1b1.tab <- tibble(data.frame(emms.m1b1))

# Compute post-hoc contrasts and format p-values
t.m1b1 <- contr.stars(emms.m1b1) |>
  mutate(p.value = pval.lev(p.value)) # Format p-values for LaTeX

# Merge EMM and contrast results, clean column names, and format output
merge(emms.m1b1.tab, t.m1b1, by = 0, all = TRUE) |>
  select(-c(1, 15)) |> # Remove unnecessary columns
  unite(Contrast, group1, group2, sep = " - ") |> # Create contrast labels
  mutate_at("Contrast", str_replace_all, "NA - NA", " ") |> # Replace missing contrasts
  # Create formatted table
  kable(
    digits = 2, booktabs = TRUE, align = c("l", rep("c", 5), "l", rep("c", 5)),
    linesep = "",
    caption = "Estimated marginal means and contrasts between participants' gender",
    escape = FALSE,
    col.names = c(
      "Gender", "EMM", "$SE$", "$df$", "$2.5\\% CI$", "$97.5\\% CI$",
      "Contrast", "Difference", "$SE$", "$df$", "$t$", "$p$"
    )
  ) |>
  # Add a header separating EMMs and contrasts
  add_header_above(c(" " = 6, "Contrasts" = 6)) |>
  # Apply LaTeX styling for table positioning
  kable_styling(latex_options = c("HOLD_position", "scale_down")) |>
  # Add footnote explaining significance formatting
  footnote(
    general = "Significant effects are in bold.", threeparttable = TRUE,
    footnote_as_chunk = TRUE, escape = FALSE
```

```
  )
```

Table S10. *Estimated marginal means and contrasts between participants' gender*

						Contrasts					
Gender	EMM	*SE*	*df*	2.5%*CI*	97.5%*CI*	Contrast	Difference	*SE*	*df*	*t*	*p*
Women	-0.22	0.07	319	-0.36	-0.09	Women - Men	-0.57	0.1	319	-5.46	**$<$ 0.0001**
Men	0.35	0.08	319	0.19	0.50						

Note: Significant effects are in bold.

4.1.3.3.2 Table S11. Estimated marginal means and contrasts between relationship status.

Table of estimated marginal means and contrasts between relationship status. All estimated marginal means and contrasts were calculated using the `emmeans` function from the `emmeans` package (Lenth, 2023).

```
# Compute estimated marginal means (EMMs) for Relationship Status from the model
emms.m1b2 <- emmeans(m1b_norm, ~Relationship)

# Convert EMM results to a tibble for easier manipulation
emms.m1b2.tab <- tibble(data.frame(emms.m1b2))

# Compute post-hoc contrasts and format p-values
t.m1b2 <- contr.stars(emms.m1b2) |>
  mutate(p.value = pval.lev(p.value)) # Format p-values for LaTeX

# Merge EMM and contrast results, clean column names, and format output
merge(emms.m1b2.tab, t.m1b2, by = 0, all = TRUE) |>
  select(-c(1, 15)) |> # Remove unnecessary columns
  unite(Contrast, group1, group2, sep = " - ") |> # Create contrast labels
  mutate_at("Contrast", str_replace_all, "NA - NA", " ") |> # Replace missing contrasts
  # Create formatted table
  kable(
    digits = 2, booktabs = TRUE, align = c("l", rep("c", 5), "l", rep("c", 5)),
    linesep = "",
    caption = "Estimated marginal means and contrasts between relationship status",
    escape = FALSE,
    col.names = c(
      "Relationship type", "EMM", "$SE$", "$df$", "$2.5\\% CI$", "$97.5\\% CI$",
      "Contrast", "Difference", "$SE$", "$df$", "$t$", "$p$"
    )
  ) |>
  # Add a header separating EMMs and contrasts
  add_header_above(c(" " = 6, "Contrasts" = 6)) |>
  # Apply LaTeX styling for table positioning
  kable_styling(latex_options = c("HOLD_position", "scale_down")) |>
  # Add footnote explaining significance formatting
  footnote(
    general = "Significant effects are in bold.", threeparttable = TRUE,
    footnote_as_chunk = TRUE, escape = FALSE
  )
```

Table S11. *Estimated marginal means and contrasts between relationship status*

						Contrasts					
Relationship type	EMM	*SE*	*df*	2.5%*CI*	97.5%*CI*	Contrast	Difference	*SE*	*df*	*t*	*p*
Stable	-0.09	0.07	319	-0.22	0.05	Stable - Single	-0.3	0.1	319	-2.86	**0.0045**
Single	0.21	0.08	319	0.06	0.36						

Note: Significant effects are in bold.

4.1.3.3.3 Table S12. Estimated marginal means and contrasts between gender by relationship status. Table of estimated marginal means and contrasts between gender by relationship status. All estimated marginal means and contrasts were calculated using the `emmeans` function from the `emmeans` package (Lenth, 2023).

```
# Compute estimated marginal means (EMMs) for Gender within each Relationship Status
emms.m1b3 <- emmeans(m1b_norm, ~ Gender | Relationship)

# Convert EMM results to a tibble for easier manipulation
emms.m1b3.tab <- tibble(data.frame(emms.m1b3))

# Compute post-hoc contrasts and format p-values
t.m1b3 <- contr.stars(emms.m1b3) |>
  mutate(p.value = pval.lev(p.value)) # Format p-values for LaTeX

# Insert NA rows to maintain table structure for grouped relationship status display
t.m1b3.f <- t.m1b3 |>
  insertRows(2, new = NA) |> # Insert empty row after first contrast
  insertRows(4, new = NA) # Insert empty row after second contrast

# Merge EMM and contrast results, clean column names, and format output
merge(emms.m1b3.tab, t.m1b3.f, by = 0, all = TRUE) |>
  select(-c(1, 3, 11, 17)) |> # Remove unnecessary columns
  drop_na(Gender) |> # Ensure Gender column is complete
  unite(Contrast, group1, group2, sep = " - ") |> # Create contrast labels
  mutate_at("Contrast", str_replace_all, "NA - NA", "") |> # Replace missing contrasts
  # Create formatted table
  kable(
    digits = 2, booktabs = TRUE, align = c("l", "l", rep("c", 5), "l", rep("c", 5)),
    linesep = "",
    caption = "Estimated marginal means and contrasts between gender by relationship status",
    escape = FALSE,
    col.names = c(
      "Gender", "EMM", "$SE$", "$df$", "$2.5\\% CI$", "$97.5\\% CI$",
      "Contrast", "Difference", "$SE$", "$df$", "$t$", "$p$"
    )
  ) |>
  # Add grouped row labels for Relationship Status
  pack_rows("Relationship status: Stable",
    start_row = 1, end_row = 2,
    bold = FALSE, background = "lightgray"
  ) |>
  pack_rows("Relationship status: Single",
    start_row = 3, end_row = 4,
    bold = FALSE, background = "lightgray"
  ) |>
  # Add a header separating EMMs and contrasts
  add_header_above(c(" " = 6, "Contrasts" = 6)) |>
  # Apply LaTeX styling for table positioning
```

```
  kable_styling(latex_options = c("HOLD_position", "scale_down")) |>
  # Add footnote explaining significance formatting
  footnote(
    general = "Significant effects are in bold.", threeparttable = TRUE,
    footnote_as_chunk = TRUE, escape = FALSE
  )
```

Table S12. *Estimated marginal means and contrasts between gender by relationship status*

						Contrasts					
Gender	EMM	*SE*	*df*	2.5%*CI*	97.5%*CI*	Contrast	Difference	*SE*	*df*	*t*	*p*
Relationship status: Stable											
Women	-0.44	0.09	319	-0.62	-0.26	Women - Men	-0.71	0.14	319	-5.00	**< 0.0001**
Men	0.27	0.11	319	0.05	0.48						
Relationship status: Single											
Women	0.00	0.10	319	-0.21	0.20	Women - Men	-0.43	0.15	319	-2.82	**0.0051**
Men	0.43	0.11	319	0.21	0.65						

Note: Significant effects are in bold.

4.1.3.4 Figure S6. Effects of gender and relationship type on Dyadic TSD Attractive Person This figure summarizes the results of hypothesis 1b.

```
# Plot (a): Main effect of Gender on Dyadic TSD Attractive Person
h1b1 <- ggplot(dat_m1, aes(
  x = Gender, y = `Dyadic TSD Attractive Person (normalized)`, color = Gender
)) +
  scale_color_manual(values = color.Gender) +
  scale_fill_manual(values = color.Gender) +
  geom_linerange(
    data = emms.m1b1.tab |> rename("Dyadic TSD Attractive Person (normalized)" = emmean),
    mapping = aes(ymin = lower.CL, ymax = upper.CL)
  ) +
  geom_point(
    data = emms.m1b1.tab |> rename("Dyadic TSD Attractive Person (normalized)" = emmean),
    position = position_dodge(0.1), size = 3
  ) +
  stat_pvalue_manual(t.m1b1, label = "p.signif", y.position = 0.6, tip.length = 0) +
  guides(color = "none") +
  theme_tq()

# Plot (b): Main effect of Relationship on Dyadic TSD Attractive Person
h1b2 <- ggplot(dat_m1, aes(
  x = Relationship, y = `Dyadic TSD Attractive Person (normalized)`, color = Relationship
)) +
  scale_color_manual(values = color.Relationship) +
  scale_fill_manual(values = color.Relationship) +
  geom_linerange(
    data = emms.m1b2.tab |> rename("Dyadic TSD Attractive Person (normalized)" = emmean),
    mapping = aes(ymin = lower.CL, ymax = upper.CL)
  ) +
  geom_point(
    data = emms.m1b2.tab |> rename("Dyadic TSD Attractive Person (normalized)" = emmean),
    position = position_dodge(0.1), size = 3
  ) +
  stat_pvalue_manual(t.m1b2, label = "p.signif", y.position = 0.45, tip.length = 0) +
```

```
  guides(color = "none") +
  theme_tq()

# Plot (c): Gender × Relationship Interaction on Dyadic TSD Attractive Person
h1b3 <- ggplot(dat_m1, aes(
  x = Gender, y = `Dyadic TSD Attractive Person (normalized)`, color = Gender
)) +
  scale_color_manual(values = color.Gender) +
  scale_fill_manual(values = color.Gender) +
  facet_wrap(~Relationship) +
  geom_linerange(
    data = emms.m1b3.tab |> rename("Dyadic TSD Attractive Person (normalized)" = emmean),
    mapping = aes(ymin = lower.CL, ymax = upper.CL)
  ) +
  geom_point(
    data = emms.m1b3.tab |> rename("Dyadic TSD Attractive Person (normalized)" = emmean),
    position = position_dodge(0.1), size = 3
  ) +
  stat_pvalue_manual(t.m1b3, label = "p.signif", y.position = c(0.6, 0.7), tip.length = 0) +
  guides(color = "none") +
  theme_tq()

# Combine the three plots into a single figure
p1b <- ggarrange(h1b1, h1b2 + labs(y = NULL), h1b3 + labs(y = NULL),
  ncol = 3, labels = "auto", widths = c(1, 1, 1.5) # Adjust widths for better alignment
)

# Display the final figure
p1b
```

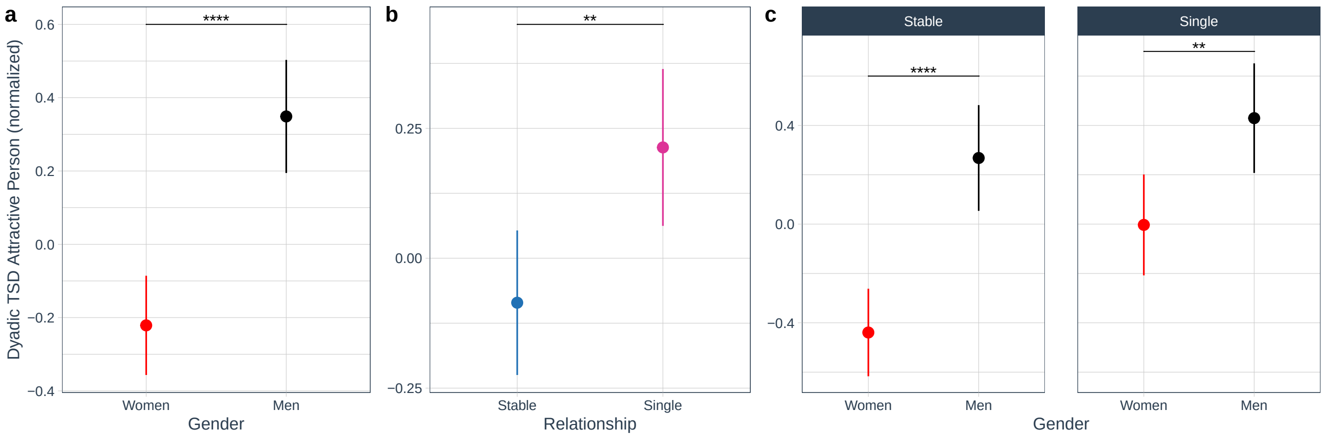

Figure S6. Effects of gender and relationship type on Dyadic TSD Attractive Person. Dyadic TSD Attractive Person was transformed using ordered quantile normalization (Peterson & Cavanaugh, 2020). **(a)** Simple comparison between sexual desire by gender (for detailed results, see Table S10); **(b)** Simple comparison between relationship status levels (for detailed results, see Table S11); **(c)** Interaction between relationship type and relationship status (see Table S9; for detailed results, see Table S12). Dots and bars represent estimated marginal means and 95% CI. In all cases, significant effects are represented with lines and stars: *$p < 0.05$, **$p < 0.01$, ***$p < 0.001$, ****$p < 0.0001$.

4.1.4 Hypothesis 1c: Dyadic TSD (Partner)

4.1.4.1 Model the effects of relationship type and gender on Dyadic TSD: Partner We fitted models with both the original (proportion; `m1c_prop`) and transformed (normalized; `m1c_norm`) TSD scores, and performed

posterior predictive checks (PPCs). As shown elsewhere (e.g., Gabry et al., 2019), if simulated data from one model are more similar to the observed outcome, that model is likely to be preferred.

```
# Set contrast options for sum coding (useful for ANOVA-like interpretation)
options(contrasts = c("contr.sum", "contr.poly"))

# Fit a linear model using the original proportion-based Dyadic TSD (Partner) scores
m1c_prop <- lm(
  `Dyadic TSD Partner (proportion)` ~ Gender * Relationship,
  data = dat_m1
)

# Fit a linear model using the normalized Dyadic TSD (Partner) scores
m1c_norm <- lm(
  `Dyadic TSD Partner (normalized)` ~ Gender * Relationship,
  data = dat_m1
)
```

4.1.4.1.1 Figure S7: Posterior predictive checks (PPCs) for Hypothesis 1c. PPCs were performed using the `check_model` function from the `performance` package (Lüdecke et al., 2021), and reported in Fig. S7. Simulated data from the normalized Solitary TSD model (Fig. S7b) are more similar to the observed outcome, so this model is preferred.

```
# Perform posterior predictive checks (PPC) and arrange them into a single figure
ppc_m1c <- ggarrange(
  # PPC plot for the original (proportion) Dyadic TSD: Partner model
  plot(
    check_model(m1c_prop, panel = FALSE, check = "pp_check")$PP_CHECK,
    colors = c("red", "grey30") # Red for observed data, grey for simulated data
  ) +
    labs(title = NULL, subtitle = NULL) +
    theme_tq() +
    facet_wrap(~1, labeller = as_labeller(c(
      "1" = "Original (proportion) Dyadic TSD: Partner"
    ))),
  # PPC plot for the transformed (normalized) Dyadic TSD: Partner model
  plot(
    check_model(m1c_norm, panel = FALSE, check = "pp_check")$PP_CHECK,
    colors = c("red", "grey30")
  ) +
    labs(title = NULL, subtitle = NULL) +
    theme_tq() +
    facet_wrap(~1, labeller = as_labeller(c(
      "1" = "Transformed (normalized) Dyadic TSD: Partner"
    ))),
  labels = "auto", # Automatically label subplots (a, b)
  common.legend = TRUE, legend = "bottom" # Use a common legend at the bottom
)

# Display the final PPC figure
ppc_m1c
```

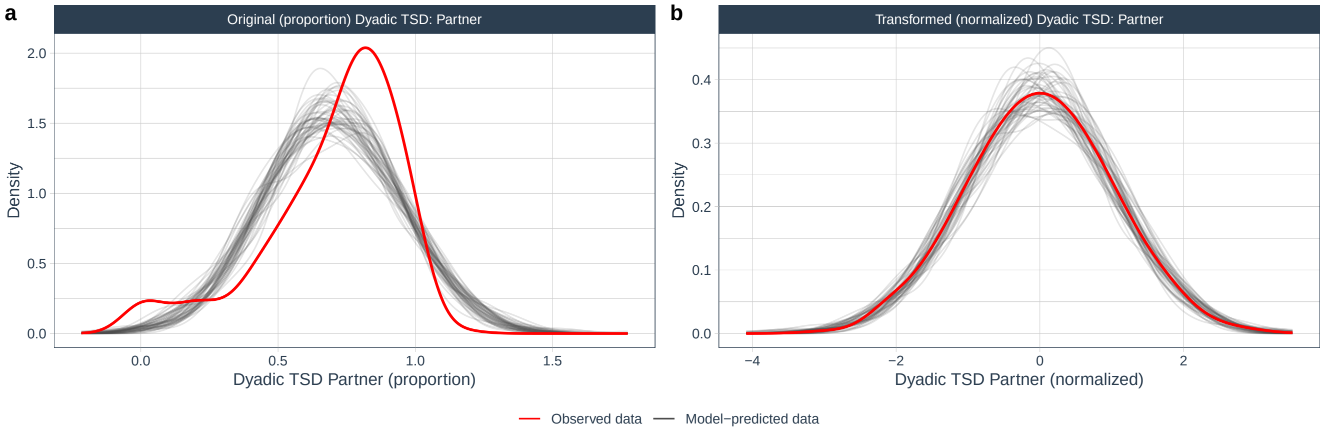

Figure S7. Posterior predictive check. **(a)** Original (proportion) Solitary TSD; **(b)** Transformed (normalized) Solitary TSD. In both panels, red lines represent the observed data, and thin black lines represent 50 iterations of simulated data from each model.

4.1.4.2 Table S13. ANOVA-type table for the interaction between `Relationship type`, and `Gender`

This tables summarizes the results of the model.

```
# Generate an ANOVA table summarizing the effects of Relationship Type and Gender on
# Dyadic TSD Partner
anova.sig.lm(
  model = m1c_norm,
  custom_caption = "Effects of relationship type and gender on Dyadic TSD Partner"
)
```

Table S13. *Effects of relationship type and gender on Dyadic TSD Partner*

Effect	*df*	*F*	*p*	ϵ_p^2
Gender	1, 316	15.49	$<$ **0.001**	0.0365
Relationship	1, 316	31.60	$<$ **0.0001**	0.09
Gender × Relationship	1, 316	0.00	0.98	$<$ 0.0001

Note:
Sexual desire was transformed using an ordered quantile normalization (Peterson and Cavanaugh, 2020). Results are Type III ANOVA. $R^2 = 0.125$, $R^2_{adjusted} = 0.117$. Gender = participant's gender (women, men); Relationship = relationship type (stable, single). As effect size, we report partial epsilon squared (ϵ_p^2), a less biased estimate than η^2 (see Albers and Lakens, 2018). Significant effects are in bold.

4.1.4.3 *Post-hoc* comparisons

Because the main effects of gender and relationship type, but not their interaction, are significant, we explored these effects using estimated marginal means.

4.1.4.3.1 Table S14. Estimated marginal means and contrasts between participants' gender.

Table of estimated marginal means and contrasts between genders. All estimated marginal means and contrasts were calculated using the `emmeans` function from the `emmeans` package (Lenth, 2023).

```
# Compute estimated marginal means (EMMs) for Gender from the model
emms.m1c1 <- emmeans(m1c_norm, ~Gender)

# Convert EMM results to a tibble for easier manipulation
emms.m1c1.tab <- tibble(data.frame(emms.m1c1))
```

```
# Compute post-hoc contrasts and format p-values
t.m1c1 <- contr.stars(emms.m1c1) |>
  mutate(p.value = pval.lev(p.value)) # Format p-values for LaTeX

# Merge EMM and contrast results, clean column names, and format output
merge(emms.m1c1.tab, t.m1c1, by = 0, all = TRUE) |>
  select(-c(1, 15)) |> # Remove unnecessary columns
  unite(Contrast, group1, group2, sep = " - ") |> # Create contrast labels
  mutate_at("Contrast", str_replace_all, "NA - NA", " ") |> # Replace missing contrasts
  # Create formatted table
  kable(
    digits = 2, booktabs = TRUE, align = c("l", rep("c", 5), "l", rep("c", 5)),
    linesep = "",
    caption = "Estimated marginal means and contrasts between participants' gender",
    escape = FALSE,
    col.names = c(
      "Gender", "EMM", "$SE$", "$df$", "$2.5\\% CI$", "$97.5\\% CI$",
      "Contrast", "Difference", "$SE$", "$df$", "$t$", "$p$"
    )
  ) |>
  # Add a header separating EMMs and contrasts
  add_header_above(c(" " = 6, "Contrasts" = 6)) |>
  # Apply LaTeX styling for table positioning
  kable_styling(latex_options = c("HOLD_position", "scale_down")) |>
  # Add footnote explaining significance formatting
  footnote(
    general = "Significant effects are in bold.", threeparttable = TRUE,
    footnote_as_chunk = TRUE, escape = FALSE
  )
```

Table S14. *Estimated marginal means and contrasts between participants' gender*

						Contrasts					
Gender	EMM	*SE*	*df*	2.5%*CI*	97.5%*CI*	Contrast	Difference	*SE*	*df*	*t*	*p*
Women	-0.21	0.07	316	-0.35	-0.07	Women - Men	-0.42	0.11	316	-3.94	$<$ **0.001**
Men	0.20	0.08	316	0.05	0.36						

Note: Significant effects are in bold.

4.1.4.3.2 Table S15. Estimated marginal means and contrasts between relationship status.

Table of estimated marginal means and contrasts between relationship status. All estimated marginal means and contrasts were calculated using the `emmeans` function from the `emmeans` package (Lenth, 2023).

```
# Compute estimated marginal means (EMMs) for Relationship Status from the model
emms.m1c2 <- emmeans(m1c_norm, ~Relationship)

# Convert EMM results to a tibble for easier manipulation
emms.m1c2.tab <- tibble(data.frame(emms.m1c2))

# Compute post-hoc contrasts and format p-values
t.m1c2 <- contr.stars(emms.m1c2) |>
  mutate(p.value = pval.lev(p.value)) # Format p-values for LaTeX

# Merge EMM and contrast results, clean column names, and format output
merge(emms.m1c2.tab, t.m1c2, by = 0, all = TRUE) |>
  select(-c(1, 15)) |> # Remove unnecessary columns
```

```
  unite(Contrast, group1, group2, sep = " - ") |> # Create contrast labels
  mutate_at("Contrast", str_replace_all, "NA - NA", " ") |> # Replace missing contrasts
  # Create formatted table
  kable(
    digits = 2, booktabs = TRUE, align = c("l", rep("c", 5), "l", rep("c", 5)),
    linesep = "",
    caption = "Estimated marginal means and contrasts between relationship status",
    escape = FALSE,
    col.names = c(
      "Relationship type", "EMM", "$SE$", "$df$", "$2.5\\% CI$", "$97.5\\% CI$",
      "Contrast", "Difference", "$SE$", "$df$", "$t$", "$p$"
    )
  ) |>
  # Add a header separating EMMs and contrasts
  add_header_above(c(" " = 6, "Contrasts" = 6)) |>
  # Apply LaTeX styling for table positioning
  kable_styling(latex_options = c("HOLD_position", "scale_down")) |>
  # Add footnote explaining significance formatting
  footnote(
    general = "Significant effects are in bold.", threeparttable = TRUE,
    footnote_as_chunk = TRUE, escape = FALSE
  )
```

Table S15. *Estimated marginal means and contrasts between relationship status*

						Contrasts					
Relationship type	EMM	*SE*	*df*	2.5%*CI*	97.5%*CI*	Contrast	Difference	*SE*	*df*	*t*	*p*
Stable	0.29	0.07	316	0.15	0.43	Stable - Single	0.6	0.11	316	5.62	$<$ **0.0001**
Single	-0.30	0.08	316	-0.46	-0.15						

Note: Significant effects are in bold.

4.1.4.3.3 Table S16. Estimated marginal means and contrasts between gender by relationship status.

Table of estimated marginal means and contrasts between gender by relationship status. All estimated marginal means and contrasts were calculated using the `emmeans` function from the `emmeans` package (Lenth, 2023).

```
# Compute estimated marginal means (EMMs) for Gender within each Relationship Status
emms.m1c3 <- emmeans(m1c_norm, ~ Gender | Relationship)

# Convert EMM results to a tibble for easier manipulation
emms.m1c3.tab <- tibble(data.frame(emms.m1c3))

# Compute post-hoc contrasts and format p-values
t.m1c3 <- contr.stars(emms.m1c3) |>
  mutate(p.value = pval.lev(p.value)) # Format p-values for LaTeX

# Insert NA rows to maintain table structure for grouped relationship status display
t.m1c3.f <- t.m1c3 |>
  insertRows(2, new = NA) |> # Insert empty row after first contrast
  insertRows(4, new = NA) # Insert empty row after second contrast

# Merge EMM and contrast results, clean column names, and format output
merge(emms.m1c3.tab, t.m1c3.f, by = 0, all = TRUE) |>
  select(-c(1, 3, 11, 17)) |> # Remove unnecessary columns
  drop_na(Gender) |> # Ensure Gender column is complete
  unite(Contrast, group1, group2, sep = " - ") |> # Create contrast labels
  mutate_at("Contrast", str_replace_all, "NA - NA", "") |> # Replace missing contrasts
```

```
# Create formatted table
kable(
  digits = 2, booktabs = TRUE, align = c("l", "l", rep("c", 5), "l", rep("c", 5)),
  linesep = "",
  caption = "Estimated marginal means and contrasts between gender by relationship status",
  escape = FALSE,
  col.names = c(
    "Gender", "EMM", "$SE$", "$df$", "$2.5\\% CI$", "$97.5\\% CI$",
    "Contrast", "Difference", "$SE$", "$df$", "$t$", "$p$"
  )
) |>
# Add grouped row labels for Relationship Status
pack_rows("Relationship status: Stable",
  start_row = 1, end_row = 2,
  bold = FALSE, background = "lightgray"
) |>
pack_rows("Relationship status: Single",
  start_row = 3, end_row = 4,
  bold = FALSE, background = "lightgray"
) |>
# Add a header separating EMMs and contrasts
add_header_above(c(" " = 6, "Contrasts" = 6)) |>
# Apply LaTeX styling for table positioning
kable_styling(latex_options = c("HOLD_position", "scale_down")) |>
# Add footnote explaining significance formatting
footnote(
  general = "Significant effects are in bold.", threeparttable = TRUE,
  footnote_as_chunk = TRUE, escape = FALSE
)
```

Table S16. *Estimated marginal means and contrasts between gender by relationship status*

						Contrasts					
Gender	EMM	*SE*	*df*	2.5%*CI*	97.5%*CI*	Contrast	Difference	*SE*	*df*	*t*	*p*
Relationship status: Stable											
Women	0.09	0.09	316	-0.09	0.27	Women - Men	-0.41	0.14	316	-2.90	**0.004**
Men	0.50	0.11	316	0.28	0.72						
Relationship status: Single											
Women	-0.51	0.11	316	-0.72	-0.30	Women - Men	-0.42	0.16	316	-2.68	**0.0077**
Men	-0.09	0.11	316	-0.32	0.13						

Note: Significant effects are in bold.

4.1.4.4 Figure S8. Effects of gender and relationship type on Dyadic TSD Partner

This figure summarizes the results of hypothesis 1c.

```
# Plot (a): Main effect of Gender on Dyadic TSD Partner
h1c1 <- ggplot(dat_m1, aes(
  x = Gender, y = `Dyadic TSD Partner (normalized)`, color = Gender
)) +
  scale_color_manual(values = color.Gender) +
  scale_fill_manual(values = color.Gender) +
  geom_linerange(
    data = emms.m1c1.tab |> rename("Dyadic TSD Partner (normalized)" = emmean),
    mapping = aes(ymin = lower.CL, ymax = upper.CL)
  ) +
```

```
  geom_point(
    data = emms.m1c1.tab |> rename("Dyadic TSD Partner (normalized)" = emmean),
    position = position_dodge(0.1), size = 3
  ) +
  stat_pvalue_manual(t.m1c1, label = "p.signif", y.position = 0.4, tip.length = 0) +
  guides(color = "none") +
  theme_tq()

# Plot (b): Main effect of Relationship on Dyadic TSD Partner
h1c2 <- ggplot(dat_m1, aes(
  x = Relationship, y = `Dyadic TSD Partner (normalized)`, color = Relationship
)) +
  scale_color_manual(values = color.Relationship) +
  scale_fill_manual(values = color.Relationship) +
  geom_linerange(
    data = emms.m1c2.tab |> rename("Dyadic TSD Partner (normalized)" = emmean),
    mapping = aes(ymin = lower.CL, ymax = upper.CL)
  ) +
  geom_point(
    data = emms.m1c2.tab |> rename("Dyadic TSD Partner (normalized)" = emmean),
    position = position_dodge(0.1), size = 3
  ) +
  stat_pvalue_manual(t.m1c2, label = "p.signif", y.position = 0.5, tip.length = 0) +
  guides(color = "none") +
  theme_tq()

# Plot (c): Gender × Relationship Interaction on Dyadic TSD Partner
h1c3 <- ggplot(dat_m1, aes(
  x = Gender, y = `Dyadic TSD Partner (normalized)`, color = Gender
)) +
  scale_color_manual(values = color.Gender) +
  scale_fill_manual(values = color.Gender) +
  facet_wrap(~Relationship) +
  geom_linerange(
    data = emms.m1c3.tab |> rename("Dyadic TSD Partner (normalized)" = emmean),
    mapping = aes(ymin = lower.CL, ymax = upper.CL)
  ) +
  geom_point(
    data = emms.m1c3.tab |> rename("Dyadic TSD Partner (normalized)" = emmean),
    position = position_dodge(0.1), size = 3
  ) +
  stat_pvalue_manual(t.m1c3, label = "p.signif", y.position = c(0.8, 0.2), tip.length = 0) +
  guides(color = "none") +
  theme_tq()

# Combine the three plots into a single figure
p1c <- ggarrange(h1c1, h1c2 + labs(y = NULL), h1c3 + labs(y = NULL),
  ncol = 3, labels = "auto", widths = c(1, 1, 1.5) # Adjust widths for better alignment
)

# Display the final figure
p1c
```

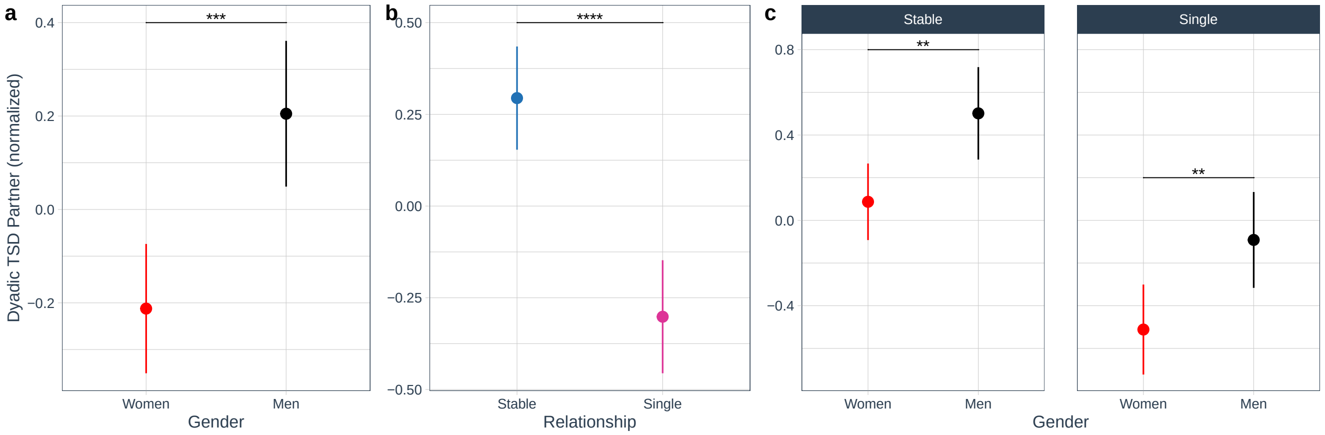

Figure S8. Effects of gender and relationship type on Dyadic TSD Partner. Dyadic TSD Partner was transformed using ordered quantile normalization (Peterson & Cavanaugh, 2020). **(a)** Simple comparison between sexual desire by gender (for detailed results, see Table S14); **(b)** Simple comparison between relationship status levels (for detailed results, see Table S15); **(c)** Interaction between relationship type and relationship status (see Table S13; for detailed results, see Table S16). Dots and bars represent estimated marginal means and 95% CI. In all cases, significant effects are represented with lines and stars: *$p < 0.05$, **$p < 0.01$, ***$p < 0.001$, ****$p < 0.0001$.

4.2 Data filtering for hypotheses 2 and 3.

To avoid over-complicating the models, first we tested whether the effects of stimuli on sexual arousal were stronger depending on the content of the stimuli (erotic versus non-erotic). This was, in fact, the case.

4.2.1 Table S17. ANOVA-type table for the effects of stimuli content, gender and stimuli gender on Subjective sexual arousal

We fitted a linear mixed model with Gender, Stimuli gender, Stimuli content, and their interactions, as fixed effects for Subjective sexual arousal and including, as random effects, random intercepts per stimulus, as well as random intercepts and slopes for the effect of stimuli content.

```
# Fit a linear mixed model predicting Subjective Sexual Arousal
# Fixed effects: Gender, Stimuli gender, Stimuli content, and their interactions
# Random effects: Intercepts per stimulus; intercepts & slopes for stimuli content per participant
m_stim_cont <- lmer(
  `Subjective sexual arousal` ~ Gender * `Stimuli gender` * `Stimuli content` +
    (1 | `Stimuli code`) +
    (1 + `Stimuli content` * `Stimuli gender` | Participant),
  data = dat,
  control = lmerControl(optimizer = "bobyqa") # Use "bobyqa" optimizer for better convergence
)

# Generate ANOVA table summarizing the model results
anova.sig.lmer(
  model = m_stim_cont,
  custom_caption = "Effects of relationship type, gender and stimuli gender on Dyadic TSD Partner"
)
```

Table S17. *Effects of relationship type, gender and stimuli gender on Dyadic TSD Partner*

Effect	*df*	*F*	*p*	ϵ_p^2
Gender	1, 321	42.47	$<$ **0.0001**	0.11
Stimuli gender	1, 447	96.15	$<$ **0.0001**	0.18
Stimuli content	1, 363.12	86.50	$<$ **0.0001**	0.19
Gender × Stimuli gender	1, 321	471.68	$<$ **0.0001**	0.59
Gender × Stimuli content	1, 321	5.02	**0.0257**	0.01
Stimuli gender × Stimuli content	1, 286.22	21.51	$<$ **0.0001**	0.07
Gender × Stimuli gender × Stimuli content	1, 321	116.42	$<$ **0.0001**	0.26

Note: Results are Type III ANOVA. $R^2_{conditional} = 0.734$, $R^2_{marginal} = 0.314$. As effect size, we report partial epsilon squared (ϵ_p^2), a less biased estimate than η^2 (see Albers and Lakens, 2018). Significant effects are in bold.

The effects of stimuli on sexual arousal were stronger for erotic compared to non-erotic stimuli; to illustrate this, we compared the (within-subject) difference in reported sexual arousal between stimuli genderes, for women and men. This difference was larger when viewing erotic than non-erotic stimuli in both women (erotic: 0.77, non-erotic: 0.57) but especially in men (erotic: 2.75, non-erotic: 1.60; see Table S18 and Fig. S9). Considering this, we tested all predictions of hypotheses 2 and 3 only on responses to erotic stimuli.

4.2.2 Table S18. Estimated marginal means and contrasts between subjective sexual arousal depending on stimuli gender, by stimuli content and participant gender.

Table of estimated marginal means and contrasts between between subjective sexual arousal depending on stimuli gender, by stimuli content and participant gender. All estimated marginal means and contrasts were calculated using the `emmeans` function from the `emmeans` package (Lenth, 2023).

```r
# Compute estimated marginal means (EMMs) for Stimuli Gender by Stimuli Content & Gender
emms.stim_cont <- emmeans(m_stim_cont, pairwise ~ `Stimuli gender` | `Stimuli content` + Gender,
  adjust = "bonferroni", # Apply Bonferroni correction for multiple comparisons
  lmer.df = "satterthwaite" # Use Satterthwaite approximation for degrees of freedom
)

# Convert EMM results to a tibble and rename columns for clarity
emms.stim_cont.tab <- tibble(data.frame(emms.stim_cont$emmeans)) |>
  rename(
    "Subjective sexual arousal" = emmean,
    "Stimuli content" = Stimuli.content,
    "Stimuli gender" = Stimuli.gender
  )

# Compute post-hoc contrasts and format p-values
t.stim_cont <- contr.stars(emms.stim_cont) |>
  mutate(p.value = pval.lev(p.value)) # Format p-values for LaTeX

# Insert NA rows to maintain structure for gender and stimuli content groupings
t.stim_cont.f <- t.stim_cont |>
  insertRows(2, new = NA) |>
  insertRows(4, new = NA) |>
  insertRows(6, new = NA) |>
  insertRows(8, new = NA)

# Merge EMM and contrast results, clean column names, and format output
merge(emms.stim_cont.tab, t.stim_cont.f, by = 0, all = TRUE) |>
  select(-c(1, 3, 4, 12, 13, 19)) |> # Remove unnecessary columns
  drop_na("Stimuli gender") |> # Ensure Stimuli gender column is complete
```

```
unite(Contrast, group1, group2, sep = " - ") |> # Create contrast labels
mutate_at("Contrast", str_replace_all, "NA - NA", " ") |> # Replace missing contrasts
mutate(across(c(df.x, df.y), as.character)) |> # Convert df columns to character
mutate(across(c(df.x, df.y), str_replace_all, "Inf", "$\\\\infty$")) |> # Format infinity df

# Create formatted table
kable(
  digits = 2, booktabs = TRUE, align = c("l", "l", rep("c", 5), "l", rep("c", 5)),
  linesep = "",
  caption = "Estimated marginal means for the three dimensions of sexual
  desire by relationship status",
  escape = FALSE,
  col.names = c(
    "Stimuli gender", "EMM", "$SE$", "$df$", "$2.5\\% CI$", "$97.5\\% CI$",
    "Contrast", "Difference", "$SE$", "$df$", "$z$", "$p$"
  )
) |>
# Add grouped row labels for Gender and Stimuli Content
pack_rows("Gender: Women - Stimuli content: Erotic",
  start_row = 1, end_row = 2,
  bold = FALSE, background = "lightgray"
) |>
pack_rows("Gender: Women - Stimuli content: Non-erotic",
  start_row = 3, end_row = 4,
  bold = FALSE, background = "lightgray"
) |>
pack_rows("Gender: Men - Stimuli content: Erotic",
  start_row = 5, end_row = 6,
  bold = FALSE, background = "lightgray"
) |>
pack_rows("Gender: Men - Stimuli content: Non-erotic",
  start_row = 7, end_row = 8,
  bold = FALSE, background = "lightgray"
) |>
# Add a header separating EMMs and contrasts
add_header_above(c(" " = 6, "Contrasts" = 6)) |>
# Apply LaTeX styling for table positioning
kable_styling(latex_options = c("HOLD_position", "scale_down")) |>
# Add footnote explaining statistical adjustments
footnote(
  general = "EMM = estimated marginal mean.
         Degrees of freedom ($df$) are asymptotic.
         Bonferroni adjustment was used.",
  threeparttable = TRUE, footnote_as_chunk = TRUE, escape = FALSE
)
```

Table S18. *Estimated marginal means for the three dimensions of sexual desire by relationship status*

Stimuli gender	EMM	*SE*	*df*	2.5%*CI*	97.5%*CI*	Contrasts					
						Contrast	Difference	*SE*	*df*	*z*	*p*
Gender: Women - Stimuli content: Erotic											
Female	1.46	0.10	∞	1.25	1.66	Female - Male	-0.77	0.11	∞	-6.80	**$<$ 0.0001**
Male	2.23	0.08	∞	2.08	2.38						
Gender: Women - Stimuli content: Non-erotic											
Female	1.12	0.09	∞	0.94	1.30	Female - Male	-0.57	0.11	∞	-5.27	**$<$ 0.0001**
Male	1.69	0.07	∞	1.56	1.82						
Gender: Men - Stimuli content: Erotic											
Female	3.84	0.12	∞	3.61	4.07	Female - Male	2.75	0.13	∞	21.60	**$<$ 0.0001**
Male	1.09	0.09	∞	0.92	1.26						
Gender: Men - Stimuli content: Non-erotic											
Female	2.65	0.10	∞	2.45	2.85	Female - Male	1.60	0.12	∞	13.44	**$<$ 0.0001**
Male	1.05	0.07	∞	0.91	1.19						

Note: EMM = estimated marginal mean. Degrees of freedom (*df*) are asymptotic. Bonferroni adjustment was used.

4.2.3 Figure S9. Effects of stimuli content (erotic, non-erotic) on subjective sexual arousal

This figure summarizes the results of the model to determine whether the effects of stimuli on sexual arousal were stronger depending on the content of the stimuli (erotic versus non-erotic).

```r
# Prepare data for vertical comparison lines between Male and Female
diff_data <- emms.stim_cont.tab |>
  select(`Stimuli gender`, Gender, `Stimuli content`, `Subjective sexual arousal`) |>
  pivot_wider(names_from = `Stimuli gender`, values_from = `Subjective sexual arousal`) |>
  mutate(
    ymin = Male, # Start of line at Male's mean arousal
    ymax = Female # End of line at Female's mean arousal
  ) |>
  mutate(
    # Define custom x positions slightly offset for better readability
    x_pos = rep(c(
      as.numeric(as.factor(`Stimuli content`[1])) - 0.25,
      as.numeric(as.factor(`Stimuli content`[2])) + 0.25
    ), 2)
  )

# Create the plot
ggplot(emms.stim_cont.tab, aes(
  x = `Stimuli gender`, y = `Subjective sexual arousal`,
  color = `Stimuli content`
)) +
  # Separate plots for each Gender
  facet_wrap(~Gender) +
  # Set custom colors for Stimuli content
  scale_color_manual(values = color.Content) +
  scale_fill_manual(values = color.Content) +
  # Add confidence interval ranges
  geom_linerange(
    data = emms.stim_cont.tab,
    mapping = aes(ymin = asymp.LCL, ymax = asymp.UCL),
    position = position_dodge(0.5)
  ) +
  # Add individual data points with position dodge to avoid overlap
```

```
geom_point(
  data = emms.stim_cont.tab,
  position = position_dodge(0.5),
  size = 3
) +
# Add statistical significance annotations
stat_pvalue_manual(t.stim_cont,
  label = "p.signif",
  y.position = c(2.7, 3, 4.2, 3), # Adjusted y positions for clarity
  tip.length = 0,
  color = "Stimuli content",
  position = position_dodge(0.5)
) +
# Add vertical dotted lines WITHOUT arrows
geom_segment(
  data = diff_data,
  aes(
    x = x_pos, xend = x_pos,
    y = ymin, yend = ymax,
    color = `Stimuli content`
  ),
  linewidth = 0.5,
  linetype = "dotted"
) + # Dotted lines
# Add SOLID arrows separately, with NO line
geom_segment(
  data = diff_data,
  aes(
    x = x_pos, xend = x_pos,
    y = ymin, yend = ymax,
    color = `Stimuli content`
  ),
  linetype = "solid", # Make sure arrows are solid
  linewidth = 0, # Hide the line itself
  arrow = arrow(length = unit(0.3, "cm"), type = "closed", ends = "both")
) +
# Rotated & Centered Difference Labels on the vertical lines
geom_text(
  data = diff_data,
  aes(
    x = x_pos - 0.06, y = (ymin + ymax) / 2,
    label = abs(round(ymax - ymin, 2)),
    color = `Stimuli content`
  ),
  angle = 90, # Rotate text vertically
  hjust = 0.5, # Center horizontally
  vjust = 0.5, # Center vertically on the line
  size = 2.5
) +
theme_tq()
```

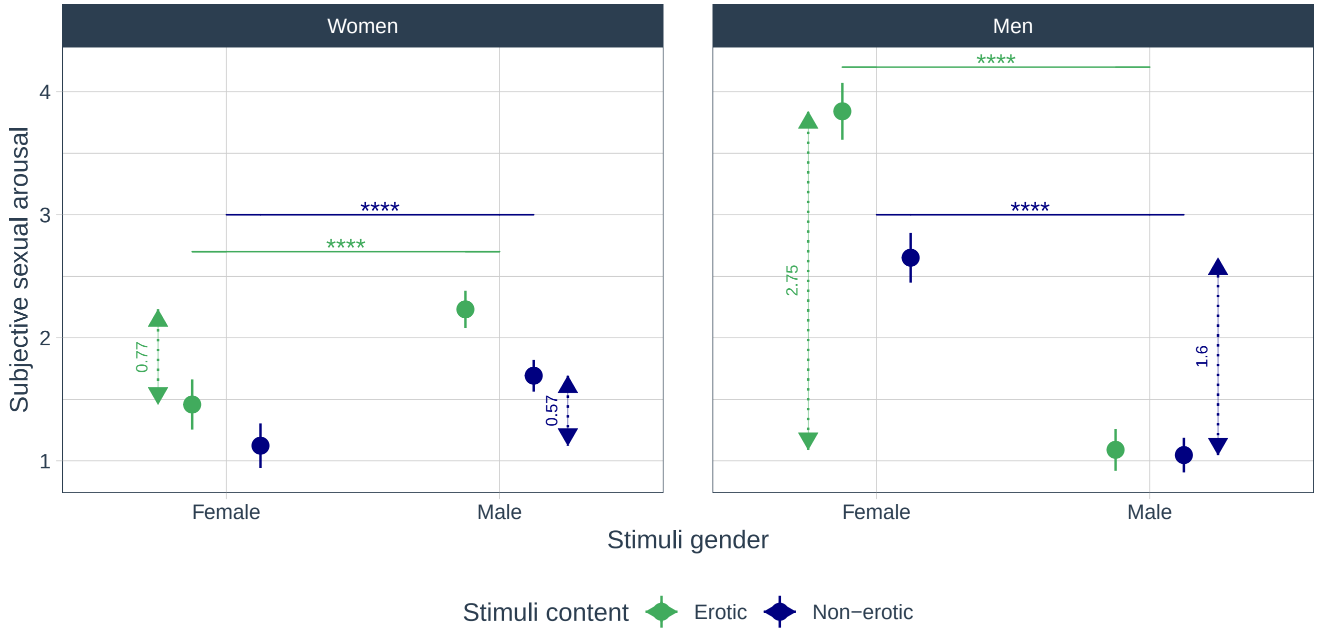

Figure S9. Effects of stimuli content (erotic, non-erotic) on subjective sexual arousal for women's (left panel) and men's (right panel) scores of male and female stimuli (see Table S17; for detailed results, see Table S18). Dots and bars represent estimated marginal means and 95% CI. Vertical lines with arrow heads represent the (absolute) difference in reported subjective sexual arousal for male and femnale stimuli, by stimuli content and gender. In all cases, significant effects are represented with lines and stars: $*p < 0.05$, $**p < 0.01$, $***p < 0.001$, $****p < 0.0001$.

4.3 Hypothesis 2: The association between trait sexual desire (TSD) and subjective sexual arousal (SSA) will vary by TSD dimension, with these associations being gender-specific in men and gender-non-specific in women.

We tested whether the relationship between SSA and TSD varies across the three dimensions of TSD and whether these associations differ between men and women. Specifically, we examined:

- **H2a**: A significant association between solitary TSD and SSA toward erotic stimuli (section 4.3.3)
- **H2b**: A significant association between dyadic TSD toward an attractive person and SSA toward erotic stimuli.
- **H2c**: No significant association between dyadic TSD toward a partner and SSA toward erotic stimuli.

To examine this hypothesis, we modeled the effects of each of the three TSD dimension scores, gender, stimulus gender, and their interactions, on SSA. We included random intercepts for each stimulus, as well as random intercepts and slopes between stimuli gender for each participant.

4.3.1 Modeling Approach

Since SSA is an ordinal variable with seven ordered levels, we fitted the models using three different approaches to ensure the robustness of our results:

1. Cumulative Link Mixed Model (CLMM), using the `clmm` function from the package `ordinal` (Christensen, 2023)
2. Generalized Mixed Model (GLMM) with a Poisson family, using the `glmer` function from the package `lme4` (Bates et al., 2015)
3. Linear mixed model (LMM), using the `lmer` function from the package `lmerTest` (Kuznetsova et al., 2017)

The results across these models were largely consistent, indicating robustness in our findings. For clarity and interpretability, we primarily base our inferences on the LMM, as it provides the most straightforward interpretation and has a wider range of available functions in R for extracting model information.

4.3.2 Data

We created a new dataset by selecting only responses to erotic stimuli, renaming key variables to remove spaces for compatibility with certain functions, and converting relevant variables to factors. Specifically, the Gender and Stimuli gender variables are transformed into factors, and a factor version of Subjective sexual arousal is created for use in the CLMM model.

```
# Filter dataset to include only responses to erotic stimuli
dat_m2 <- dat |>
  filter(`Stimuli content` == "Erotic") |> # Select only erotic stimuli responses
  # Rename variables to remove spaces (improves function compatibility)
  rename(
    Subjective.sexual.arousal = `Subjective sexual arousal`,
    Solitary.TSD = `Solitary sexual desire`,
    Dyadic.TSD.Attractive.Person = `Dyadic sexual desire (Attractive person)`,
    Dyadic.TSD.Partner = `Dyadic sexual desire (Partner)`,
    Stimuli.gender = `Stimuli gender`,
    Stimuli.code = `Stimuli code`
  ) |>
  # Convert categorical variables to factors
  mutate(
    Gender = as.factor(Gender), # Convert Gender to factor
    Stimuli.gender = as.factor(Stimuli.gender), # Convert Stimuli gender to factor
    # Create a factor version of SSA for use in the CLMM model
    Subjective.sexual.arousal.factor = as.factor(Subjective.sexual.arousal)
  )
```

4.3.3 Hypothesis 2a: Solitary TSD

4.3.3.1 Model Robustness: Examining the Effects of Solitary TSD on SSA Across Gender and Stimuli Gender To assess the robustness of our findings, we fitted three different models examining how Solitary TSD predicts SSA, considering variations by gender and stimuli gender:

1. Cumulative Link Mixed Model (CLMM) – `m2a_clmm` (for ordinal outcomes, using a probit link).
2. Generalized Linear Mixed Model (GLMM) with Poisson family – `m2a_poisson` (treating SSA as a count variable).
3. Linear Mixed Model (LMM) – `m2a_lmer` (treating SSA as a continuous variable).

```
# Fit three different models to examine how Dyadic TSD Partner predicts SSA
# Each model considers variations by Gender and Stimuli Gender

# (1) Cumulative Link Mixed Model (CLMM) - Ordinal outcome with probit link
m2a_clmm <- clmm(
  Subjective.sexual.arousal.factor ~ Solitary.TSD * Gender * Stimuli.gender +
    (1 | Stimuli.code) + # Random intercept for Stimuli
    (1 + Stimuli.gender | Participant), # Random intercept & slope for Stimuli gender
  data = dat_m2,
  link = "probit", # Use probit link function for ordinal regression
  control = list(method = "nlminb") # Use 'nlminb' optimizer for better convergence
)

# (2) Generalized Linear Mixed Model (GLMM) - Poisson regression for count data
m2a_poisson <- glmer(
  Subjective.sexual.arousal ~ Solitary.TSD * Gender * Stimuli.gender +
    (1 | Stimuli.code) + # Random intercept for Stimuli
    (1 + Stimuli.gender | Participant), # Random intercept & slope for Stimuli gender
  data = dat_m2,
  family = poisson # Poisson distribution for count data
)
```

```
# (3) Linear Mixed Model (LMM) - Continuous approximation
m2a_lmer <- lmer(
  Subjective.sexual.arousal ~ Solitary.TSD * Gender * Stimuli.gender +
    (1 | Stimuli.code) + # Random intercept for Stimuli
    (1 + Stimuli.gender | Participant), # Random intercept & slope for Stimuli gender
  data = dat_m2,
  control = lmerControl(optimizer = "bobyqa") # Use 'bobyqa' optimizer for stability
)
```

4.3.3.1.1 Table S19. ANOVA-type table of fixed effects (main effects and interactions) across the three fitted models

As shown in the table below, the pattern of significant effects remains consistent across all three models, except for the main effect of gender, which is not significant in the CLMM.

```
# Compare ANOVA-type tables across the three fitted models for Hypothesis 2a
# - CLMM: Cumulative Link Mixed Model
# - GLMM (Poisson): Generalized Linear Mixed Model
# - LMM: Linear Mixed Model
anova.comp(
  CLMMmod = m2a_clmm, # Ordinal regression model (probit link)
  GLMERmod = m2a_poisson, # Poisson regression model (count data)
  LMERmod = m2a_lmer, # Linear mixed model (continuous response)
  hypothesis = "2a" # Label for hypothesis tracking in output
)
```

Table S19. *Comparison of fixed effects across the three models for Hypothesis 2a: CLMM, GLMM (Poisson), and LMM.*

	CLMM			GLMER (Poisson)			LMM		
Effect	*df*	χ^2	*p*	*df*	χ^2	*p*	*df*	*F*	*p*
Solitary TSD	1	27.377	**< 0.0001**	1	24.430	**< 0.0001**	1, 319	17.464	**< 0.0001**
Gender	1	0.015	0.9	1	7.086	**0.0078**	1, 319	8.838	**0.0032**
Stimuli gender	1	43.812	**< 0.0001**	1	31.553	**< 0.0001**	1, 369.21	24.715	**< 0.0001**
Solitary TSD × Gender	1	2.409	0.12	1	2.795	0.09	1, 319	0.852	0.36
Solitary TSD × Stimuli gender	1	0.137	0.71	1	0.321	0.57	1, 319	0.024	0.88
Gender × Stimuli gender	1	181.478	**< 0.0001**	1	127.568	**< 0.0001**	1, 319	74.790	**< 0.0001**
Solitary TSD × Gender × Stimuli gender	1	2.959	0.09	1	0.605	0.44	1, 319	1.778	0.18

Note: For CLMM and GLMER (Poisson) models, results are Analysis of Deviance (Type III Wald chi-square tests), while for LMM, results are from an Analysis of Variance (Type III ANOVA with Satterthwaite's method). Significant effects are in bold.

4.3.3.1.2 Figure S10: Model-based predictions for Hypothesis 2a.

This figure presents model-based predictions of subjective sexual arousal as a function of Solitary TSD, across different stimulus genderes and participant genders. The three subplots correspond to the three statistical models used for analysis: (a) Cumulative Link Mixed Model (CLMM), (b) Generalized Linear Mixed Model (GLMM, Poisson), and (c) Linear Mixed Model (LMM). Shaded areas represent 95% confidence intervals.

```
# CLMM Predictions: Subjective Sexual Arousal ~ Solitary TSD | Gender * Stimuli Gender
p_m2a_clmm <- emmeans(m2a_clmm, ~ Solitary.TSD | Gender * Stimuli.gender,
  at = list(Solitary.TSD = seq(0, 31, length.out = 100)), # Predict over range of TSD
  mode = "mean.class" # Compute predicted mean response categories
) |>
  as.data.frame() |> # Convert predictions to dataframe
  ggplot(aes(
    x = Solitary.TSD, y = mean.class,
    color = Stimuli.gender, fill = Stimuli.gender
  )) +
  geom_line(size = 1) + # Add prediction lines
  geom_ribbon(aes(ymin = asymp.LCL, ymax = asymp.UCL, fill = Stimuli.gender),
```

```
    alpha = 0.2, color = NA
  ) + # Add confidence interval shading
  scale_color_manual(values = color.StimuliSex) +
  scale_fill_manual(values = color.StimuliSex) +
  facet_wrap(~Gender, ncol = 1) + # Separate plots by Gender
  labs(
    y = "Predicted SSA", x = "Solitary TSD", title = "CLMM",
    color = "Stimuli Gender", fill = "Stimuli Gender"
  ) +
  theme_tq() +
  theme(legend.position = "bottom") +
  ylim(c(0.3, 5.3)) # Set Y-axis limits

# Poisson GLMM Predictions
p_m2a_poisson <- emmeans(m2a_poisson, ~ Solitary.TSD | Gender * Stimuli.gender,
  at = list(Solitary.TSD = seq(0, 31, length.out = 100)),
  type = "response" # Compute response-scale predictions
) |>
  as.data.frame() |>
  ggplot(aes(
    x = Solitary.TSD, y = rate,
    color = Stimuli.gender, fill = Stimuli.gender
  )) +
  geom_line(size = 1) +
  geom_ribbon(aes(ymin = asymp.LCL, ymax = asymp.UCL, fill = Stimuli.gender),
    alpha = 0.2, color = NA
  ) +
  scale_color_manual(values = color.StimuliSex) +
  scale_fill_manual(values = color.StimuliSex) +
  facet_wrap(~Gender, ncol = 1) +
  labs(
    y = "", x = "Solitary TSD", title = "GLMER (Poisson)",
    color = "Stimuli Gender", fill = "Stimuli Gender"
  ) +
  theme_tq() +
  theme(legend.position = "bottom") +
  ylim(c(0.3, 5.3))

# LMM Predictions
p_m2a_lmer <- emmeans(m2a_lmer, ~ Solitary.TSD | Gender * Stimuli.gender,
  at = list(Solitary.TSD = seq(0, 31, length.out = 100)),
  type = "response"
) |>
  as.data.frame() |>
  ggplot(aes(
    x = Solitary.TSD, y = emmean,
    color = Stimuli.gender, fill = Stimuli.gender
  )) +
  geom_line(size = 1) +
  geom_ribbon(aes(ymin = asymp.LCL, ymax = asymp.UCL, fill = Stimuli.gender),
    alpha = 0.2, color = NA
  ) +
  scale_color_manual(values = color.StimuliSex) +
  scale_fill_manual(values = color.StimuliSex) +
  facet_wrap(~Gender, ncol = 1) +
  labs(
```

```
    y = "", x = "Solitary TSD", title = "LMM",
    color = "Stimuli Gender", fill = "Stimuli Gender"
  ) +
  theme_tq() +
  theme(legend.position = "bottom") +
  ylim(c(0.3, 5.3))

# Arrange all plots into a single figure
p_robu_m2a <- ggarrange(p_m2a_clmm, p_m2a_poisson, p_m2a_lmer,
  common.legend = TRUE, # Share legend across plots
  labels = "auto", # Automatically label subfigures (a, b, c)
  legend = "bottom",
  nrow = 1 # Arrange plots in a single row
)

# Display the combined figure
p_robu_m2a
```

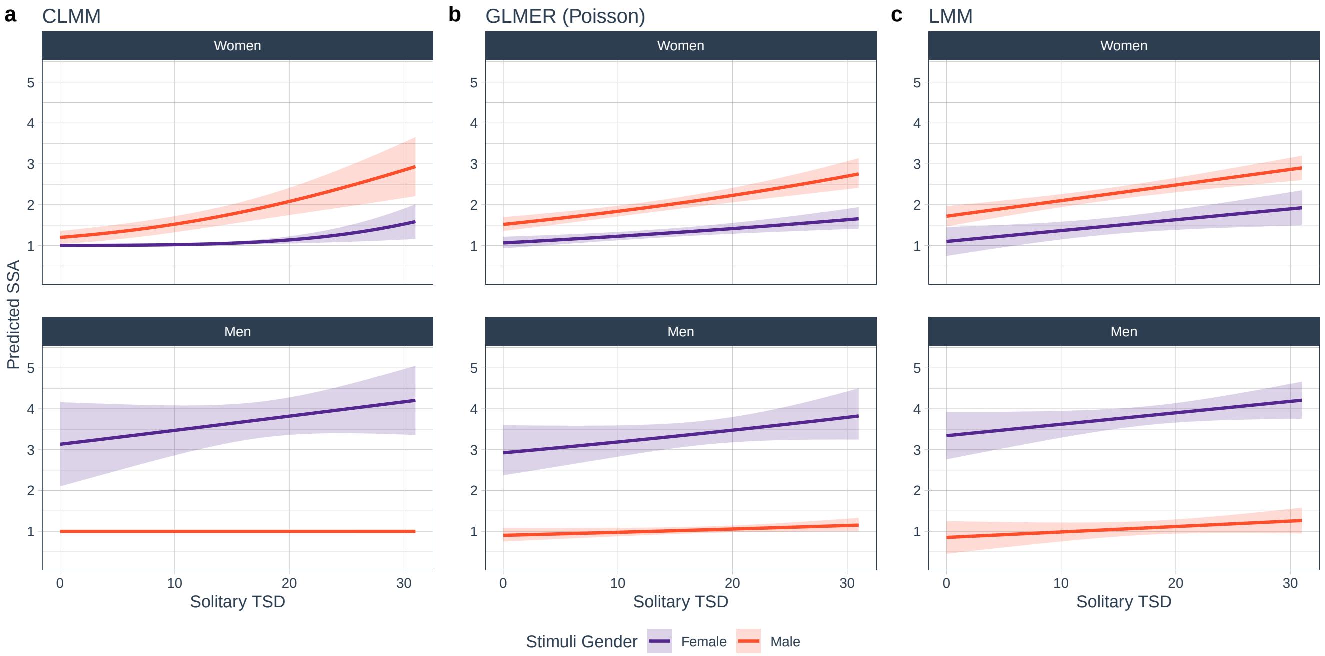

Figure S10. Predicted subjective sexual arousal as a function of Solitary TSD, modeled using three statistical approaches: **(a)** Cumulative Link Mixed Model (CLMM); **(b)** Generalized Linear Mixed Model (GLMM) with a Poisson family; **(c)** Linear Mixed Model (LMM). Lines represent predicted values, and shaded areas indicate 95% confidence intervals. The models include participant gender and stimulus gender as key factors.

4.3.3.2 Final Model: Effects of Solitary TSD on SSA Across Gender and Stimuli Gender Given the apparent robustness of most results across models (CLMM, GLMER and LMM; Table S19, Fig. S10), we test the predictions of the hypothesis from the LMM (`m2a_lmer`).

4.3.3.2.1 Table S20. ANOVA-type table for the interaction between `Relationship type`, and `Gender` This tables summarizes the results of the model.

```
# Generate ANOVA-type table for the final LMM model
# This summarizes the effects of Solitary TSD on SSA across Gender and Stimuli Gender
anova.sig.lmer(
  model = m2a_lmer, # Use LMM as the final model
```

```
  custom_caption = "Effects of Solitary TSD on SSA Across Gender and Stimuli Gender"
)
```

Table S20. *Effects of Solitary TSD on SSA Across Gender and Stimuli Gender*

Effect	*df*	*F*	*p*	ϵ_p^2
Solitary TSD	1, 319	17.46	**< 0.0001**	0.0489
Gender	1, 319	8.84	**0.0032**	0.0239
Stimuli gender	1, 369.21	24.71	**< 0.0001**	0.06
Solitary TSD × Gender	1, 319	0.85	0.36	< 0.0001
Solitary TSD × Stimuli gender	1, 319	0.02	0.88	< 0.0001
Gender × Stimuli gender	1, 319	74.79	**< 0.0001**	0.19
Solitary TSD × Gender × Stimuli gender	1, 319	1.78	0.18	0.0024

Note: Results are Type III ANOVA. $R^2_{conditional} = 0.745$, $R^2_{marginal} = 0.335$. As effect size, we report partial epsilon squared (ϵ_p^2), a less biased estimate than η^2 (see Albers and Lakens, 2018). Significant effects are in bold.

4.3.3.2.2 ***Post-hoc* tests** To test the hypothesis, which predicted that there would be different relationship between SSA and solitary TSD, and that this association differ between men and women depending on the gender of stimuli, we used simple slope analysis.

```
# Perform simple slopes analysis for Solitary TSD on SSA
# Moderators: Stimuli Gender & Gender
slop.m2a_lmer <- sim_slopes(m2a_lmer,
  pred = Solitary.TSD, # Predictor: Solitary TSD
  modx = Stimuli.gender, # First moderator: Stimuli gender
  mod2 = Gender, # Second moderator: Gender
  confint = TRUE # Compute confidence intervals
)

# Extract slopes separately for Women and Men
slop.m2a_lmer.tab <- bind_rows(
  slop.m2a_lmer$slopes[[1]] |> mutate(Gender = "Women"),
  slop.m2a_lmer$slopes[[2]] |> mutate(Gender = "Men")
) |>
  # Recode Gender labels
  mutate(Gender = recode_factor(Gender, Femenino = "Women", Masculino = "Men")) |>
  # Select relevant columns and convert numeric values
  select(8, 1:2, 4:7) |>
  mutate(across(3:7, as.numeric)) |>
  mutate(across(3:6, round, 2)) |> # Round confidence intervals
  mutate(sig = pval.stars(p)) |> # Convert p-values to significance stars
  rename("Stimuli.gender" = "Value of Stimuli.gender") |>
  rename(Coefficient = Est.)

# Format the table output
slop.m2a_lmer.tab[, -c(1, 8)] |>
  mutate(p = pval.lev(p)) |> # Format p-values
  kable(
    booktabs = TRUE, align = c("l", rep("c", 5)),
    linesep = "",
    caption = "Slope for Solitary TSD on Subjective Sexual Arousal by Stimuli Gender and Gender",
    col.names = c("Stimuli gender", "$B$", "$2.5\\% CI$", "$97.5\\% CI$", "$t$", "$p$"),
    escape = FALSE
  ) |>
```

```
  kable_styling(latex_options = c("HOLD_position")) |>
  # Add row grouping for gender
  pack_rows("Gender: Women", start_row = 1, end_row = 2,
            bold = FALSE, background = "lightgray") |>
  pack_rows("Gender: Men", start_row = 3, end_row = 4,
            bold = FALSE, background = "lightgray") |>
  # Add footnote explaining coefficients
  footnote(
    general = "$B$ are unstandardized coefficients.
    No intercept is reported as continuous predictors were centered
    and are dependent on this specific sample.",
    threeparttable = TRUE, footnote_as_chunk = TRUE, escape = FALSE
  )
```

Table S21. *Slope for Solitary TSD on Subjective Sexual Arousal by Stimuli Gender and Gender*

Stimuli gender	B	2.5%CI	97.5%CI	t	p
Gender: Women					
Female	0.03	0.01	0.05	2.42	**0.0162**
Male	0.04	0.02	0.05	5.07	$<$ **0.0001**
Gender: Men					
Female	0.03	0.00	0.06	1.84	0.07
Male	0.01	-0.01	0.03	1.28	0.2

Note: B are unstandardized coefficients. No intercept is reported as continuous predictors were centered and are dependent on this specific sample.

4.3.3.3 Figure S11. Subjective sexual arousal to erotic stimuli: Main effects and interactions This figure summarizes the results of hypothesis 2a.

```
# Modify LMM plot to visualize main effects & interactions
p_m2a.fin <- p_m2a_lmer +
  labs(
    title = "", # Remove title
    y = "Predicted Subjective Sexual Arousal"
  ) + # Update y-axis label
  facet_wrap(~Gender, ncol = 2) + # Separate plots by Gender
  # Add text labels for regression slopes (B, CI, p-values)
  geom_text(
    data = slop.m2a_lmer.tab |>
      mutate(Solitary.TSD = 2), # Set label position
    mapping = aes(
      x = min(Solitary.TSD), y = Inf,
      label = paste(
        "B = ", Coefficient,
        ", IC 95%[", `2.5%`, ", ", `97.5%`, "], p",
        ifelse(grepl("<", pe2.lev(p)), pe2.lev(p),
          paste0(" = ", pe2.lev(p))
        ),
        ifelse(is.na(sig), "", sig)
      ),
      vjust = 2 + as.numeric(as.factor(Stimuli.gender)) * 2 # Stack labels
    ),
    hjust = -0.1, # Align text to the left
    show.legend = FALSE
```

```
  ) + # Hide legend for text
  theme(legend.position = "bottom") # Move legend to bottom

# Display the final figure
p_m2a.fin
```

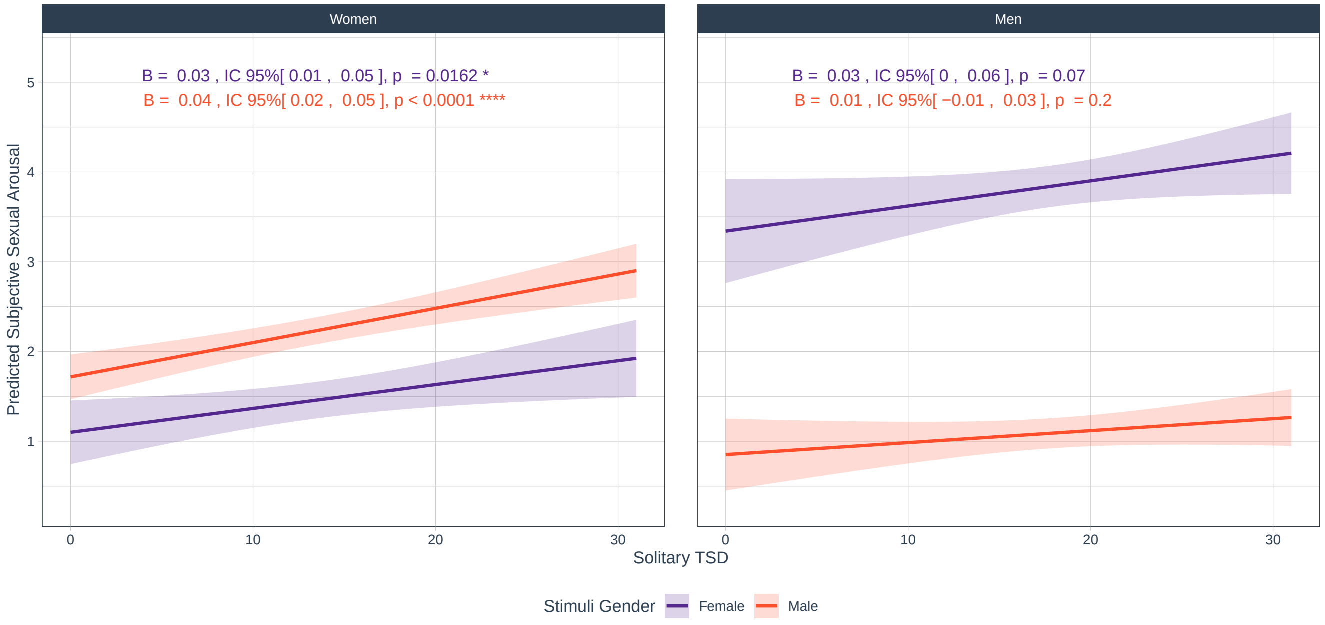

Figure S11. Predicted subjective sexual arousal as a function of Solitary TSD, modeled using aLinear Mixed Model (LMM). Lines represent predicted values, and shaded areas indicate 95% confidence intervals. The model include participant gender and stimuli gender as key factors.

4.3.4 Hypothesis 2b: Dyadic TSD Attractive Person

4.3.4.1 Model Robustness: Examining the Effects of Dyadic TSD Attractive Person on SSA Across Gender and Stimuli Gender To assess the robustness of our findings, we fitted three different models examining how Dyadic TSD Attractive Person predicts SSA, considering variations by gender and stimuli gender:

1. Cumulative Link Mixed Model (CLMM) – `m2b_clmm` (for ordinal outcomes, using a probit link).
2. Generalized Linear Mixed Model (GLMM) with Poisson family – `m2b_poisson` (treating SSA as a count variable).
3. Linear Mixed Model (LMM) – `m2b_lmer` (treating SSA as a continuous variable).

```
# Fit three different models to examine how Dyadic TSD Partner predicts SSA
# Each model considers variations by Gender and Stimuli Gender

# (1) Cumulative Link Mixed Model (CLMM) - Ordinal outcome with probit link
m2b_clmm <- clmm(
  Subjective.sexual.arousal.factor ~ Dyadic.TSD.Attractive.Person * Gender * Stimuli.gender +
    (1 | Stimuli.code) + # Random intercept for Stimuli
    (1 + Stimuli.gender | Participant), # Random intercept & slope for Stimuli gender
  data = dat_m2,
  link = "probit", # Use probit link function
  control = list(method = "nlminb") # Use 'nlminb' optimizer for better convergence
)

# (2) Generalized Linear Mixed Model (GLMM) - Poisson regression for count data
m2b_poisson <- glmer(
```

```
  Subjective.sexual.arousal ~ Dyadic.TSD.Attractive.Person * Gender * Stimuli.gender +
    (1 | Stimuli.code) + # Random intercept for Stimuli
    (1 + Stimuli.gender | Participant), # Random intercept & slope for Stimuli gender
  data = dat_m2,
  family = poisson # Use Poisson distribution for count data
)

# (3) Linear Mixed Model (LMM) - Continuous approximation
m2b_lmer <- lmer(
  Subjective.sexual.arousal ~ Dyadic.TSD.Attractive.Person * Gender * Stimuli.gender +
    (1 | Stimuli.code) + # Random intercept for Stimuli
    (1 + Stimuli.gender | Participant), # Random intercept & slope for Stimuli gender
  data = dat_m2,
  control = lmerControl(optimizer = "bobyqa") # Use 'bobyqa' optimizer for stability
)
```

4.3.4.1.1 Table S22. ANOVA-type table of fixed effects (main effects and interactions) across the three fitted models As shown in the table below, the pattern of significant effects remains consistent across all three models, except for the main effect of gender, which is not significant in the CLMM.

```
# Compare fixed effects across the three models for Hypothesis 2b
# CLMM: Cumulative Link Mixed Model
# GLMER: Generalized Linear Mixed Model (Poisson)
# LMER: Linear Mixed Model
anova.comp(
  CLMMmod = m2b_clmm, # CLMM model
  GLMERmod = m2b_poisson, # GLMER model
  LMERmod = m2b_lmer, # LMER model
  hypothesis = "2b" # Label hypothesis for output
)
```

Table S22. *Comparison of fixed effects across the three models for Hypothesis 2b: CLMM, GLMM (Poisson), and LMM.*

	CLMM			GLMER (Poisson)			LMM		
Effect	*df*	χ^2	*p*	*df*	χ^2	*p*	*df*	*F*	*p*
Dyadic TSD Attractive Person	1	36.545	**< 0.0001**	1	45.711	**< 0.0001**	1, 319	48.490	**< 0.0001**
Gender	1	0.031	0.86	1	3.059	0.08	1, 319	1.446	0.23
Stimuli gender	1	18.293	**< 0.0001**	1	7.365	**0.0067**	1, 373.93	2.689	0.1
Dyadic TSD Attractive Person × Gender	1	3.774	0.05	1	0.940	0.33	1, 319	0.530	0.47
Dyadic TSD Attractive Person × Stimuli gender	1	7.654	**0.0057**	1	7.507	**0.0061**	1, 319	15.428	**< 0.001**
Gender × Stimuli gender	1	124.186	**< 0.0001**	1	67.054	**< 0.0001**	1, 319	27.444	**< 0.0001**
Dyadic TSD Attractive Person × Gender × Stimuli gender	1	3.833	0.05	1	20.127	**< 0.0001**	1, 319	29.689	**< 0.0001**

Note: For CLMM and GLMER (Poisson) models, results are Analysis of Deviance (Type III Wald chi-square tests), while for LMM, results are from an Analysis of Variance (Type III ANOVA with Satterthwaite's method). Significant effects are in bold.

4.3.4.1.2 Figure S12: Model-based predictions for Hypothesis 2b. This figure presents model-based predictions of subjective sexual arousal as a function of Dyadic TSD Attractive Person, across different stimulus genderes and participant genders. The three subplots correspond to the three statistical models used for analysis: (a) Cumulative Link Mixed Model (CLMM), (b) Generalized Linear Mixed Model (GLMM, Poisson), and (c) Linear Mixed Model (LMM). Shaded areas represent 95% confidence intervals.

```
# CLMM Predictions
p_m2b_clmm <- emmeans(m2b_clmm, ~ Dyadic.TSD.Attractive.Person | Gender * Stimuli.gender,
  at = list(Dyadic.TSD.Attractive.Person = seq(0, 31, length.out = 100)),
  mode = "mean.class" # Compute predicted mean response categories
) |>
  as.data.frame() |> # Convert to dataframe for ggplot
```

```
  ggplot(aes(
    x = Dyadic.TSD.Attractive.Person, y = mean.class,
    color = Stimuli.gender, fill = Stimuli.gender
  )) +
  geom_line(size = 1) + # Add predicted response line
  geom_ribbon(aes(ymin = asymp.LCL, ymax = asymp.UCL, fill = Stimuli.gender),
    alpha = 0.2, color = NA
  ) + # Add confidence interval as shaded ribbon
  scale_color_manual(values = color.StimuliSex) +
  scale_fill_manual(values = color.StimuliSex) +
  facet_wrap(~Gender, ncol = 1) + # Create separate plots for each gender
  labs(
    y = "Predicted Subjective Sexual Arousal",
    x = "Dyadic TSD Attractive Person",
    title = "CLMM", color = "Stimuli Gender", fill = "Stimuli Gender"
  ) +
  theme_tq() +
  theme(legend.position = "bottom") +
  ylim(c(0.3, 6.5)) # Set Y-axis limits

# Poisson GLMM Predictions
p_m2b_poisson <- emmeans(m2b_poisson, ~ Dyadic.TSD.Attractive.Person | Gender * Stimuli.gender,
  at = list(Dyadic.TSD.Attractive.Person = seq(0, 31, length.out = 100)),
  type = "response" # Compute response-scale predictions
) |>
  as.data.frame() |>
  ggplot(aes(
    x = Dyadic.TSD.Attractive.Person, y = rate,
    color = Stimuli.gender, fill = Stimuli.gender
  )) +
  geom_line(size = 1) +
  geom_ribbon(aes(ymin = asymp.LCL, ymax = asymp.UCL, fill = Stimuli.gender),
    alpha = 0.2, color = NA
  ) +
  scale_color_manual(values = color.StimuliSex) +
  scale_fill_manual(values = color.StimuliSex) +
  facet_wrap(~Gender, ncol = 1) +
  labs(
    y = "", x = "Dyadic TSD Attractive Person",
    title = "GLMER (Poisson)", color = "Stimuli Gender", fill = "Stimuli Gender"
  ) +
  theme_tq() +
  theme(legend.position = "bottom") +
  ylim(c(0.3, 6.5))

# LMM Predictions
p_m2b_lmer <- emmeans(m2b_lmer, ~ Dyadic.TSD.Attractive.Person | Gender * Stimuli.gender,
  at = list(Dyadic.TSD.Attractive.Person = seq(0, 31, length.out = 100)),
  type = "response"
) |>
  as.data.frame() |>
  ggplot(aes(
    x = Dyadic.TSD.Attractive.Person, y = emmean,
    color = Stimuli.gender, fill = Stimuli.gender
  )) +
  geom_line(size = 1) +
```

```
  geom_ribbon(aes(ymin = asymp.LCL, ymax = asymp.UCL, fill = Stimuli.gender),
    alpha = 0.2, color = NA
  ) +
  scale_color_manual(values = color.StimuliSex) +
  scale_fill_manual(values = color.StimuliSex) +
  facet_wrap(~Gender, ncol = 1) +
  labs(
    y = "", x = "Dyadic TSD Attractive Person",
    title = "LMM", color = "Stimuli Gender", fill = "Stimuli Gender"
  ) +
  theme_tq() +
  theme(legend.position = "bottom") +
  ylim(c(0.3, 6.5))

# Arrange Plots into a Single Figure
p_robu_m2b <- ggarrange(
  p_m2b_clmm, p_m2b_poisson, p_m2b_lmer, # Combine plots side by side
  common.legend = TRUE, # Share legend across plots
  labels = "auto", # Automatically label subfigures (a, b, c)
  legend = "bottom",
  nrow = 1 # Arrange in a single row
)

# Display the combined figure
p_robu_m2b
```

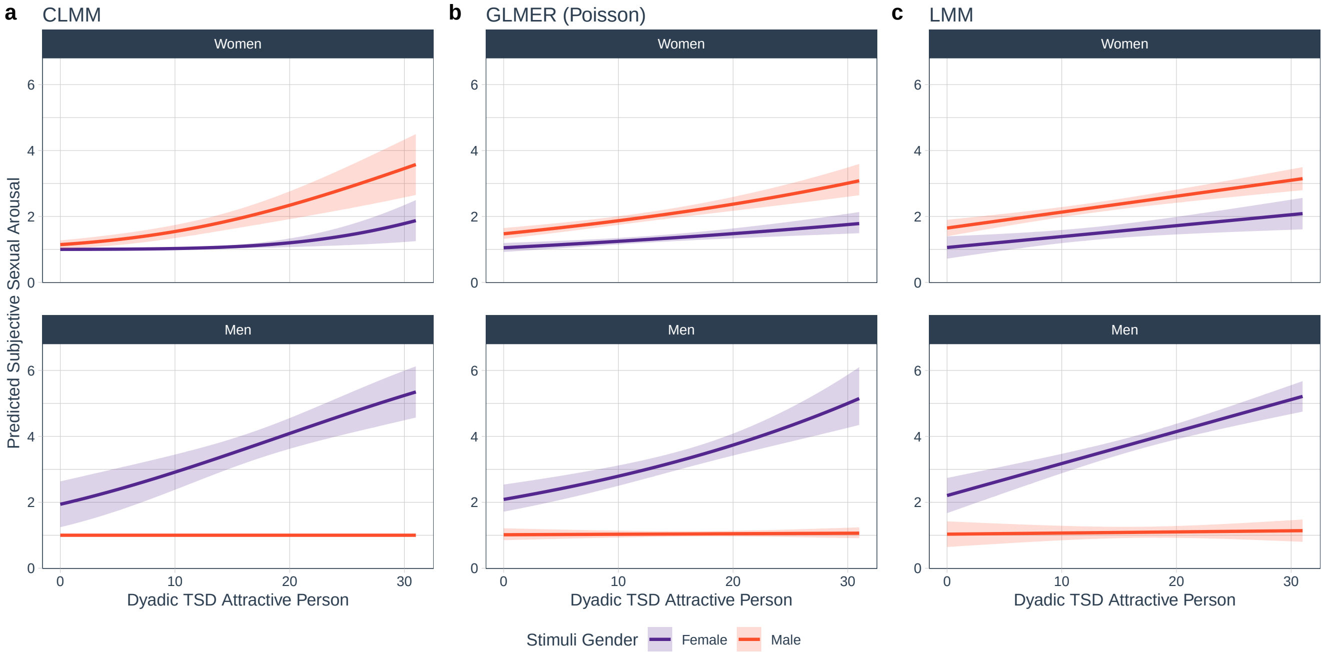

Figure S12. Predicted subjective sexual arousal as a function of Dyadic TSD Attractive Person, modeled using three statistical approaches: **(a)** Cumulative Link Mixed Model (CLMM); **(b)** Generalized Linear Mixed Model (GLMM) with a Poisson family; **(c)** Linear Mixed Model (LMM). Lines represent predicted values, and shaded areas indicate 95% confidence intervals. The models include participant gender and stimulus gender as key factors.

4.3.4.2 Final Model: Effects of Dyadic TSD Attractive Person on SSA Across Gender and Stimuli Gender

Given the apparent robustness of most results across models (CLMM, GLMER and LMM; Table S22, Fig. S12), we test the predictions of the hypothesis from the LMM (`m2b_lmer`).

4.3.4.2.1 Table S23. ANOVA-type table for the interaction between `Relationship type`, and `Gender`

This tables summarizes the results of the model.

```
# Generate ANOVA-type table for the final LMM model
# Summarizes the effects of Dyadic TSD Attractive Person on SSA across Gender and Stimuli Gender
anova.sig.lmer(
  model = m2b_lmer, # Use LMM as the final model
  custom_caption = "Effects of Dyadic TSD Attractive Person on SSA
  Across Gender and Stimuli Gender"
)
```

Table S23. *Effects of Dyadic TSD Attractive Person on SSA Across Gender and Stimuli Gender*

Effect	*df*	*F*	*p*	ϵ_p^2
Dyadic TSD Attractive Person	1, 319	48.49	**< 0.0001**	0.13
Gender	1, 319	1.45	0.23	0.0014
Stimuli gender	1, 373.93	2.69	0.1	0.0045
Dyadic TSD Attractive Person × Gender	1, 319	0.53	0.47	< 0.0001
Dyadic TSD Attractive Person × Stimuli gender	1, 319	15.43	**< 0.001**	0.0431
Gender × Stimuli gender	1, 319	27.44	**< 0.0001**	0.08
Dyadic TSD Attractive Person × Gender × Stimuli gender	1, 319	29.69	**< 0.0001**	0.08

Note: Results are Type III ANOVA. $R^2_{conditional} = 0.745$, $R^2_{marginal} = 0.367$. As effect size, we report partial epsilon squared (ϵ_p^2), a less biased estimate than η^2 (see Albers and Lakens, 2018). Significant effects are in bold.

4.3.4.2.2 *Post-hoc* tests

To test the hypothesis, which predicted that there would be different relationship between SSA and Dyadic TSD Attractive Person, and that this association differ between men and women depending on the gender of stimuli, we used simple slope analysis.

```
# Perform simple slope analysis for Dyadic TSD Attractive Person on SSA
# This examines differences by stimuli gender and participant gender
slop.m2b_lmer <- sim_slopes(
  m2b_lmer,
  pred = Dyadic.TSD.Attractive.Person, # Predictor: Dyadic TSD Attractive Person
  modx = Stimuli.gender, # Moderator: Stimuli gender
  mod2 = Gender, # Second moderator: Gender
  confint = TRUE # Compute confidence intervals
)

# Format the slope results into a structured table
slop.m2b_lmer.tab <- bind_rows(
  slop.m2b_lmer$slopes[[1]] |> mutate(Gender = "Women"),
  slop.m2b_lmer$slopes[[2]] |> mutate(Gender = "Men")
) |>
  mutate(Gender = recode_factor(Gender, Femenino = "Women", Masculino = "Men")) |>
  select(8, 1:2, 4:7) |>
  mutate(across(3:7, as.numeric)) |>
  mutate(across(3:6, round, 2)) |>
  mutate(sig = pval.stars(p)) |>
  rename("Stimuli.gender" = "Value of Stimuli.gender", Coefficient = Est.)

# Generate formatted table for slopes of Dyadic TSD Attractive Person on SSA
slop.m2b_lmer.tab[, -c(1, 8)] |>
  mutate(p = pval.lev(p)) |>
  kable(
    booktabs = TRUE,
    align = c("l", rep("c", 5)),
```

```
    caption = "Slope for Dyadic TSD Attractive Person on SSA by stimuli gender and gender",
    linesep = "",
    col.names = c("Stimuli gender", "$B$", "$2.5\\% CI$", "$97.5\\% CI$", "$t$", "$p$"),
    escape = FALSE
  ) |>
  kable_styling(latex_options = c("HOLD_position")) |>
  pack_rows(
    group_label = "Gender: Women", start_row = 1, end_row = 2,
    bold = FALSE, background = "lightgray"
  ) |>
  pack_rows(
    group_label = "Gender: Men", start_row = 3, end_row = 4,
    bold = FALSE, background = "lightgray"
  ) |>
  footnote(
    general = "$B$ represents unstandardized coefficients. No intercept is reported as
    continuous predictors were centered and are dependent on this specific sample.",
    threeparttable = TRUE,
    footnote_as_chunk = TRUE,
    escape = FALSE
  )
```

Table S24. *Slope for Dyadic TSD Attractive Person on SSA by stimuli gender and gender*

Stimuli gender	B	$2.5\% CI$	$97.5\% CI$	t	p
Gender: Women					
Female	0.03	0.01	0.06	2.82	**0.0051**
Male	0.05	0.03	0.06	5.70	$<$ **0.0001**
Gender: Men					
Female	0.10	0.07	0.13	6.58	$<$ **0.0001**
Male	0.00	-0.02	0.02	0.32	0.75

Note: B represents unstandardized coefficients. No intercept is reported as continuous predictors were centered and are dependent on this specific sample.

4.3.4.3 Figure S13. Subjective sexual arousal to erotic stimuli: Main effects and interactions This figure summarizes the results of hypothesis 2b.

```
# Generate the final figure for Hypothesis 2b
# Visualizes the effects of Dyadic TSD Attractive Person on SSA across Gender and Stimuli Gender
p_m2b.fin <- p_m2b_lmer +
  labs(
    title = "",
    y = "Predicted Subjective Sexual Arousal"
  ) +
  facet_wrap(~Gender, ncol = 2) + # Create separate plots for each gender
  # Add text labels with slope values, adjusting placement
  geom_text(
    data = slop.m2b_lmer.tab |> mutate(Dyadic.TSD.Attractive.Person = 2),
    mapping = aes(
      x = min(Dyadic.TSD.Attractive.Person), y = Inf,
      label = paste(
        "B = ", Coefficient, ", IC 95%[", `2.5%`, ", ", `97.5%`, "], p",
        ifelse(grepl("<", pe2.lev(p)), pe2.lev(p), paste0(" = ", pe2.lev(p))),
        ifelse(is.na(sig), "", sig)
```

```
      ),
      vjust = 2 + as.numeric(as.factor(Stimuli.gender)) * 2 # Adjust vertical positioning
    ),
    hjust = -0.1, # Align text to the left
    show.legend = FALSE # Remove legends from text annotations
  ) +
  theme(legend.position = "bottom") # Move legend to the bottom

# Display the final figure
p_m2b.fin
```

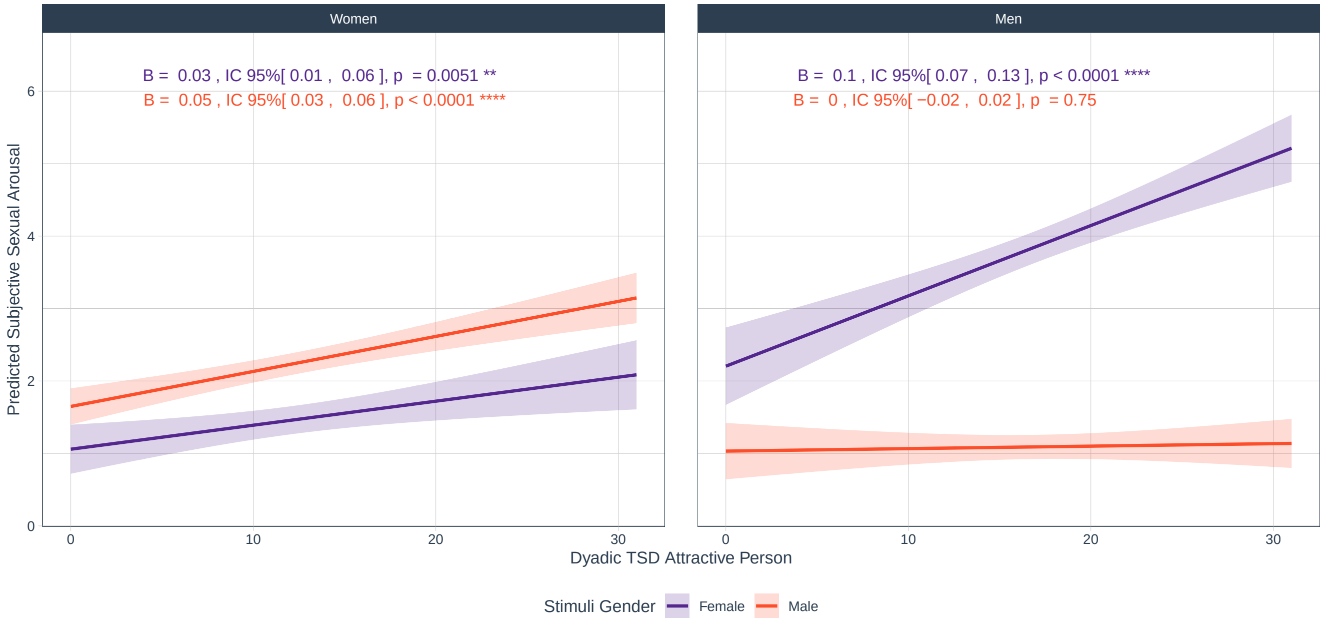

Figure S13. Predicted subjective sexual arousal as a function of Dyadic TSD Partner, modeled using aLinear Mixed Model (LMM). Lines represent predicted values, and shaded areas indicate 95% confidence intervals. The model include participant gender and stimuli gender as key factors.

4.3.5 Hypothesis 2c: Dyadic TSD Partner

4.3.5.1 Model Robustness: Examining the Effects of Dyadic TSD Partner on SSA Across Gender and Stimuli Gender To assess the robustness of our findings, we fitted three different models examining how Dyadic TSD Partner predicts SSA, considering variations by gender and stimuli gender:

1. Cumulative Link Mixed Model (CLMM) – `m2c_clmm` (for ordinal outcomes, using a probit link).
2. Generalized Linear Mixed Model (GLMM) with Poisson family – `m2c_poisson` (treating SSA as a count variable).
3. Linear Mixed Model (LMM) – `m2c_lmer` (treating SSA as a continuous variable).

```
# Fit three different models to examine how Dyadic TSD Partner predicts SSA
# Each model considers variations by Gender and Stimuli Gender

# (1) Cumulative Link Mixed Model (CLMM) - Ordinal outcome with probit link
m2c_clmm <- clmm(
  Subjective.sexual.arousal.factor ~ Dyadic.TSD.Partner * Gender * Stimuli.gender +
    (1 | Stimuli.code) + # Random intercept for Stimuli
    (1 + Stimuli.gender | Participant), # Random intercept & slope for Stimuli gender
  data = dat_m2,
  link = "probit",
```

```
  control = list(method = "nlminb") # Use 'nlminb' optimizer for better convergence
)

# (2) Generalized Linear Mixed Model (GLMM) - Poisson regression for count data
m2c_poisson <- glmer(
  Subjective.sexual.arousal ~ Dyadic.TSD.Partner * Gender * Stimuli.gender +
    (1 | Stimuli.code) + # Random intercept for Stimuli
    (1 + Stimuli.gender | Participant), # Random intercept & slope for Stimuli gender
  data = dat_m2,
  family = poisson # Poisson distribution for count data
)

# (3) Linear Mixed Model (LMM) - Continuous approximation
m2c_lmer <- lmer(
  Subjective.sexual.arousal ~ Dyadic.TSD.Partner * Gender * Stimuli.gender +
    (1 | Stimuli.code) + # Random intercept for Stimuli
    (1 + Stimuli.gender | Participant), # Random intercept & slope for Stimuli gender
  data = dat_m2,
  control = lmerControl(optimizer = "bobyqa") # Use 'bobyqa' optimizer for stability
)
```

4.3.5.1.1 Table S25. ANOVA-type table of fixed effects (main effects and interactions) across the three fitted models As shown in the table below, the pattern of significant effects remains consistent across all three models, except for the main effect of gender, which is not significant in the CLMM.

```
# Compare ANOVA-type tables across the three fitted models
# This evaluates fixed effects (main effects & interactions) for Dyadic TSD Partner on SSA
anova.comp(
  CLMMmod = m2c_clmm, # Cumulative Link Mixed Model
  GLMERmod = m2c_poisson, # Generalized Linear Mixed Model (Poisson)
  LMERmod = m2c_lmer, # Linear Mixed Model
  hypothesis = "2c" # Specifies hypothesis 2c
)
```

Table S25. *Comparison of fixed effects across the three models for Hypothesis 2c: CLMM, GLMM (Poisson), and LMM.*

	CLMM			GLMER (Poisson)			LMM		
Effect	*df*	χ^2	*p*	*df*	χ^2	*p*	*df*	*F*	*p*
Dyadic TSD Partner	1	0.642	0.42	1	5.150	**0.0232**	1, 316	6.589	**0.0107**
Gender	1	0.743	0.39	1	0.078	0.78	1, 316	0.034	0.85
Stimuli gender	1	10.881	$<$ **0.001**	1	2.688	0.1	1, 344.42	0.991	0.32
Dyadic TSD Partner × Gender	1	0.310	0.58	1	2.111	0.15	1, 316	3.967	**0.0472**
Dyadic TSD Partner × Stimuli gender	1	2.366	0.12	1	5.423	**0.0199**	1, 316	8.458	**0.0039**
Gender × Stimuli gender	1	61.739	$<$ **0.0001**	1	45.783	$<$ **0.0001**	1, 316	20.549	$<$ **0.0001**
Dyadic TSD Partner × Gender × Stimuli gender	1	1.254	0.26	1	2.461	0.12	1, 316	5.700	**0.0176**

Note: For CLMM and GLMER (Poisson) models, results are Analysis of Deviance (Type III Wald chi-square tests), while for LMM, results are from an Analysis of Variance (Type III ANOVA with Satterthwaite's method). Significant effects are in bold.

4.3.5.1.2 Figure S14: Model-based predictions for Hypothesis 2c. This figure presents model-based predictions of subjective sexual arousal as a function of Dyadic TSD Partner, across different stimulus genderes and participant genders. The three subplots correspond to the three statistical models used for analysis: (a) Cumulative Link Mixed Model (CLMM), (b) Generalized Linear Mixed Model (GLMM, Poisson), and (c) Linear Mixed Model (LMM). Shaded areas represent 95% confidence intervals.

```
# Generate model-based predictions for Dyadic TSD Partner on SSA
# across different statistical models (CLMM, GLMER, LMM)
```

```
# CLMM Predictions
p_m2c_clmm <- emmeans(m2c_clmm, ~ Dyadic.TSD.Partner | Gender * Stimuli.gender,
  at = list(Dyadic.TSD.Partner = seq(0, 31, length.out = 100)),
  mode = "mean.class"
) |>
  as.data.frame() |>
  ggplot(aes(
    x = Dyadic.TSD.Partner, y = mean.class,
    color = Stimuli.gender, fill = Stimuli.gender
  )) +
  geom_line(size = 1) +
  geom_ribbon(aes(ymin = asymp.LCL, ymax = asymp.UCL), alpha = 0.2, color = NA) +
  scale_color_manual(values = color.StimuliSex) +
  scale_fill_manual(values = color.StimuliSex) +
  facet_wrap(~Gender, ncol = 1) +
  labs(
    y = "Predicted Subjective Sexual Arousal",
    x = "Dyadic TSD Partner",
    title = "CLMM",
    color = "Stimuli Gender", fill = "Stimuli Gender"
  ) +
  theme_tq() +
  theme(legend.position = "bottom") +
  ylim(c(0.3, 5.3))

# Poisson GLMM Predictions
p_m2c_poisson <- emmeans(m2c_poisson, ~ Dyadic.TSD.Partner | Gender * Stimuli.gender,
  at = list(Dyadic.TSD.Partner = seq(0, 31, length.out = 100)),
  type = "response"
) |>
  as.data.frame() |>
  ggplot(aes(
    x = Dyadic.TSD.Partner, y = rate,
    color = Stimuli.gender, fill = Stimuli.gender
  )) +
  geom_line(size = 1) +
  geom_ribbon(aes(ymin = asymp.LCL, ymax = asymp.UCL), alpha = 0.2, color = NA) +
  scale_color_manual(values = color.StimuliSex) +
  scale_fill_manual(values = color.StimuliSex) +
  facet_wrap(~Gender, ncol = 1) +
  labs(
    y = "", x = "Dyadic TSD Partner",
    title = "GLMER (Poisson)",
    color = "Stimuli Gender", fill = "Stimuli Gender"
  ) +
  theme_tq() +
  theme(legend.position = "bottom") +
  ylim(c(0.3, 5.3))

# LMM Predictions
p_m2c_lmer <- emmeans(m2c_lmer, ~ Dyadic.TSD.Partner | Gender * Stimuli.gender,
  at = list(Dyadic.TSD.Partner = seq(0, 31, length.out = 100)),
  type = "response"
) |>
  as.data.frame() |>
  ggplot(aes(
```

```
    x = Dyadic.TSD.Partner, y = emmean,
    color = Stimuli.gender, fill = Stimuli.gender
  )) +
  geom_line(size = 1) +
  geom_ribbon(aes(ymin = asymp.LCL, ymax = asymp.UCL), alpha = 0.2, color = NA) +
  scale_color_manual(values = color.StimuliSex) +
  scale_fill_manual(values = color.StimuliSex) +
  facet_wrap(~Gender, ncol = 1) +
  labs(
    y = "", x = "Dyadic TSD Partner",
    title = "LMM",
    color = "Stimuli Gender", fill = "Stimuli Gender"
  ) +
  theme_tq() +
  theme(legend.position = "bottom") +
  ylim(c(0.3, 5.3))

# Arrange plots into a single figure
p_robu_m2c <- ggarrange(p_m2c_clmm, p_m2c_poisson, p_m2c_lmer,
  common.legend = TRUE, labels = "auto", legend = "bottom", nrow = 1
)

# Display the combined figure
p_robu_m2c
```

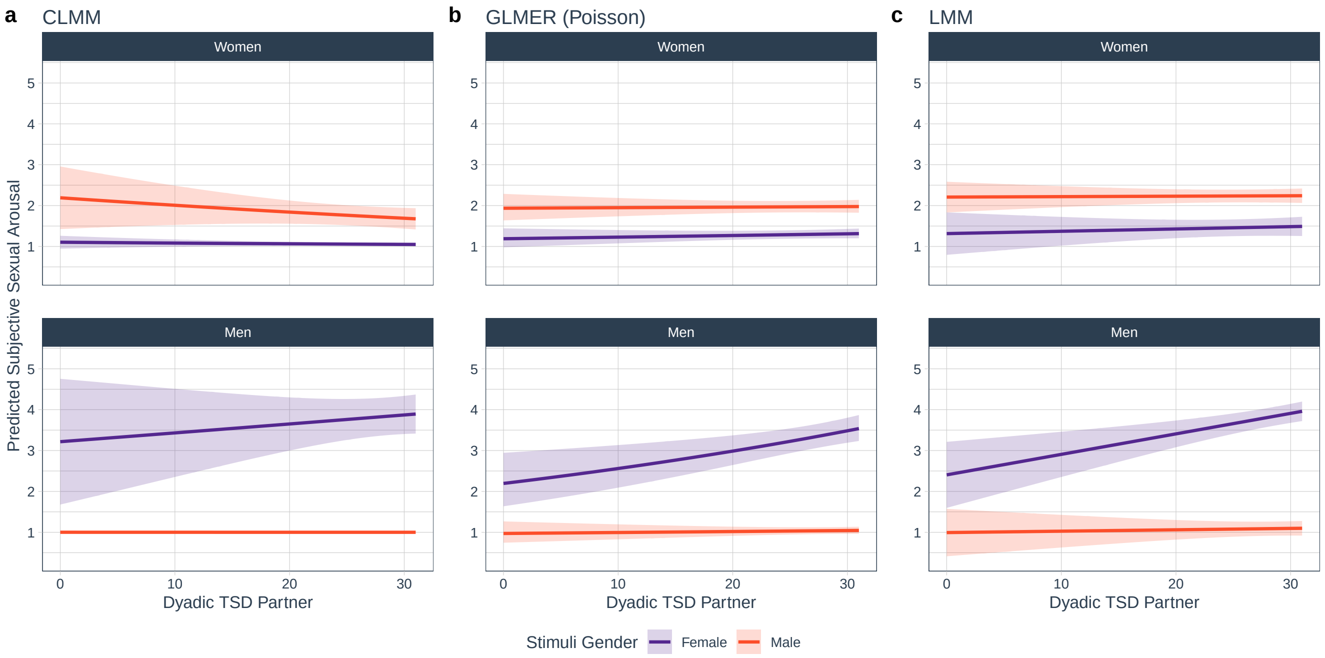

Figure S14. Predicted subjective sexual arousal as a function of Dyadic TSD Partner, modeled using three statistical approaches: **(a)** Cumulative Link Mixed Model (CLMM); **(b)** Generalized Linear Mixed Model (GLMM) with a Poisson family; **(c)** Linear Mixed Model (LMM). Lines represent predicted values, and shaded areas indicate 95% confidence intervals. The models include participant gender and stimulus gender as key factors.

4.3.5.2 Final Model: Effects of Dyadic TSD Partner on SSA Across Gender and Stimuli Gender

Given the apparent robustness of most results across models (CLMM, GLMER and LMM; Table S25, Fig. S14), we test the predictions of the hypothesis from the LMM (`m2c_lmer`).

4.3.5.2.1 Table S26. ANOVA-type table for the interaction between `Relationship type`, and `Gender`

This tables summarizes the results of the model.

```
# Generate ANOVA-type table for the final LMM model
# This summarizes the effects of Dyadic TSD Partner on SSA across Gender and Stimuli Gender
anova.sig.lmer(
  model = m2c_lmer, # Use LMM as the final model
  custom_caption = "Effects of Dyadic TSD Partner on SSA Across Gender and Stimuli Gender"
)
```

Table S26. *Effects of Dyadic TSD Partner on SSA Across Gender and Stimuli Gender*

Effect	*df*	*F*	*p*	ϵ_p^2
Dyadic TSD Partner	1, 316	6.59	**0.0107**	0.0173
Gender	1, 316	0.03	0.85	< 0.0001
Stimuli gender	1, 344.42	0.99	0.32	< 0.0001
Dyadic TSD Partner × Gender	1, 316	3.97	**0.0472**	0.0093
Dyadic TSD Partner × Stimuli gender	1, 316	8.46	**0.0039**	0.023
Gender × Stimuli gender	1, 316	20.55	**< 0.0001**	0.06
Dyadic TSD Partner × Gender × Stimuli gender	1, 316	5.70	**0.0176**	0.0146

Note: Results are Type III ANOVA. $R^2_{conditional} = 0.745$, $R^2_{marginal} = 0.329$. As effect size, we report partial epsilon squared (ϵ_p^2), a less biased estimate than η^2 (see Albers and Lakens, 2018). Significant effects are in bold.

4.3.5.2.2 ***Post-hoc* tests**

To test the hypothesis, which predicted that there would be different relationship between SSA and Dyadic TSD Partner, and that this association differ between men and women depending on the gender of stimuli, we used simple slope analysis.

```
# Perform simple slopes analysis for Dyadic TSD Partner on SSA
slop.m2c_lmer <- sim_slopes(
  m2c_lmer,
  pred = Dyadic.TSD.Partner,
  modx = Stimuli.gender,
  mod2 = Gender,
  confint = TRUE
)

# Combine results for both genders
slop.m2c_lmer.tab <- bind_rows(
  slop.m2c_lmer$slopes[[1]] |> mutate(Gender = "Women"),
  slop.m2c_lmer$slopes[[2]] |> mutate(Gender = "Men")
) |>
  mutate(
    Gender = recode_factor(Gender, Femenino = "Women", Masculino = "Men")
  ) |>
  select(Gender, `Value of Stimuli.gender`, Est., `2.5%`, `97.5%`, `t val.`, p) |>
  mutate(
    across(c(Est., `2.5%`, `97.5%`, `t val.`, p), as.numeric),
    across(c(Est., `2.5%`, `97.5%`, `t val.`), round, 2),
    sig = pval.stars(p)
  ) |>
  rename(
    "Stimuli.gender" = `Value of Stimuli.gender`,
    "Coefficient" = Est.
  )
```

```
# Create a formatted results table
slop.m2c_lmer.tab[, -c(1, 8)] |>
  mutate(p = pval.lev(p)) |>
  kable(
    booktabs = TRUE,
    align = c("l", rep("c", 5)),
    caption = "Slope for Dyadic TSD Partner on Subjective sexual arousal by
    stimuli gender and gender",
    linesep = "",
    col.names = c(
      "Stimuli gender", "$B$", "$2.5\\% CI$", "$97.5\\% CI$", "$t$", "$p$"
    ),
    escape = FALSE
  ) |>
  kable_styling(latex_options = c("HOLD_position")) |>
  pack_rows(
    group_label = "Gender: Women",
    start_row = 1, end_row = 2,
    bold = FALSE, background = "lightgray"
  ) |>
  pack_rows(
    group_label = "Gender: Men",
    start_row = 3, end_row = 4,
    bold = FALSE, background = "lightgray"
  ) |>
  footnote(
    general = "$B$ represents unstandardized coefficients. No intercept is reported as
    continuous predictors were centered and are dependent on this specific sample.",
    threeparttable = TRUE,
    footnote_as_chunk = TRUE,
    escape = FALSE
  )
```

Table S27. *Slope for Dyadic TSD Partner on Subjective sexual arousal by stimuli gender and gender*

Stimuli gender	B	$2.5\% CI$	$97.5\% CI$	t	p
Gender: Women					
Female	0.01	-0.01	0.02	0.58	0.56
Male	0.00	-0.01	0.01	0.15	0.88
Gender: Men					
Female	0.05	0.02	0.08	3.63	$<$ **0.001**
Male	0.00	-0.02	0.02	0.34	0.73

Note: B represents unstandardized coefficients. No intercept is reported as continuous predictors were centered and are dependent on this specific sample.

4.3.5.3 Figure S15. Subjective sexual arousal to erotic stimuli: Main effects and interactions

This figure summarizes the results of hypothesis 2c.

```
# Create the final plot for Dyadic TSD Partner on SSA
p_m2c.fin <- p_m2c_lmer +
  labs(
    title = "",
    y = "Predicted Subjective Sexual Arousal"
  ) +
```

```
  facet_wrap(~Gender, ncol = 2) + # Create separate facets by gender
  # Add text labels for regression slopes
  geom_text(
    data = slop.m2c_lmer.tab |> mutate(Dyadic.TSD.Partner = 2),
    mapping = aes(
      x = min(Dyadic.TSD.Partner), y = Inf,
      label = paste(
        "B = ", Coefficient,
        ", 95% CI [", `2.5%`, ", ", `97.5%`,
        "], p", ifelse(grepl("<", pe2.lev(p)), pe2.lev(p), paste0(" = ", pe2.lev(p))),
        ifelse(is.na(sig), "", sig)
      ),
      vjust = 2 + as.numeric(as.factor(Stimuli.gender)) * 2 # Stacks labels properly
    ),
    hjust = -0.1, # Align text to the left
    show.legend = FALSE
  ) +
  theme(legend.position = "bottom") # Position legend at the bottom

# Display the final figure
p_m2c.fin
```

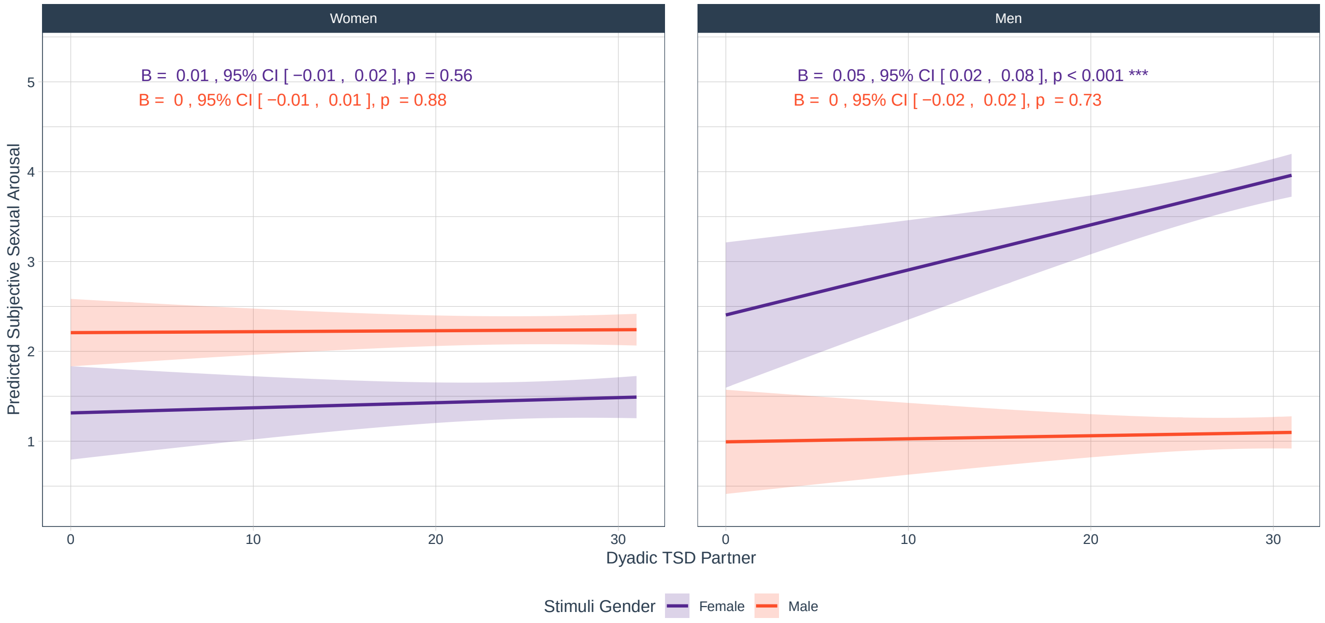

Figure S15. Predicted subjective sexual arousal as a function of Dyadic TSD Partner, modeled using aLinear Mixed Model (LMM). Lines represent predicted values, and shaded areas indicate 95% confidence intervals. The model include participant gender and stimuli gender as key factors.

4.4 Hypothesis 3: The associations between TSD dimensions and SSA toward stimuli of self-reported preferred gender will be moderated by gender and relationship status.

We tested whether the relationship between SSA and TSD varies across the three dimensions of TSD and whether these associations differ between men and women depending on whether they were single or not, but only in responses toward stimuli of the preferred gender. This is a fully exploratory hypothesis, for which no directional predictions were made, beyond an interaction between the TSD dimension, gender, and relationship status. As

with the case of Hypothesys 2 (section 4.3), fitted separate models for each TSD dimension:

- **H3a**: Solitary TSD and SSA
- **H3b**: Dyadic TSD toward an attractive person
- **H3c**: Dyadic TSD toward a partner

To examine this hypothesis, we modeled the effects of each of the three TSD dimension scores, gender, relationship status, and their interactions, on SSA towars stimuli of the self-reported preferred gender. We included random intercepts for each stimulus, as well as random intercepts for each participant.

4.4.1 Modeling Approach

Following the strategy employed for Hypothesys 2 (section 4.3), and given that SSA is an ordinal variable with seven ordered levels, we fitted diferent models using three different approaches to ensure the robustness of our results:

1. Cumulative Link Mixed Model (CLMM), using the `clmm` function from the package `ordinal` (Christensen, 2023)
2. Generalized Mixed Model (GLMM) with a Poisson family, using the `glmer` function from the package `lme4` (Bates et al., 2015)
3. Linear mixed model (LMM), using the `lmer` function from the package `lmerTest` (Kuznetsova et al., 2017)

The results across these models were largely consistent, indicating robustness in our findings. For clarity and interpretability, we primarily base our inferences on the LMM, as it provides the most straightforward interpretation and has a wider range of available functions in R for extracting model information.

4.4.2 Data

We created a new dataset by selecting, once again, only responses to erotic stimuli but this time also filtering only responses to stimuli of the preferred gender. We also renamed key variables to remove spaces for compatibility with certain functions, and created a factor version of Subjective sexual arousal for use in the CLMM model.

```
# Filter dataset to include only responses to erotic stimuli
dat_m3 <- dat |>
  filter(`Stimuli content` == "Erotic" &
    `Stimuli gender` == `Preferred gender`) |>
  rename(
    Subjective.sexual.arousal = `Subjective sexual arousal`,
    Solitary.TSD = `Solitary sexual desire`,
    Dyadic.TSD.Attractive.Person = `Dyadic sexual desire (Attractive person)`,
    Dyadic.TSD.Partner = `Dyadic sexual desire (Partner)`,
    Stimuli.code = `Stimuli code`
  ) |>
  mutate(Subjective.sexual.arousal.factor = as.factor(Subjective.sexual.arousal))
```

4.4.3 Hypothesis 3a: Solitary TSD

4.4.3.1 Model Robustness: Examining the Effects of Solitary TSD on SSA Across Gender and Stimuli Gender To assess the robustness of our findings, we fitted three different models examining how Solitary TSD predicts SSA, considering variations by gender and stimuli gender:

1. Cumulative Link Mixed Model (CLMM) – `m3a_clmm` (for ordinal outcomes, using a probit link).
2. Generalized Linear Mixed Model (GLMM) with Poisson family – `m3a_poisson` (treating SSA as a count variable).
3. Linear Mixed Model (LMM) – `m3a_lmer` (treating SSA as a continuous variable).

Note on model structure:

> Including a random intercept for Stimuli (in addition to Participant) resulted in convergence failures in the cumulative link mixed model (CLMM), which could not be resolved by changing optimisers or other strategies. To ensure model stability and convergence, we retained only the random intercept for

Participant. This decision was made after verifying in simpler models that including Stimuli did not qualitatively alter the fixed-effects estimates.

```
# (1) Cumulative Link Mixed Model (CLMM) - Ordinal model with probit link
m3a_clmm <- clmm(
  Subjective.sexual.arousal.factor ~ Solitary.TSD * Gender * Relationship +
    # (1 | Stimuli.code) + # Random intercept for Stimuli impeded convergence
    (1 | Participant), # Random intercept for Participant
  data = dat_m3,
  link = "probit",
  control = list(method = "nlminb") # Optimiser that often helps convergence
)

# (2) Generalized Linear Mixed Model (GLMM) - Poisson regression for count data
m3a_poisson <- glmer(
  Subjective.sexual.arousal ~ Solitary.TSD * Gender * Relationship +
    (1 | Stimuli.code) + # Random intercept for Stimuli
    (1 | Participant), # Random intercept & slope for Relationship status
  data = dat_m3,
  family = poisson # Poisson distribution for count data
)

# (3) Linear Mixed Model (LMM) - Continuous approximation
m3a_lmer <- lmer(
  Subjective.sexual.arousal ~ Solitary.TSD * Gender * Relationship +
    (1 | Stimuli.code) + # Random intercept for Stimuli
    (1 | Participant), # Random intercept & slope for Relationship status
  data = dat_m3,
  control = lmerControl(optimizer = "bobyqa") # Use 'bobyqa' optimizer for stability
)
```

4.4.3.1.1 Table S28. ANOVA-type table of fixed effects (main effects and interactions) across the three fitted models As shown in the table below, the pattern of significant effects remains consistent across all three models, except for the main effect of gender, which is not significant in the CLMM.

```
# Compare fixed effects across the three models (CLMM, GLMM, LMM)
anova.comp(
  CLMMmod = m3a_clmm, # Cumulative Link Mixed Model (ordinal outcome)
  GLMERmod = m3a_poisson, # Generalized Linear Mixed Model (Poisson family)
  LMERmod = m3a_lmer, # Linear Mixed Model (continuous outcome)
  hypothesis = "3a" # Specifies hypothesis 3a for tracking
)
```

Table S28. *Comparison of fixed effects across the three models for Hypothesis 3a: CLMM, GLMM (Poisson), and LMM.*

	CLMM			GLMER (Poisson)			LMM		
Effect	*df*	χ^2	*p*	*df*	χ^2	*p*	*df*	*F*	*p*
Solitary TSD	1	10.812	**0.001**	1	9.473	**0.0021**	1, 315	6.881	**0.0091**
Gender	1	18.645	**< 0.0001**	1	17.941	**< 0.0001**	1, 355.34	14.100	**< 0.001**
Relationship	1	0.001	0.97	1	0.018	0.89	1, 314.95	0.337	0.56
Solitary TSD × Gender	1	1.554	0.21	1	1.291	0.26	1, 315.23	0.071	0.79
Solitary TSD × Relationship	1	0.071	0.79	1	0.180	0.67	1, 314.95	0.531	0.47
Gender × Relationship	1	3.058	0.08	1	2.152	0.14	1, 314.95	2.953	0.09
Solitary TSD × Gender × Relationship	1	2.343	0.13	1	1.443	0.23	1, 315.08	2.023	0.16

Note: For CLMM and GLMER (Poisson) models, results are Analysis of Deviance (Type III Wald chi-square tests), while for LMM, results are from an Analysis of Variance (Type III ANOVA with Satterthwaite's method). Significant effects are in bold.

4.4.3.1.2 Figure S16: Model-based predictions for Hypothesis 3a.

This figure presents model-based predictions of subjective sexual arousal as a function of Solitary TSD, across different relationship status and participant genders. The three subplots correspond to the three statistical models used for analysis: (a) Cumulative Link Mixed Model (CLMM), (b) Generalized Linear Mixed Model (GLMM, Poisson), and (c) Linear Mixed Model (LMM). Shaded areas represent 95% confidence intervals.

```
# CLMM Predictions: Estimated marginal means for Solitary TSD across gender & relationship
p_m3a_clmm <- emmeans(m3a_clmm, ~ Solitary.TSD | Gender * Relationship,
  at = list(Solitary.TSD = seq(0, 31, length.out = 100)), # Generate values for smooth curve
  mode = "mean.class" # Predict mean response category
) |>
  as.data.frame() |> # Convert results into a dataframe for plotting
  ggplot(aes(x = Solitary.TSD, y = mean.class, color = Relationship, fill = Relationship)) +
  geom_line(size = 1) + # Plot predicted means as lines
  geom_ribbon(aes(ymin = asymp.LCL, ymax = asymp.UCL, fill = Relationship),
    alpha = 0.2, color = NA # Add confidence interval as shaded ribbon
  ) +
  scale_color_manual(values = color.Relationship) + # Use predefined colors
  scale_fill_manual(values = color.Relationship) +
  facet_wrap(~Gender, ncol = 1) + # Create separate plots for each gender
  labs(y = "Predicted Subjective Sexual Arousal", x = "Solitary TSD",
       title = "CLMM") +
  theme_tq() + # Apply custom theme
  theme(legend.position = "bottom") +
  ylim(c(0.3, 6)) # Set Y-axis limits

# Poisson GLMM Predictions: Similar setup but with a Poisson distribution
p_m3a_poisson <- emmeans(m3a_poisson, ~ Solitary.TSD | Gender * Relationship,
  at = list(Solitary.TSD = seq(0, 31, length.out = 100)), type = "response"
) |>
  as.data.frame() |>
  ggplot(aes(x = Solitary.TSD, y = rate, color = Relationship, fill = Relationship)) +
  geom_line(size = 1) +
  geom_ribbon(aes(ymin = asymp.LCL, ymax = asymp.UCL, fill = Relationship),
    alpha = 0.2, color = NA
  ) +
  scale_color_manual(values = color.Relationship) +
  scale_fill_manual(values = color.Relationship) +
  facet_wrap(~Gender, ncol = 1) +
  labs(y = "", x = "Solitary TSD", title = "GLMER (Poisson)") +
  theme_tq() +
  theme(legend.position = "bottom") +
  ylim(c(0.3, 6))

# LMM Predictions: Continuous outcome using LMM
p_m3a_lmer <- emmeans(m3a_lmer, ~ Solitary.TSD | Gender * Relationship,
  at = list(Solitary.TSD = seq(0, 31, length.out = 100)), type = "response"
) |>
  as.data.frame() |>
  ggplot(aes(x = Solitary.TSD, y = emmean, color = Relationship, fill = Relationship)) +
  geom_line(size = 1) +
  geom_ribbon(aes(ymin = asymp.LCL, ymax = asymp.UCL, fill = Relationship),
    alpha = 0.2, color = NA
  ) +
  scale_color_manual(values = color.Relationship) +
  scale_fill_manual(values = color.Relationship) +
  facet_wrap(~Gender, ncol = 1) +
```

```
  labs(y = "", x = "Solitary TSD", title = "LMM") +
  theme_tq() +
  theme(legend.position = "bottom") +
  ylim(c(0.3, 6))

# Arrange plots into a single figure
p_robu_m3a <- ggarrange(
  p_m3a_clmm, p_m3a_poisson, p_m3a_lmer, # Combine all three models
  common.legend = TRUE, # Share legend across plots
  labels = "auto", # Automatically label subfigures (a, b, c)
  legend = "bottom",
  nrow = 1 # Arrange in a single row
)

# Display the combined figure
p_robu_m3a
```

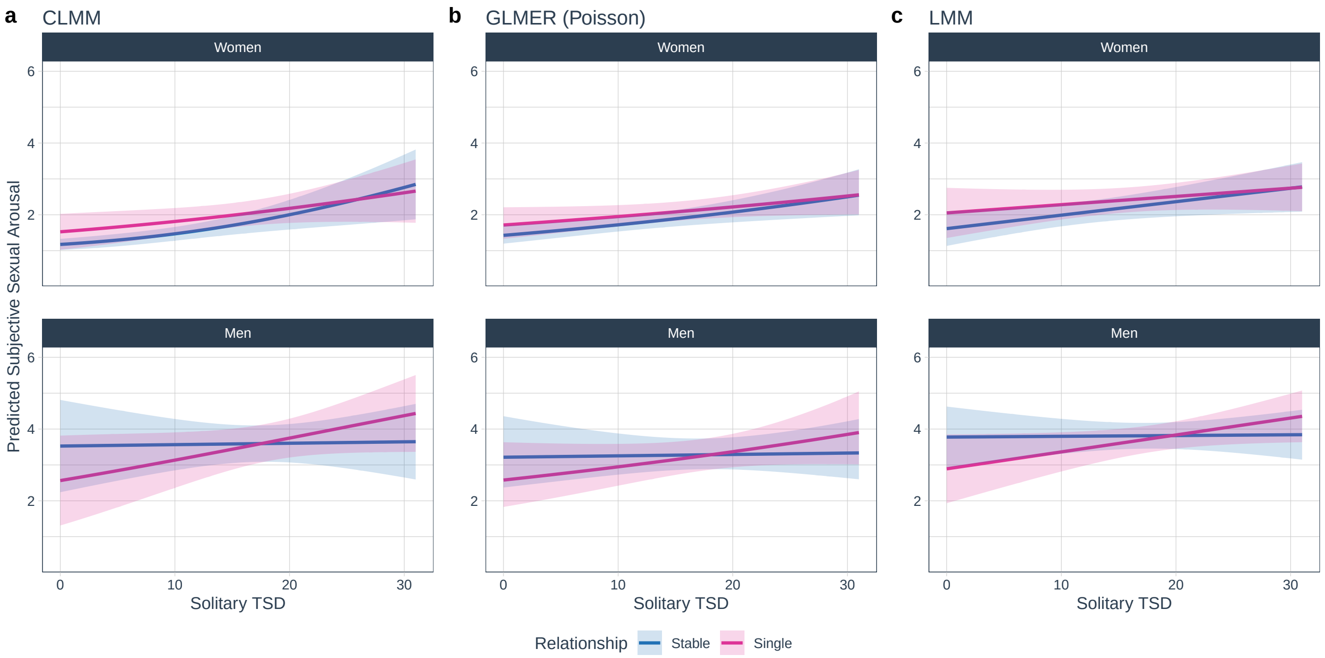

Figure S16. Predicted subjective sexual arousal as a function of Solitary TSD, modeled using three statistical approaches: **(a)** Cumulative Link Mixed Model (CLMM); **(b)** Generalized Linear Mixed Model (GLMM) with a Poisson family; **(c)** Linear Mixed Model (LMM). Lines represent predicted values, and shaded areas indicate 95% confidence intervals. The models include participant gender and relationship status as key factors.

4.4.3.2 Final Model: Effects of Solitary TSD on SSA Across Gender and Stimuli Gender Given the apparent robustness of most results across models (CLMM, GLMER and LMM; Table S28, Fig. S16), we test the predictions of the hypothesis from the LMM (`m3a_lmer`).

4.4.3.2.1 Table S29. ANOVA-type table for the interaction between `Relationship type`, and `Gender` This tables summarizes the results of the model.

```
# Generate ANOVA-type table for the final LMM model
# This summarizes the effects of Solitary TSD on SSA across Gender and Relationship
anova.sig.lmer(
  model = m3a_lmer, # Use LMM as the final model
  custom_caption = "Effects of Solitary TSD on SSA Across Gender and Relationship"
```

```
)
```

Table S29. *Effects of Solitary TSD on SSA Across Gender and Relationship*

Effect	*df*	*F*	*p*	ϵ_p^2
Solitary TSD	1, 315	6.88	**0.0091**	0.0183
Gender	1, 355.34	14.10	**< 0.001**	0.0355
Relationship	1, 314.95	0.34	0.56	< 0.0001
Solitary TSD × Gender	1, 315.23	0.07	0.79	< 0.0001
Solitary TSD × Relationship	1, 314.95	0.53	0.47	< 0.0001
Gender × Relationship	1, 314.95	2.95	0.09	0.0061
Solitary TSD × Gender × Relationship	1, 315.08	2.02	0.16	0.0032

Note: Results are Type III ANOVA. $R^2_{conditional} = 0.72$, $R^2_{marginal} = 0.171$. As effect size, we report partial epsilon squared (ϵ_p^2), a less biased estimate than η^2 (see Albers and Lakens, 2018). Significant effects are in bold.

4.4.3.2.2 *Post-hoc* tests To test the hypothesis, which predicted that there would be different relationship between SSA and solitary TSD, and that this association differ between men and women depending on the gender of stimuli, we used simple slope analysis.

```
# Compute simple slopes for Solitary TSD on SSA, moderated by Gender and Relationship
slop.m3a_lmer <- sim_slopes(m3a_lmer,
  pred = Solitary.TSD, # Predictor: Solitary TSD
  modx = Relationship, # Moderator 1: Relationship status
  mod2 = Gender, # Moderator 2: Gender
  confint = TRUE
) # Compute confidence intervals

# Extract slopes separately for Women and Men, then combine into a single table
slop.m3a_lmer.tab <- bind_rows(
  slop.m3a_lmer$slopes[[1]] |>
    mutate(Gender = "Women"),
  slop.m3a_lmer$slopes[[2]] |>
    mutate(Gender = "Men")
) |>
  mutate(Gender = recode_factor(Gender,
    Femenino = "Women",
    Masculino = "Men"
  )) |>
  select(8, 1:2, 4:7) |> # Select relevant columns
  mutate(across(3:7, as.numeric)) |> # Ensure numeric format
  mutate(across(3:6, round, 2)) |> # Round coefficients and confidence intervals
  mutate(sig = pval.stars(p)) |> # Add significance stars
  rename("Relationship" = "Value of Relationship") |> # Rename columns for clarity
  rename(Coefficient = Est.) # Rename estimated coefficient column

# Format and display the table using `kable`
slop.m3a_lmer.tab[, -c(1, 8)] |> # Remove first and eighth columns
  mutate(p = pval.lev(p)) |> # Format p-values
  kable(
    booktabs = TRUE,
    align = c("l", rep("c", 5)),
    caption = "Slope for Solitary TSD on
      Subjective sexual arousal by relationship status and gender",
    linesep = "",
```

```
    col.names = c(
      "Relationship status",
      "$B$",
      "$2.5\\% CI$",
      "$97.5\\% CI$",
      "$t$",
      "$p$"
    ),
    escape = FALSE
  ) |>
  kable_styling(latex_options = c("HOLD_position")) |>
  pack_rows(
    group_label = "Gender: Women",
    start_row = 1,
    end_row = 2,
    bold = FALSE,
    background = "lightgray"
  ) |>
  pack_rows(
    group_label = "Gender: Men",
    start_row = 3,
    end_row = 4,
    bold = FALSE,
    background = "lightgray"
  ) |>
  footnote(
    general = "$B$ are unstandardized coefficients.
           No intercept is reported as continuous predictors were centered
           and are dependent on this specific sample.",
    threeparttable = TRUE,
    footnote_as_chunk = TRUE,
    escape = FALSE
  )
```

Table S30. *Slope for Solitary TSD on Subjective sexual arousal by relationship status and gender*

Relationship status	B	$2.5\% CI$	$97.5\% CI$	t	p
Gender: Women					
Stable	0.04	0.01	0.07	2.31	**0.0217**
Single	0.02	-0.01	0.06	1.19	0.23
Gender: Men					
Stable	0.00	-0.04	0.05	0.10	0.92
Single	0.05	0.00	0.10	1.91	0.06

Note: B are unstandardized coefficients. No intercept is reported as continuous predictors were centered and are dependent on this specific sample.

4.4.3.3 Figure S17. Subjective sexual arousal to erotic stimuli: Main effects and interactions

This figure summarizes the results of hypothesis 3a.

```
# Generate final plot for Solitary TSD effects on SSA
# Includes interaction between Relationship status and Gender
p_m3a.fin <- p_m3a_lmer +
  labs(
    title = "",
```

```
    y = "Predicted Subjective Sexual Arousal"
  ) +
  facet_wrap(~Gender, ncol = 2) + # Separate plots by Gender

  # Add text labels with regression coefficients, confidence intervals, and p-values
  geom_text(
    data = slop.m3a_lmer.tab |>
      mutate(Solitary.TSD = 2), # Assign a reference value for positioning
    mapping = aes(
      x = min(Solitary.TSD), y = Inf,
      label = paste(
        "B = ", Coefficient,
        ", IC 95%[", `2.5%`, ", ", `97.5%`,
        "], p",
        ifelse(grepl("<", pe2.lev(p)), pe2.lev(p),
          paste0(" = ", pe2.lev(p))
        ),
        ifelse(is.na(sig), "", sig)
      ),
      vjust = 2 + as.numeric(as.factor(Relationship)) * 2
    ),
    # Adjust vertical positioning based on Relationship status
    hjust = -0.1, # Left-align text labels
    show.legend = FALSE
  ) + # Hide legend for text labels

  theme(legend.position = "bottom") # Move legend to bottom

# Display the final plot
p_m3a.fin
```

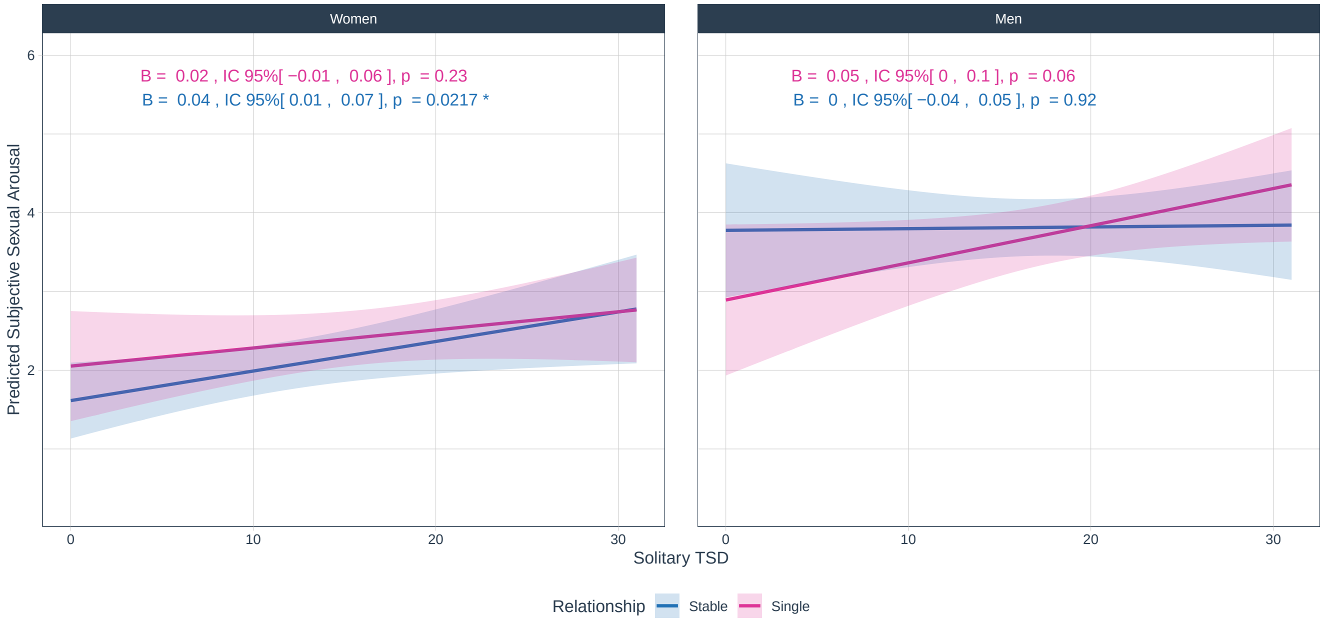

Figure S17. Predicted subjective sexual arousal as a function of Solitary TSD, modeled using aLinear Mixed Model (LMM). Lines represent predicted values, and shaded areas indicate 95% confidence intervals. The model include participant gender and relationship status as key factors.

4.4.4 Hypothesis 3b: Dyadic TSD Attractive Person

4.4.4.1 Model Robustness: Examining the Effects of Dyadic TSD Attractive Person on SSA Across Gender and Stimuli Gender To assess the robustness of our findings, we fitted three different models examining how Dyadic TSD Attractive Person predicts SSA, considering variations by gender and stimuli gender:

1. Cumulative Link Mixed Model (CLMM) – `m3b_clmm` (for ordinal outcomes, using a probit link).
2. Generalized Linear Mixed Model (GLMM) with Poisson family – `m3b_poisson` (treating SSA as a count variable).
3. Linear Mixed Model (LMM) – `m3b_lmer` (treating SSA as a continuous variable).

```
# Fit three different models to assess robustness of Dyadic TSD Attractive Person on SSA

# (1) Cumulative Link Mixed Model (CLMM) - Ordinal model with probit link
m3b_clmm <- clmm(
  Subjective.sexual.arousal.factor ~ Dyadic.TSD.Attractive.Person * Gender * Relationship +
    (1 | Stimuli.code) + # Random intercept for Stimuli
    (1 | Participant), # Random intercept & slope for Relationship status
  data = dat_m3,
  link = "probit", # Use probit link for ordinal regression
  control = list(method = "nlminb") # Use 'nlminb' optimizer for better convergence
)

# (2) Generalized Linear Mixed Model (GLMM) - Poisson regression for count data
m3b_poisson <- glmer(
  Subjective.sexual.arousal ~ Dyadic.TSD.Attractive.Person * Gender * Relationship +
    (1 | Stimuli.code) + # Random intercept for Stimuli
    (1 | Participant), # Random intercept & slope for Relationship status
  data = dat_m3,
  family = poisson # Poisson distribution for count data
)

# (3) Linear Mixed Model (LMM) - Continuous approximation
m3b_lmer <- lmer(
  Subjective.sexual.arousal ~ Dyadic.TSD.Attractive.Person * Gender * Relationship +
    (1 | Stimuli.code) + # Random intercept for Stimuli
    (1 | Participant), # Random intercept & slope for Relationship status
  data = dat_m3,
  control = lmerControl(optimizer = "bobyqa") # Use 'bobyqa' optimizer for stability
)
```

4.4.4.1.1 Table S31. ANOVA-type table of fixed effects (main effects and interactions) across the three fitted models As shown in the table below, the pattern of significant effects remains consistent across all three models, except for the main effect of gender, which is not significant in the CLMM.

```
# Compare fixed effects across CLMM, GLMM, and LMM models for Hypothesis 3b
# This function generates an ANOVA-type table summarizing main effects and interactions
anova.comp(
  CLMMmod = m3b_clmm, # Cumulative Link Mixed Model (CLMM)
  GLMERmod = m3b_poisson, # Generalized Linear Mixed Model (GLMM, Poisson)
  LMERmod = m3b_lmer, # Linear Mixed Model (LMM)
  hypothesis = "3b" # Hypothesis identifier for documentation purposes
)
```

Table S31. *Comparison of fixed effects across the three models for Hypothesis 3b: CLMM, GLMM (Poisson), and LMM.*

	CLMM			GLMER (Poisson)			LMM		
Effect	*df*	χ^2	*p*	*df*	χ^2	*p*	*df*	*F*	*p*
Dyadic TSD Attractive Person	1	47.486	**< 0.0001**	1	47.634	**< 0.0001**	1, 315.21	46.796	**< 0.0001**
Gender	1	4.636	**0.0313**	1	4.229	**0.0397**	1, 354.77	1.207	0.27
Relationship	1	0.928	0.34	1	0.353	0.55	1, 315.16	0.126	0.72
Dyadic TSD Attractive Person × Gender	1	0.452	0.5	1	1.391	0.24	1, 314.97	7.064	**0.0083**
Dyadic TSD Attractive Person × Relationship	1	0.525	0.47	1	0.130	0.72	1, 315.21	0.084	0.77
Gender × Relationship	1	0.000	0.99	1	0.005	0.94	1, 315.06	0.001	0.97
Dyadic TSD Attractive Person × Gender × Relationship	1	0.215	0.64	1	0.213	0.64	1, 314.97	0.339	0.56

Note: For CLMM and GLMER (Poisson) models, results are Analysis of Deviance (Type III Wald chi-square tests), while for LMM, results are from an Analysis of Variance (Type III ANOVA with Satterthwaite's method). Significant effects are in bold.

4.4.4.1.2 Figure S18: Model-based predictions for Hypothesis 3b. This figure presents model-based predictions of subjective sexual arousal as a function of Dyadic TSD Attractive Person, across different relationship status and participant genders. The three subplots correspond to the three statistical models used for analysis: (a) Cumulative Link Mixed Model (CLMM), (b) Generalized Linear Mixed Model (GLMM, Poisson), and (c) Linear Mixed Model (LMM). Shaded areas represent 95% confidence intervals.

```
# Generate model-based predictions for Hypothesis 3b
# Predict SSA based on Dyadic TSD Attractive Person across gender and relationship status

# CLMM Predictions
p_m3b_clmm <- emmeans(m3b_clmm, ~ Dyadic.TSD.Attractive.Person | Gender * Relationship,
  at = list(Dyadic.TSD.Attractive.Person = seq(0, 31, length.out = 100)),
  mode = "mean.class" # Compute predicted mean response categories
) |>
  as.data.frame() |> # Convert predictions to a dataframe for ggplot
  ggplot(aes(
    x = Dyadic.TSD.Attractive.Person, y = mean.class,
    color = Relationship, fill = Relationship
  )) +
  geom_line(size = 1) + # Plot predicted response line
  geom_ribbon(aes(ymin = asymp.LCL, ymax = asymp.UCL, fill = Relationship),
    alpha = 0.2, color = NA # Add confidence interval as shaded ribbon
  ) +
  scale_color_manual(values = color.Relationship) + # Apply custom colors
  scale_fill_manual(values = color.Relationship) +
  facet_wrap(~Gender, ncol = 1) + # Create separate plots for each gender
  labs(
    y = "Predicted Subjective Sexual Arousal", x = "Attractive Person Sexual Desire",
    title = "CLMM"
  ) +
  theme_tq() + # Apply custom theme
  theme(legend.position = "bottom") +
  ylim(c(0.3, 7)) # Set Y-axis limits

# Poisson GLMM Predictions
p_m3b_poisson <- emmeans(m3b_poisson, ~ Dyadic.TSD.Attractive.Person | Gender * Relationship,
  at = list(Dyadic.TSD.Attractive.Person = seq(0, 31, length.out = 100)),
  type = "response" # Compute response-scale predictions
) |>
  as.data.frame() |>
  ggplot(aes(
    x = Dyadic.TSD.Attractive.Person, y = rate,
    color = Relationship, fill = Relationship
```

```
  )) +
  geom_line(size = 1) +
  geom_ribbon(aes(ymin = asymp.LCL, ymax = asymp.UCL, fill = Relationship),
    alpha = 0.2, color = NA
  ) +
  scale_color_manual(values = color.Relationship) +
  scale_fill_manual(values = color.Relationship) +
  facet_wrap(~Gender, ncol = 1) +
  labs(
    y = "", x = "Attractive Person Sexual Desire",
    title = "GLMER (Poisson)"
  ) +
  theme_tq() +
  theme(legend.position = "bottom") +
  ylim(c(0.3, 7))

# LMM Predictions
p_m3b_lmer <- emmeans(m3b_lmer, ~ Dyadic.TSD.Attractive.Person | Gender * Relationship,
  at = list(Dyadic.TSD.Attractive.Person = seq(0, 31, length.out = 100)),
  type = "response"
) |>
  as.data.frame() |>
  ggplot(aes(
    x = Dyadic.TSD.Attractive.Person, y = emmean,
    color = Relationship, fill = Relationship
  )) +
  geom_line(size = 1) +
  geom_ribbon(aes(ymin = asymp.LCL, ymax = asymp.UCL, fill = Relationship),
    alpha = 0.2, color = NA
  ) +
  scale_color_manual(values = color.Relationship) +
  scale_fill_manual(values = color.Relationship) +
  facet_wrap(~Gender, ncol = 1) +
  labs(
    y = "", x = "Attractive Person Sexual Desire",
    title = "LMM"
  ) +
  theme_tq() +
  theme(legend.position = "bottom") +
  ylim(c(0.3, 7))

# Arrange Plots into a Single Figure
p_robu_m3b <- ggarrange(p_m3b_clmm, p_m3b_poisson, p_m3b_lmer, # Combine plots side by side
  common.legend = TRUE, # Share legend across plots
  labels = "auto", # Automatically label subfigures (a, b, c)
  legend = "bottom",
  nrow = 1 # Arrange in a single row
)

# Display the combined figure
p_robu_m3b
```

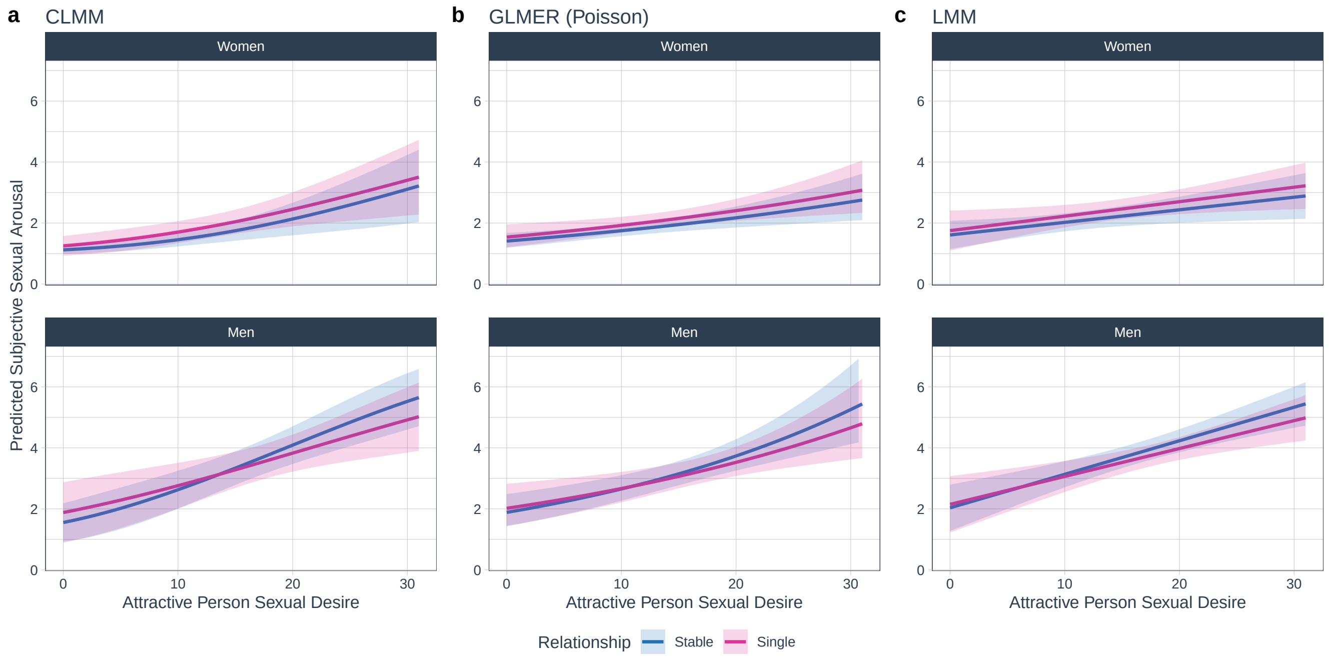

Figure S18. Predicted subjective sexual arousal as a function of Dyadic TSD Attractive Person, modeled using three statistical approaches: **(a)** Cumulative Link Mixed Model (CLMM); **(b)** Generalized Linear Mixed Model (GLMM) with a Poisson family; **(c)** Linear Mixed Model (LMM). Lines represent predicted values, and shaded areas indicate 95% confidence intervals. The models include participant gender and relationship status as key factors.

4.4.4.2 Final Model: Effects of Dyadic TSD Attractive Person on SSA Across Gender and Stimuli Gender

Given the apparent robustness of most results across models (CLMM, GLMER and LMM; Table S31, Fig. S18), we test the predictions of the hypothesis from the LMM (`m3b_lmer`).

4.4.4.2.1 Table S32. ANOVA-type table for the interaction between `Relationship type`, and `Gender`

This tables summarizes the results of the model.

```
# Generate ANOVA-type table for the final LMM model
# This summarizes the effects of Dyadic TSD Attractive Person on SSA
# across Gender and Relationship Status
anova.sig.lmer(
  model = m3b_lmer, # Use LMM as the final model
  custom_caption = "Effects of Dyadic TSD Attractive Person on SSA
  Across Gender and Stimuli Gender"
)
```

Table S32. *Effects of Dyadic TSD Attractive Person on SSA Across Gender and Stimuli Gender*

Effect	*df*	*F*	*p*	ϵ_p^2
Dyadic TSD Attractive Person	1, 315.21	46.80	**< 0.0001**	0.13
Gender	1, 354.77	1.21	0.27	< 0.001
Relationship	1, 315.16	0.13	0.72	< 0.0001
Dyadic TSD Attractive Person × Gender	1, 314.97	7.06	**0.0083**	0.0188
Dyadic TSD Attractive Person × Relationship	1, 315.21	0.08	0.77	< 0.0001
Gender × Relationship	1, 315.06	0.00	0.97	< 0.0001
Dyadic TSD Attractive Person × Gender × Relationship	1, 314.97	0.34	0.56	< 0.0001

Note: Results are Type III ANOVA. $R^2_{conditional} = 0.719$, $R^2_{marginal} = 0.225$. As effect size, we report partial epsilon squared (ϵ_p^2), a less biased estimate than η^2 (see Albers and Lakens, 2018). Significant effects are in bold.

4.4.4.2.2 *Post-hoc* tests To test the hypothesis, which predicted that there would be different relationship between SSA and Dyadic TSD Attractive Person, and that this association differ between men and women depending on the gender of stimuli, we used simple slope analysis.

```
# Perform simple slopes analysis to examine the effect of Dyadic TSD Attractive Person
# on SSA across Gender and Relationship Status
slop.m3b_lmer <- sim_slopes(m3b_lmer,
  pred = Dyadic.TSD.Attractive.Person, # Predictor variable
  modx = Relationship, # Moderator 1: Relationship status
  mod2 = Gender, # Moderator 2: Gender
  confint = TRUE
) # Compute confidence intervals

# Combine slopes for Women and Men into a single dataframe for easier processing
slop.m3b_lmer.tab <- bind_rows(
  slop.m3b_lmer$slopes[[1]] |>
    mutate(Gender = "Women"),
  slop.m3b_lmer$slopes[[2]] |>
    mutate(Gender = "Men")
) |>
  # Recoding Gender labels to ensure consistency
  mutate(Gender = recode_factor(Gender, Femenino = "Women", Masculino = "Men")) |>
  # Select relevant columns (drop unnecessary ones)
  select(8, 1:2, 4:7) |>
  # Convert selected columns to numeric
  mutate(across(3:7, as.numeric)) |>
  # Round relevant columns for cleaner presentation
  mutate(across(3:6, round, 2)) |>
  # Compute significance stars for p-values
  mutate(sig = pval.stars(p)) |>
  # Rename columns for clarity
  rename("Relationship" = "Value of Relationship") |>
  rename(Coefficient = Est.)

# Format the table for display using kable
slop.m3b_lmer.tab[, -c(1, 8)] |>
  # Format p-values for readability
  mutate(p = pval.lev(p)) |>
  # Create a LaTeX-formatted table with proper alignment and captions
  kable(
    booktabs = TRUE,
    align = c("l", rep("c", 5)),
    caption = "Slope for Dyadic TSD Attractive Person on
        Subjective sexual arousal by stimuli gender and gender",
    linesep = "",
    col.names = c(
      "Relationship status", "$B$", "$2.5\\% CI$",
      "$97.5\\% CI$", "$t$", "$p$"
    ),
    escape = FALSE
  ) |>
  # Apply LaTeX styling options
  kable_styling(latex_options = c("HOLD_position")) |>
  # Group rows by gender for better readability
  pack_rows(
    group_label = "Gender: Women",
    start_row = 1,
```

```
    end_row = 2,
    bold = FALSE,
    background = "lightgray"
  ) |>
  pack_rows(
    group_label = "Gender: Men",
    start_row = 3,
    end_row = 4,
    bold = FALSE,
    background = "lightgray"
  ) |>
  # Add a footnote explaining coefficient interpretation
  footnote(
    general = "$B$ are unstandardized coefficients.
           No intercept is reported as continuous predictors were centered
           and are dependent on this specific sample.",
    threeparttable = TRUE,
    footnote_as_chunk = TRUE,
    escape = FALSE
  )
```

Table S33. *Slope for Dyadic TSD Attractive Person on Subjective sexual arousal by stimuli gender and gender*

Relationship status	B	2.5%CI	97.5%CI	t	p
Gender: Women					
Stable	0.04	0.01	0.08	2.39	**0.0174**
Single	0.05	0.01	0.09	2.31	**0.0218**
Gender: Men					
Stable	0.11	0.07	0.15	5.13	**$<$ 0.0001**
Single	0.09	0.04	0.14	3.68	**$<$ 0.001**

Note: B are unstandardized coefficients. No intercept is reported as continuous predictors were centered and are dependent on this specific sample.

4.4.4.3 Figure S19. Subjective sexual arousal to erotic stimuli: Main effects and interactions This figure summarizes the results of hypothesis 3b.

```
# Generate a plot visualizing the relationship between Dyadic TSD Attractive Person
# and SSA across Gender and Relationship Status
p_m3b.fin <- p_m3b_lmer +
  # Remove plot title and set Y-axis label
  labs(
    title = "",
    y = "Predicted Subjective Sexual Arousal"
  ) +
  # Create separate facets for each Gender
  facet_wrap(~Gender, ncol = 2) +
  # Add text labels displaying regression coefficients and confidence intervals
  geom_text(
    data = slop.m3b_lmer.tab |>
      mutate(Dyadic.TSD.Attractive.Person = 2),
    mapping = aes(
      x = min(Dyadic.TSD.Attractive.Person), y = Inf,
      label = paste(
        "B = ", Coefficient,
```

```
        ", IC 95%[", `2.5%`, ", ", `97.5%`,
        "], p",
        ifelse(grepl("<", pe2.lev(p)), pe2.lev(p),
          paste0(" = ", pe2.lev(p))
        ),
        ifelse(is.na(sig), "", sig)
      ),
      # Adjust vertical position to prevent overlapping
      vjust = 2 + as.numeric(as.factor(Relationship)) * 2
    ),
    hjust = -0.1, # Align text labels to the left
    # size = 3,  # Uncomment if size adjustment is needed
    show.legend = FALSE
  ) +
  # Set legend position at the bottom
  theme(legend.position = "bottom")

# Display the final plot
p_m3b.fin
```

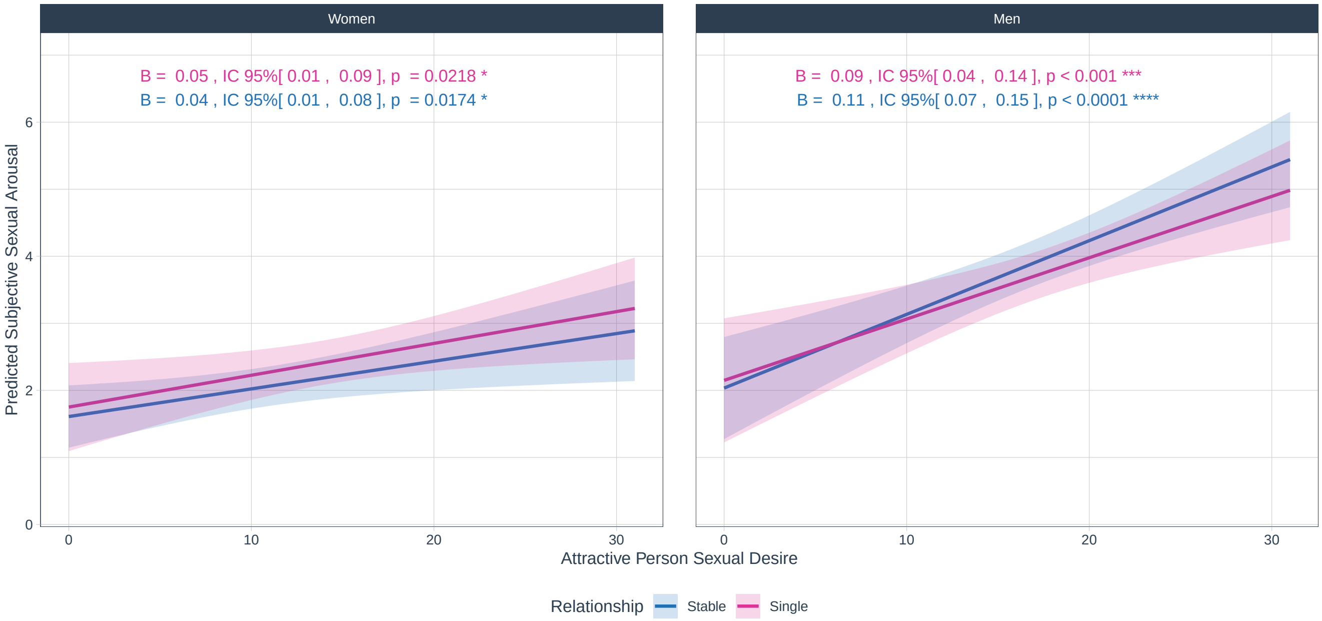

Figure S19. Predicted subjective sexual arousal as a function of Dyadic TSD Partner, modeled using aLinear Mixed Model (LMM). Lines represent predicted values, and shaded areas indicate 95% confidence intervals. The model include participant gender and relationship status as key factors.

4.4.5 Hypothesis 3c: Dyadic TSD Partner

4.4.5.1 Model Robustness: Examining the Effects of Dyadic TSD Partner on SSA Across Gender and Stimuli Gender To assess the robustness of our findings, we fitted three different models examining how Dyadic TSD Partner predicts SSA, considering variations by gender and stimuli gender:

1. Cumulative Link Mixed Model (CLMM) – `m3c_clmm` (for ordinal outcomes, using a probit link).
2. Generalized Linear Mixed Model (GLMM) with Poisson family – `m3c_poisson` (treating SSA as a count variable).
3. Linear Mixed Model (LMM) – `m3c_lmer` (treating SSA as a continuous variable).

```r
# (1) Cumulative Link Mixed Model (CLMM) - Ordinal model with probit link
m3c_clmm <- clmm(
  Subjective.sexual.arousal.factor ~ Dyadic.TSD.Partner * Gender * Relationship +
    (1 | Stimuli.code) + # Random intercept to account for variance across stimuli
    (1 | Participant), # Random intercept & slope for Relationship status
  data = dat_m3,
  link = "probit", # Use a probit link function for ordinal outcomes
  control = list(method = "nlminb") # Use 'nlminb' optimizer to improve convergence
)

# (2) Generalized Linear Mixed Model (GLMM) - Poisson regression for count data
m3c_poisson <- glmer(
  Subjective.sexual.arousal ~ Dyadic.TSD.Partner * Gender * Relationship +
    (1 | Stimuli.code) + # Random intercept for Stimuli
    (1 | Participant), # Random intercept & slope for Relationship status
  data = dat_m3,
  family = poisson # Use Poisson distribution for count-based SSA modeling
)

# (3) Linear Mixed Model (LMM) - Continuous approximation
m3c_lmer <- lmer(
  Subjective.sexual.arousal ~ Dyadic.TSD.Partner * Gender * Relationship +
    (1 | Stimuli.code) + # Random intercept for Stimuli
    (1 | Participant), # Random intercept & slope for Relationship status
  data = dat_m3,
  control = lmerControl(optimizer = "bobyqa") # Use 'bobyqa' optimizer for numerical stability
)
```

4.4.5.1.1 Table S34. ANOVA-type table of fixed effects (main effects and interactions) across the three fitted models

As shown in the table below, the pattern of significant effects remains consistent across all three models, except for the main effect of gender, which is not significant in the CLMM.

```r
# Generate an ANOVA-type comparison table for fixed effects across the three models
anova.comp(
  CLMMmod = m3c_clmm, # Cumulative Link Mixed Model (CLMM) - ordinal outcome
  GLMERmod = m3c_poisson, # Generalized Linear Mixed Model (GLMM) - Poisson outcome
  LMERmod = m3c_lmer, # Linear Mixed Model (LMM) - continuous outcome
  hypothesis = "3c" # Specify hypothesis being tested
)
```

Table S34. *Comparison of fixed effects across the three models for Hypothesis 3c: CLMM, GLMM (Poisson), and LMM.*

	CLMM			GLMER (Poisson)			LMM		
Effect	*df*	χ^2	*p*	*df*	χ^2	*p*	*df*	*F*	*p*
Dyadic TSD Partner	1	0.039	0.84	1	2.932	0.09	1, 311.9	3.163	0.08
Gender	1	2.276	0.13	1	2.719	0.1	1, 328.45	2.500	0.11
Relationship	1	0.080	0.78	1	0.062	0.8	1, 311.9	0.670	0.41
Dyadic TSD Partner × Gender	1	0.645	0.42	1	0.651	0.42	1, 311.98	1.153	0.28
Dyadic TSD Partner × Relationship	1	0.471	0.49	1	0.501	0.48	1, 311.9	1.374	0.24
Gender × Relationship	1	4.340	**0.0372**	1	7.102	**0.0077**	1, 311.9	8.505	**0.0038**
Dyadic TSD Partner × Gender × Relationship	1	3.905	**0.0481**	1	6.593	**0.0102**	1, 311.94	8.308	**0.0042**

Note: For CLMM and GLMER (Poisson) models, results are Analysis of Deviance (Type III Wald chi-square tests), while for LMM, results are from an Analysis of Variance (Type III ANOVA with Satterthwaite's method). Significant effects are in bold.

4.4.5.1.2 Figure S20: Model-based predictions for Hypothesis 3c. This figure presents model-based predictions of subjective sexual arousal as a function of Dyadic TSD Partner, across different relationship status and participant genders. The three subplots correspond to the three statistical models used for analysis: (a) Cumulative Link Mixed Model (CLMM), (b) Generalized Linear Mixed Model (GLMM, Poisson), and (c) Linear Mixed Model (LMM). Shaded areas represent 95% confidence intervals.

```
# CLMM Predictions
p_m3c_clmm <- emmeans(m3c_clmm, ~ Dyadic.TSD.Partner | Gender * Relationship,
  at = list(Dyadic.TSD.Partner = seq(0, 31, length.out = 100)), # Generate 100 points
  mode = "mean.class" # Compute predicted mean response categories
) |>
  as.data.frame() |> # Convert predictions to a dataframe
  ggplot(aes(
    x = Dyadic.TSD.Partner, y = mean.class,
    color = Relationship, fill = Relationship
  )) +
  geom_line(size = 1) + # Add predicted response line
  geom_ribbon(aes(ymin = asymp.LCL, ymax = asymp.UCL, fill = Relationship),
    alpha = 0.2, color = NA
  ) + # Add confidence interval
  scale_color_manual(values = color.Relationship) + # Apply custom colors
  scale_fill_manual(values = color.Relationship) +
  facet_wrap(~Gender, ncol = 1) + # Separate plots by gender
  labs(
    y = "Predicted Subjective Sexual Arousal", x = "Dyadic TSD Partner",
    title = "CLMM"
  ) +
  theme_tq() + # Apply theme
  theme(legend.position = "bottom") +
  ylim(c(0.3, 8)) # Set Y-axis limits

# Poisson GLMM Predictions
p_m3c_poisson <- emmeans(m3c_poisson, ~ Dyadic.TSD.Partner | Gender * Relationship,
  at = list(Dyadic.TSD.Partner = seq(0, 31, length.out = 100)), # Generate 100 points
  type = "response" # Compute response-scale predictions
) |>
  as.data.frame() |>
  ggplot(aes(
    x = Dyadic.TSD.Partner, y = rate,
    color = Relationship, fill = Relationship
  )) +
  geom_line(size = 1) +
  geom_ribbon(aes(ymin = asymp.LCL, ymax = asymp.UCL, fill = Relationship),
    alpha = 0.2, color = NA
  ) +
  scale_color_manual(values = color.Relationship) +
  scale_fill_manual(values = color.Relationship) +
  facet_wrap(~Gender, ncol = 1) +
  labs(y = "", x = "Dyadic TSD Partner", title = "GLMER (Poisson)") +
  theme_tq() +
  theme(legend.position = "bottom") +
  ylim(c(0.3, 8))

# LMM Predictions
p_m3c_lmer <- emmeans(m3c_lmer, ~ Dyadic.TSD.Partner | Gender * Relationship,
  at = list(Dyadic.TSD.Partner = seq(0, 31, length.out = 100)),
  type = "response"
```

```
) |>
  as.data.frame() |>
  ggplot(aes(
    x = Dyadic.TSD.Partner, y = emmean,
    color = Relationship, fill = Relationship
  )) +
  geom_line(size = 1) +
  geom_ribbon(aes(ymin = asymp.LCL, ymax = asymp.UCL, fill = Relationship),
    alpha = 0.2, color = NA
  ) +
  scale_color_manual(values = color.Relationship) +
  scale_fill_manual(values = color.Relationship) +
  facet_wrap(~Gender, ncol = 1) +
  labs(y = "", x = "Dyadic TSD Partner", title = "LMM") +
  theme_tq() +
  theme(legend.position = "bottom") +
  ylim(c(0.3, 8))

# Arrange Plots into a Single Figure
p_robu_m3c <- ggarrange(p_m3c_clmm, p_m3c_poisson, p_m3c_lmer, # Combine plots side by side
  common.legend = TRUE, # Share legend across plots
  labels = "auto", # Automatically label subfigures (a, b, c)
  legend = "bottom",
  nrow = 1 # Arrange in a single row
)

# Display the combined figure
p_robu_m3c
```

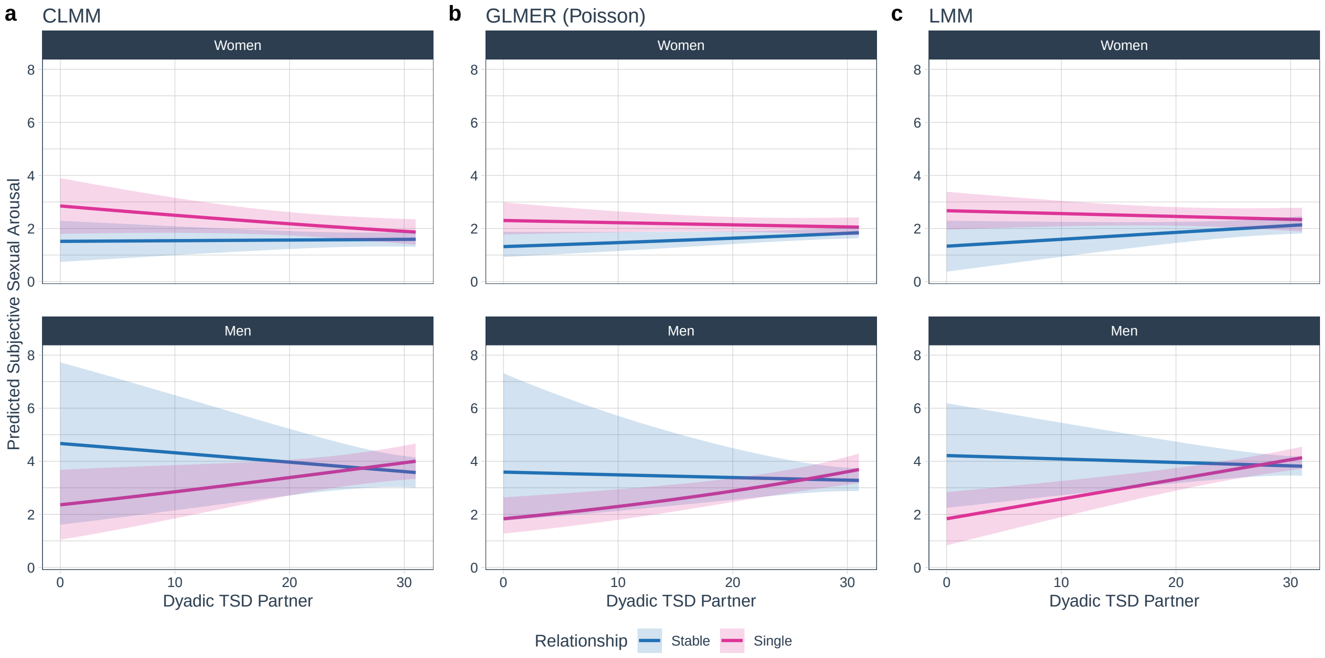

Figure S20. Predicted subjective sexual arousal as a function of Dyadic TSD Partner, modeled using three statistical approaches: **(a)** Cumulative Link Mixed Model (CLMM); **(b)** Generalized Linear Mixed Model (GLMM) with a Poisson family; **(c)** Linear Mixed Model (LMM). Lines represent predicted values, and shaded areas indicate 95% confidence intervals. The models include participant gender and relationship status as key factors.

4.4.5.2 Final Model: Effects of Dyadic TSD Partner on SSA Across Gender and Stimuli Gender

Given the apparent robustness of most results across models (CLMM, GLMER and LMM; Table S34, Fig. S20), we test the predictions of the hypothesis from the LMM (`m3c_lmer`).

4.4.5.2.1 Table S35. ANOVA-type table for the interaction between `Relationship type`, and `Gender`

This tables summarizes the results of the model.

```
# Generate ANOVA-type table for the final LMM model
# This summarizes the effects of Dyadic TSD Partner on SSA across Gender and Stimuli Gender
anova.sig.lmer(
  model = m3c_lmer, # Use LMM as the final model
  custom_caption = "Effects of Dyadic TSD Partner on SSA Across Gender and Stimuli Gender"
)
```

Table S35. *Effects of Dyadic TSD Partner on SSA Across Gender and Stimuli Gender*

Effect	*df*	*F*	*p*	ϵ_p^2
Dyadic TSD Partner	1, 311.9	3.16	0.08	0.0069
Gender	1, 328.45	2.50	0.11	0.0045
Relationship	1, 311.9	0.67	0.41	< 0.0001
Dyadic TSD Partner × Gender	1, 311.98	1.15	0.28	< 0.001
Dyadic TSD Partner × Relationship	1, 311.9	1.37	0.24	0.0012
Gender × Relationship	1, 311.9	8.51	**0.0038**	0.0234
Dyadic TSD Partner × Gender × Relationship	1, 311.94	8.31	**0.0042**	0.0228

Note: Results are Type III ANOVA. $R^2_{conditional} = 0.719$, $R^2_{marginal} = 0.182$. As effect size, we report partial epsilon squared (ϵ_p^2), a less biased estimate than η^2 (see Albers and Lakens, 2018). Significant effects are in bold.

4.4.5.2.2 *Post-hoc* tests

To test the hypothesis, which predicted that there would be different relationship between SSA and Dyadic TSD Partner, and that this association differ between men and women depending on the gender of stimuli, we used simple slope analysis.

```
# Perform simple slope analysis to examine how Dyadic TSD Partner predicts SSA
# The model includes relationship status and gender as moderators
slop.m3c_lmer <- sim_slopes(
  m3c_lmer,
  pred = Dyadic.TSD.Partner, # Predictor: Dyadic TSD Partner
  modx = Relationship, # First moderator: Relationship status
  mod2 = Gender, # Second moderator: Gender
  confint = TRUE # Compute confidence intervals
)

# Bind slopes for women and men into a single table, adding Gender as a column
slop.m3c_lmer.tab <- bind_rows(
  slop.m3c_lmer$slopes[[1]] |> mutate(Gender = "Women"),
  slop.m3c_lmer$slopes[[2]] |> mutate(Gender = "Men")
) |>
  # Recode Gender variable to English labels
  mutate(Gender = recode_factor(Gender, Femenino = "Women", Masculino = "Men")) |>
  # Select relevant columns (reordering for clarity)
  select(8, 1:2, 4:7) |>
  # Convert specific columns to numeric format
  mutate(across(3:7, as.numeric)) |>
  # Round coefficients and confidence intervals to 2 decimal places
  mutate(across(3:6, round, 2)) |>
  # Add significance stars based on p-values
```

```
  mutate(sig = pval.stars(p)) |>
  # Rename columns for clarity
  rename("Relationship" = "Value of Relationship") |>
  rename(Coefficient = Est.)

# Create formatted output table
slop.m3c_lmer.tab[, -c(1, 8)] |> # Exclude unnecessary columns
  mutate(p = pval.lev(p)) |> # Format p-values
  kable(
    booktabs = TRUE,
    align = c("l", rep("c", 5)), # Align columns
    caption = "Slope for Dyadic TSD Partner on Subjective sexual arousal
               by stimuli gender and gender",
    linesep = "",
    col.names = c("Relationship status", "$B$", "$2.5\\% CI$", "$97.5\\% CI$", "$t$", "$p$"),
    escape = FALSE
  ) |>
  # Apply table styling
  kable_styling(latex_options = c("HOLD_position")) |>
  # Group rows by gender for better readability
  pack_rows("Gender: Women", start_row = 1, end_row = 2,
            bold = FALSE, background = "lightgray") |>
  pack_rows("Gender: Men", start_row = 3, end_row = 4,
            bold = FALSE, background = "lightgray") |>
  # Add a footnote for coefficient interpretation
  footnote(
    general = "$B$ are unstandardized coefficients. No intercept is reported
               as continuous predictors were centered and are dependent on this sample.",
    threeparttable = TRUE,
    footnote_as_chunk = TRUE,
    escape = FALSE
  )
```

Table S36. *Slope for Dyadic TSD Partner on Subjective sexual arousal by stimuli gender and gender*

Relationship status	B	$2.5\% CI$	$97.5\% CI$	t	p
Gender: Women					
Stable	0.03	-0.01	0.06	1.54	0.12
Single	-0.01	-0.04	0.02	-0.73	0.47
Gender: Men					
Stable	-0.01	-0.07	0.05	-0.41	0.68
Single	0.07	0.04	0.11	4.02	$<$ **0.0001**

Note: B are unstandardized coefficients. No intercept is reported as continuous predictors were centered and are dependent on this sample.

4.4.5.3 Figure S21. Subjective sexual arousal to erotic stimuli: Main effects and interactions

This figure summarizes the results of hypothesis 3c.

```
# Generate figure to visualize interaction between Relationship status and Gender
p_m3c.fin <- p_m3c_lmer +
  # Set axis labels (removing title for a cleaner look)
  labs(title = "", y = "Predicted Subjective Sexual Arousal") +
  # Facet by Gender, displaying two columns
  facet_wrap(~Gender, ncol = 2) +
```

```
  # Add text labels for regression slopes
  geom_text(
    data = slop.m3c_lmer.tab |> mutate(Dyadic.TSD.Partner = 2),
    mapping = aes(
      x = min(Dyadic.TSD.Partner), y = Inf,
      label = paste(
        "B = ", Coefficient, ", IC 95%[", `2.5%`, ", ", `97.5%`, "], p",
        ifelse(
          grepl("<", pe2.lev(p)), pe2.lev(p), paste0(" = ", pe2.lev(p))
        ),
        ifelse(is.na(sig), "", sig) # Add significance stars if applicable
      ),
      vjust = 2 + as.numeric(as.factor(Relationship)) * 2 # Adjust vertical spacing
    ),
    hjust = -0.1, # Align text slightly to the left
    # size = 3,  # Uncomment to control text size if needed
    show.legend = FALSE # Hide legend for text labels
  ) +
  # Move legend to the bottom
  theme(legend.position = "bottom")

# Display the final plot
p_m3c.fin
```

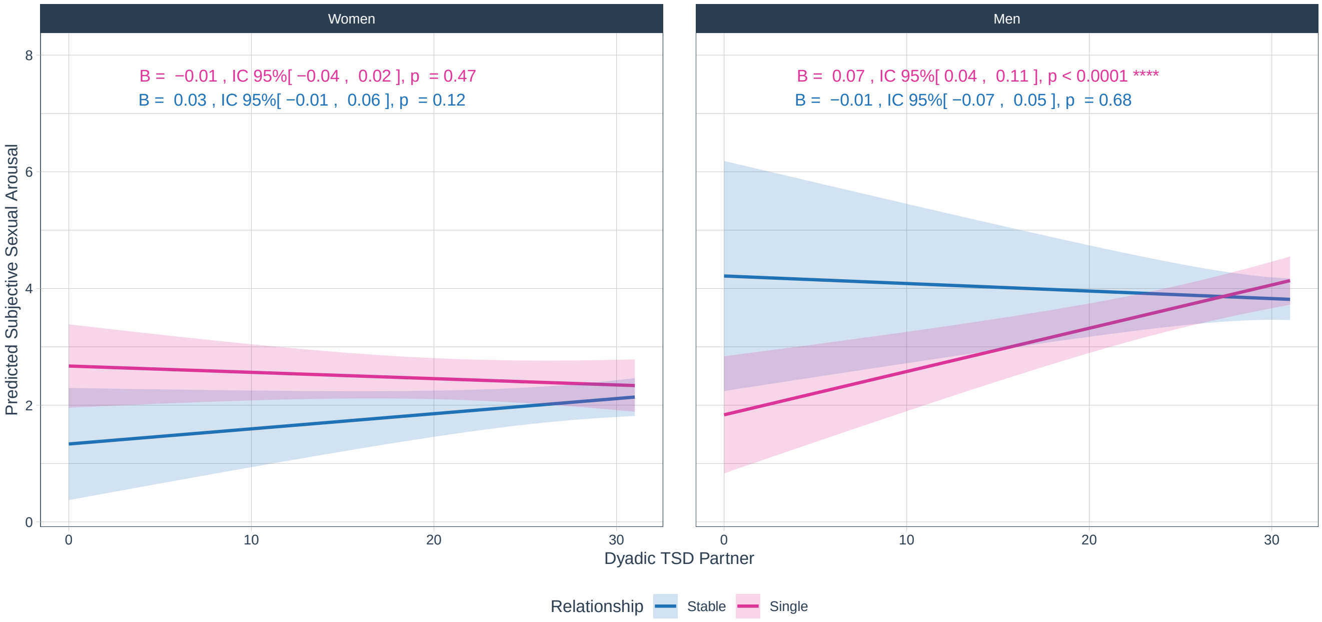

Figure S21. Predicted subjective sexual arousal as a function of Dyadic TSD Partner, modeled using aLinear Mixed Model (LMM). Lines represent predicted values, and shaded areas indicate 95% confidence intervals. The model include participant gender and relationship status as key factors.

---

5 Final figures and tables

Figures and tables included in the main document.

5.1 Table 1. Hypothesis 1

ANOVA-type table for the interaction between `Relationship type`, and `Gender` for the three final models for hypothesis 1.

```
# Generate ANOVA-type table for the interaction between Relationship type and Gender
# across the three final models for Hypothesis 1
reduce(
  list(
    # Compute ANOVA summary for Solitary TSD
    bind_cols(
      anova_summary(Anova(m1a_norm, type = 3)),
      epsilon_squared(m1a_norm)
    ) |>
      unite(col = "df", DFn:DFd, sep = ", ") |> # Combine degrees of freedom into one column
      select(Effect, df, F, p, Epsilon2_partial) |> # Select relevant columns
      mutate(
        p = pval.lev(p), # Format p-values
        Epsilon2_partial = pe2.lev(Epsilon2_partial) # Format epsilon squared values
      ),
    # Compute ANOVA summary for Dyadic TSD Attractive Person
    bind_cols(
      anova_summary(Anova(m1b_norm, type = 3)),
      epsilon_squared(m1b_norm)
    ) |>
      unite(col = "df", DFn:DFd, sep = ", ") |>
      select(Effect, df, F, p, Epsilon2_partial) |>
      mutate(
        p = pval.lev(p),
        Epsilon2_partial = pe2.lev(Epsilon2_partial)
      ),
    # Compute ANOVA summary for Dyadic TSD Partner
    bind_cols(
      anova_summary(Anova(m1c_norm, type = 3)),
      epsilon_squared(m1c_norm)
    ) |>
      unite(col = "df", DFn:DFd, sep = ", ") |>
      select(Effect, df, F, p, Epsilon2_partial) |>
      mutate(
        p = pval.lev(p),
        Epsilon2_partial = pe2.lev(Epsilon2_partial)
      )
  ),
  full_join, # Merge results by Effect
  by = "Effect"
) |>
  mutate_at("Effect", str_replace_all, ":", " × ") |> # Replace ":" with "×" for readability
  kable(
    digits = 2,
    booktabs = TRUE,
    align = c("l", rep("c", 12)),
    linesep = "",
    caption = "Effects of relationship type and gender on TSD dimensions",
    col.names = c("Effect", rep(c("$df$", "$F$", "$p$", "$\\epsilon^2_p$"), 3)),
    escape = FALSE
  ) |>
  kable_styling(latex_options = c("HOLD_position", "scale_down")) |>
```

```
  add_header_above(c(
    " " = 1, # Empty column
    "Solitary TSD" = 4,
    "Dyadic TSD Attractive Person" = 4,
    "Dyadic TSD Partner" = 4
  )) |>
  footnote(
    general = paste0(
      "Sexual desire was transformed using ordered quantile normalization ",
      "(\\\\cite{petersonOrderedQuantileNormalization2020a}). Results are type III ANOVA. ",
      "Solitary TSD: $R^2$ = ", round(r2(m1a_norm)$R2, 3),
      ", $R^2_{adjusted}$ = ", round(r2(m1a_norm)$R2_adjusted, 3),
      "; Dyadic TSD Attractive Person: $R^2$ = ", round(r2(m1b_norm)$R2, 3),
      ", $R^2_{adjusted}$ = ", round(r2(m1b_norm)$R2_adjusted, 3),
      "; Dyadic TSD - Partner: $R^2$ = ", round(r2(m1c_norm)$R2, 3),
      ", $R^2_{adjusted}$ = ", round(r2(m1c_norm)$R2_adjusted, 3),
      ". Gender = participants gender (women, men); ",
      "Relationship = relationship type (stable, single). ",
      "As effect size, we report partial epsilon squared ($\\\\epsilon^2_p$), which provides ",
      "a less biased estimate than $\\\\eta^2$ (see \\\\cite{albersWhenPowerAnalyses2018}). ",
      "Significant effects are in bold."
    ),
    escape = FALSE,
    threeparttable = TRUE,
    footnote_as_chunk = TRUE
  )
```

Table 1. *Effects of relationship type and gender on TSD dimensions*

	Solitary TSD				Dyadic TSD Attractive Person				Dyadic TSD Partner			
Effect	*df*	*F*	*p*	ϵ_p^2	*df*	*F*	*p*	ϵ_p^2	*df*	*F*	*p*	ϵ_p^2
Gender	1, 319	22.42	**< 0.0001**	0.06	1, 319	29.85	**< 0.0001**	0.09	1, 316	15.49	**< 0.001**	0.0365
Relationship	1, 319	14.07	**< 0.001**	0.03	1, 319	8.20	**0.004**	0.03	1, 316	31.60	**< 0.0001**	0.09
Gender × Relationship	1, 319	4.23	**0.04**	0.01	1, 319	1.73	0.19	0.00	1, 316	0.00	0.98	< 0.0001

Note: Sexual desire was transformed using ordered quantile normalization (Peterson and Cavanaugh, 2020). Results are type III ANOVA. Solitary TSD: $R^2 = 0.103$, $R^2_{adjusted} = 0.095$; Dyadic TSD Attractive Person: $R^2 = 0.122$, $R^2_{adjusted} = 0.114$; Dyadic TSD - Partner: $R^2 = 0.125$, $R^2_{adjusted} = 0.117$. Gender = participants gender (women, men); Relationship = relationship type (stable, single). As effect size, we report partial epsilon squared (ϵ^2_p), which provides a less biased estimate than η^2 (see Albers and Lakens, 2018). Significant effects are in bold.

5.2 Figure 1. Hypothesis 1

Estimated marginal means for the interaction between `Relationship type`, and `Gender` for the three final models for hypothesis 1.

```
# Create a combined figure for Hypothesis 1
# Estimated marginal means for the interaction between Relationship type and Gender
# across the three final models for sexual desire dimensions
ggarrange(
  h1a3, h1b3, h1c3, # Arrange the three subplots (a: Solitary TSD, b: Dyadic TSD Attractive Person,
  # c: Dyadic TSD Partner)
  common.legend = TRUE, # Use a shared legend across plots
  legend = "bottom", # Position legend at the bottom
  labels = "auto", # Automatically label subplots (a, b, c)
  nrow = 1 # Arrange all plots in a single row
)
```

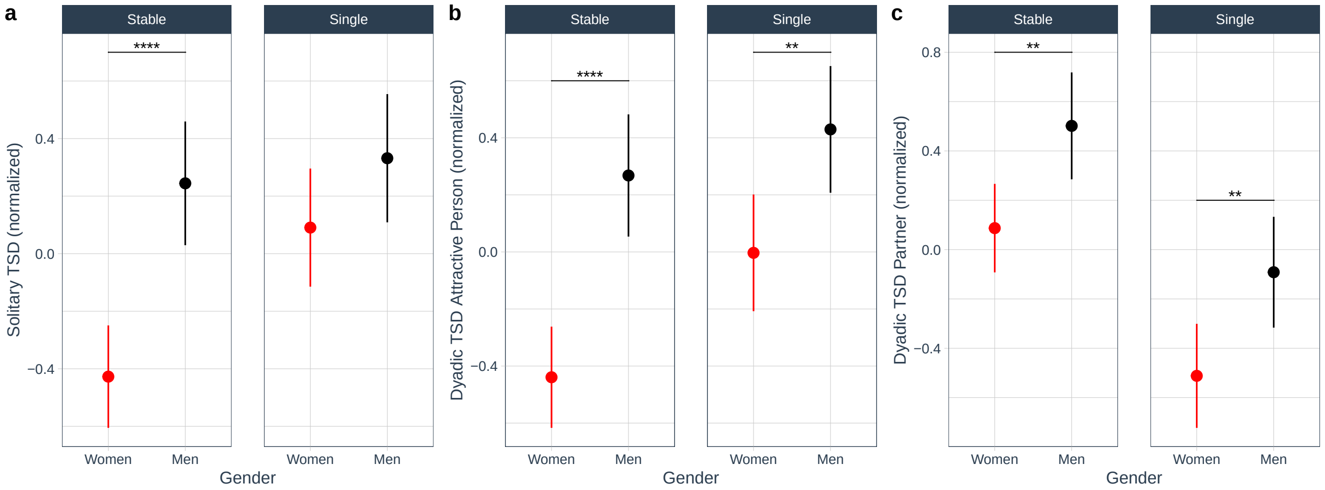

Figure 1. Effects of gender and relationship type on dimensions of Trait Sexual Desire (TSD). All dimensions of TSD were transformed using ordered quantile normalization (Peterson & Cavanaugh, 2020). **(a)** Solitary TSD; **(b)** Dyadic TSD Attractive Person; **(c)** Dyadic TSD Partner. Dots and bars represent estimated marginal means and 95% CI. In all cases, significant effects are represented with lines and stars: $*p < 0.05$, $**p < 0.01$, $***p < 0.001$, $****p < 0.0001$.

5.3 Table 2. Hypothesis 2

ANOVA-type table for the interaction between `Relationship type`, and `Gender` for the three final models for hypothesis 2.

```
# Generate an ANOVA-type table summarizing the effects of relationship type and gender
# on the three final models for Hypothesis 2 (Solitary TSD, Dyadic TSD Attractive Person,
# Dyadic TSD Partner)
bind_rows(
  # ANOVA results for Solitary TSD
  bind_cols(
    anova(m2a_lmer), # Perform ANOVA on the LMM for Solitary TSD
    epsilon_squared(m2a_lmer) # Compute partial epsilon squared effect sizes
  ) |>
    mutate(DenDF = round(DenDF, 2)) |> # Round denominator degrees of freedom
    unite(col = "df", NumDF:DenDF, sep = ", ") |> # Combine NumDF and DenDF into "df" column
    rownames_to_column(var = "Effect") |> # Convert row names to a column
    rename("F" = "F value", "p" = "Pr(>F)") |> # Rename columns for clarity
    select(Effect, df, F, p, Epsilon2_partial) |> # Select relevant columns
    mutate(p = pval.lev(p), Epsilon2_partial = pe2.lev(Epsilon2_partial)), # Format p-values
  # ANOVA results for Dyadic TSD Attractive Person
  bind_cols(
    anova(m2b_lmer),
    epsilon_squared(m2b_lmer)
  ) |>
    mutate(DenDF = round(DenDF, 2)) |>
    unite(col = "df", NumDF:DenDF, sep = ", ") |>
    rownames_to_column(var = "Effect") |>
    rename("F" = "F value", "p" = "Pr(>F)") |>
    select(Effect, df, F, p, Epsilon2_partial) |>
    mutate(p = pval.lev(p), Epsilon2_partial = pe2.lev(Epsilon2_partial)),
  # ANOVA results for Dyadic TSD Partner
  bind_cols(
    anova(m2c_lmer),
    epsilon_squared(m2c_lmer)
```

```
  ) |>
    mutate(DenDF = round(DenDF, 2)) |>
    unite(col = "df", NumDF:DenDF, sep = ", ") |>
    rownames_to_column(var = "Effect") |>
    rename("F" = "F value", "p" = "Pr(>F)") |>
    select(Effect, df, F, p, Epsilon2_partial) |>
    mutate(p = pval.lev(p), Epsilon2_partial = pe2.lev(Epsilon2_partial))
) |>
  # Format effect names for readability
  mutate(Effect = str_replace_all(Effect, "\\.", " ")) |>
  mutate(Effect = str_replace_all(Effect, ":", " × ")) |>
  # Generate a formatted table using `kable`
  kable(
    digits = 2, # Round values to 2 decimal places
    booktabs = TRUE, # Use LaTeX booktabs for better formatting
    align = c("l", rep("c", 4)), # Align columns
    linesep = "", # No extra lines
    caption = "Effects of TSD dimensions on SSA Across Gender and Stimuli Gender",
    col.names = c("Effect", "$df$", "$F$", "$p$", "$\\epsilon^2_p$"), # Column headers
    escape = FALSE # Allow LaTeX formatting
  ) |>
  kable_styling(latex_options = c("HOLD_position", "scale_down")) |> # LaTeX table styling
  # Group the table into three sections
  pack_rows("Solitary TSD", 1, 7,
            bold = FALSE, background = "lightgray") |>
  pack_rows("Dyadic TSD Attractive Person", 8, 14,
            bold = FALSE, background = "lightgray") |>
  pack_rows("Dyadic TSD Partner", 15, 21,
            bold = FALSE, background = "lightgray") |>
  # Add footnotes with additional details
  footnote(
    general = paste0(
      "Results are type III ANOVA. Solitary TSD: ",
      "$R^2_{conditional}$ = ", round(r2_nakagawa(m2a_lmer)$R2_conditional, 3),
      ", $R^2_{marginal}$ = ", round(r2_nakagawa(m2a_lmer)$R2_marginal, 3),
      "; Dyadic TSD Attractive Person: ",
      "$R^2_{conditional}$ = ", round(r2_nakagawa(m2b_lmer)$R2_conditional, 3),
      ", $R^2_{marginal}$ = ", round(r2_nakagawa(m2b_lmer)$R2_marginal, 3),
      "; Dyadic TSD Partner: ",
      "$R^2_{conditional}$ = ", round(r2_nakagawa(m2c_lmer)$R2_conditional, 3),
      ", $R^2_{marginal}$ = ", round(r2_nakagawa(m2c_lmer)$R2_marginal, 3),
      ". Gender = participant gender (women, men); Stimuli gender = gender of stimuli (male, female).
      Partial epsilon squared ($\\\\epsilon^2_p$) is used as an effect size, as it provides
      a less biased estimate than $\\\\eta^2$ (see \\\\cite{albersWhenPowerAnalyses2018}).
      Significant effects are in bold."
    ),
    escape = FALSE, # Allow LaTeX formatting
    threeparttable = TRUE,
    footnote_as_chunk = TRUE
  )
```

Table 2. *Effects of TSD dimensions on SSA Across Gender and Stimuli Gender*

Effect	*df*	*F*	*p*	ϵ_p^2
Solitary TSD				
Solitary TSD	1, 319	17.46	**< 0.0001**	0.0489
Gender	1, 319	8.84	**0.0032**	0.0239
Stimuli gender	1, 369.21	24.71	**< 0.0001**	0.06
Solitary TSD × Gender	1, 319	0.85	0.36	< 0.0001
Solitary TSD × Stimuli gender	1, 319	0.02	0.88	< 0.0001
Gender × Stimuli gender	1, 319	74.79	**< 0.0001**	0.19
Solitary TSD × Gender × Stimuli gender	1, 319	1.78	0.18	0.0024
Dyadic TSD Attractive Person				
Dyadic TSD Attractive Person	1, 319	48.49	**< 0.0001**	0.13
Gender	1, 319	1.45	0.23	0.0014
Stimuli gender	1, 373.93	2.69	0.1	0.0045
Dyadic TSD Attractive Person × Gender	1, 319	0.53	0.47	< 0.0001
Dyadic TSD Attractive Person × Stimuli gender	1, 319	15.43	**< 0.001**	0.0431
Gender × Stimuli gender	1, 319	27.44	**< 0.0001**	0.08
Dyadic TSD Attractive Person × Gender × Stimuli gender	1, 319	29.69	**< 0.0001**	0.08
Dyadic TSD Partner				
Dyadic TSD Partner	1, 316	6.59	**0.0107**	0.0173
Gender	1, 316	0.03	0.85	< 0.0001
Stimuli gender	1, 344.42	0.99	0.32	< 0.0001
Dyadic TSD Partner × Gender	1, 316	3.97	**0.0472**	0.0093
Dyadic TSD Partner × Stimuli gender	1, 316	8.46	**0.0039**	0.023
Gender × Stimuli gender	1, 316	20.55	**< 0.0001**	0.06
Dyadic TSD Partner × Gender × Stimuli gender	1, 316	5.70	**0.0176**	0.0146

Note: Results are type III ANOVA. Solitary TSD: $R^2_{conditional} = 0.745$, $R^2_{marginal} = 0.335$; Dyadic TSD Attractive Person: $R^2_{conditional} = 0.745$, $R^2_{marginal} = 0.367$; Dyadic TSD Partner: $R^2_{conditional} = 0.745$, $R^2_{marginal} = 0.329$. Gender = participant gender (women, men); Stimuli gender = gender of stimuli (male, female). Partial epsilon squared (ϵ_p^2) is used as an effect size, as it provides a less biased estimate than η^2 (see Albers and Lakens, 2018). Significant effects are in bold.

5.4 Figure 2. Hypothesis 2

Simple slopes for the interaction between dimensions of sexual desire and `Stimuli Gender`, by gender, for the three final models for hypothesis 2.

```
# Generate a combined figure showing the interaction between sexual desire dimensions
# and stimuli gender, grouped by gender, for the final models in Hypothesis 2
ggarrange(
  p_m2a.fin, # Solitary TSD simple slopes plot
  p_m2b.fin, # Dyadic TSD Attractive Person simple slopes plot
  p_m2c.fin, # Dyadic TSD Partner simple slopes plot
  common.legend = TRUE, # Use a shared legend across all plots
  legend = "bottom", # Position the legend at the bottom
  labels = "auto", # Automatically label subfigures (a, b, c)
  nrow = 3, # Arrange plots in three rows
  ncol = 1 # Use a single column layout
)
```

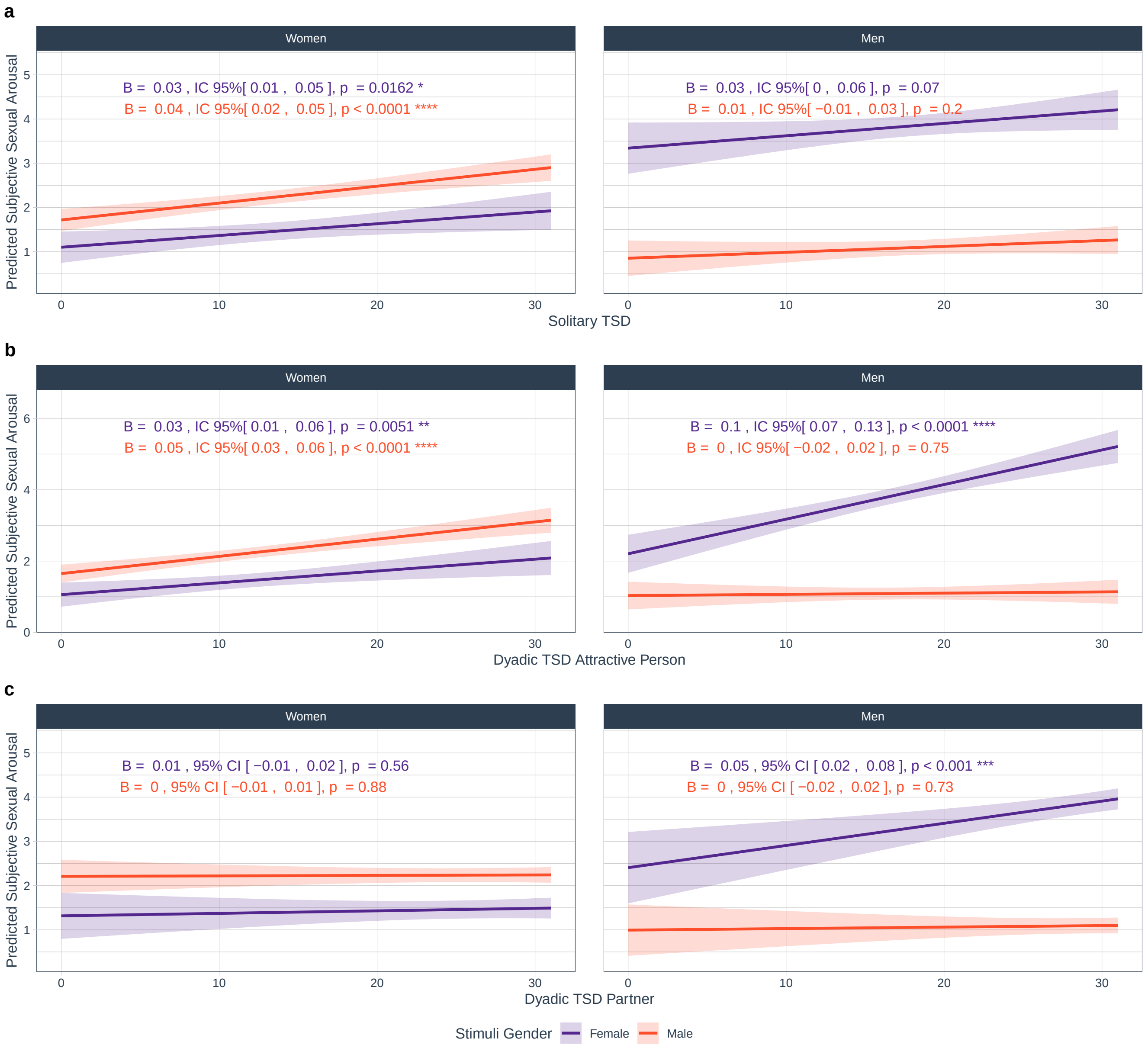

Figure 2. Slopes of Trait Sexual Desire (TSD) dimensions on sexual arousal, by gender and stimuli gender. **(a)** Solitary TSD; **(b)** Dyadic TSD Attractive Person; **(c)** Dyadic TSD Partner. Lines represent simple slopes and 95% CI. Significant effects are represented with stars alongside slope details: $*p < 0.05$, $**p < 0.01$, $***p < 0.001$, $****p < 0.0001$.

5.5 Table 3. Hypothesis 3

ANOVA-type table for the interaction between `Relationship type`, and `Gender` for the three final models for hypothesis 3.

```
# Generate an ANOVA-type table summarizing the interaction between
# Relationship Type and Gender for Hypothesis 3 models
bind_rows(
  # Model 3a: Solitary TSD
  bind_cols(anova(m3a_lmer), epsilon_squared(m3a_lmer)) |>
    mutate(DenDF = round(DenDF, 2)) |> # Round denominator degrees of freedom
    unite(col = "df", NumDF:DenDF, sep = ", ") |> # Combine NumDF and DenDF into one column
    rownames_to_column(var = "Effect") |> # Convert row names to a column
```

```
    rename("F" = "F value", "p" = "Pr(>F)") |> # Rename columns for clarity
    select(Effect, df, F, p, Epsilon2_partial) |> # Keep relevant columns
    mutate(p = pval.lev(p), Epsilon2_partial = pe2.lev(Epsilon2_partial)),
  # Model 3b: Dyadic TSD Attractive Person
  bind_cols(anova(m3b_lmer), epsilon_squared(m3b_lmer)) |>
    mutate(DenDF = round(DenDF, 2)) |>
    unite(col = "df", NumDF:DenDF, sep = ", ") |>
    rownames_to_column(var = "Effect") |>
    rename("F" = "F value", "p" = "Pr(>F)") |>
    select(Effect, df, F, p, Epsilon2_partial) |>
    mutate(p = pval.lev(p), Epsilon2_partial = pe2.lev(Epsilon2_partial)),
  # Model 3c: Dyadic TSD Partner
  bind_cols(anova(m3c_lmer), epsilon_squared(m3c_lmer)) |>
    mutate(DenDF = round(DenDF, 2)) |>
    unite(col = "df", NumDF:DenDF, sep = ", ") |>
    rownames_to_column(var = "Effect") |>
    rename("F" = "F value", "p" = "Pr(>F)") |>
    select(Effect, df, F, p, Epsilon2_partial) |>
    mutate(p = pval.lev(p), Epsilon2_partial = pe2.lev(Epsilon2_partial))
) |>
  mutate(Effect = str_replace_all(Effect, "\\.", " ")) |> # Replace dots with spaces
  mutate(Effect = str_replace_all(Effect, ":", " × ")) |> # Replace colons with ×
  # Format table output
  kable(
    digits = 2, booktabs = TRUE, align = c("l", rep("c", 4)),
    linesep = "", caption = "Effects of TSD dimensions on SSA across Gender and
    Relationship Status",
    col.names = c("Effect", "$df$", "$F$", "$p$", "$\\epsilon^2_p$"), escape = FALSE
  ) |>
  kable_styling(latex_options = c("HOLD_position", "scale_down")) |>
  # Add section grouping for each model
  pack_rows("Solitary TSD", 1, 7,
            bold = FALSE, background = "lightgray") |>
  pack_rows("Dyadic TSD Attractive Person", 8, 14,
            bold = FALSE, background = "lightgray") |>
  pack_rows("Dyadic TSD Partner", 15, 21,
            bold = FALSE, background = "lightgray") |>
  # Add footnote with additional model details
  footnote(
    general = paste0(
      "Results are type III ANOVA. ",
      "Solitary TSD: $R^2_{conditional}$ = ", round(r2_nakagawa(m3a_lmer)$R2_conditional, 3),
      ", $R^2_{marginal}$ = ", round(r2_nakagawa(m3a_lmer)$R2_marginal, 3),
      "; Dyadic TSD Attractive Person: $R^2_{conditional}$ = ",
      round(r2_nakagawa(m3b_lmer)$R2_conditional, 3),
      ", $R^2_{marginal}$ = ", round(r2_nakagawa(m3b_lmer)$R2_marginal, 3),
      "; Dyadic TSD - Partner: $R^2_{conditional}$ = ",
      round(r2_nakagawa(m3c_lmer)$R2_conditional, 3),
      ", $R^2_{marginal}$ = ", round(r2_nakagawa(m3c_lmer)$R2_marginal, 3),
      ". Gender = participants' gender (women, men); Relationship = relationship type
      (stable, single). As effect size, we report partial epsilon squared ($\\\\epsilon^2_p$),
      which provides a less biased estimate than $\\\\eta^2$
      (see \\\\cite{albersWhenPowerAnalyses2018}). Significant effects are in bold."
    ),
    escape = FALSE, threeparttable = TRUE, footnote_as_chunk = TRUE
  )
```

Table 3. *Effects of TSD dimensions on SSA across Gender and Relationship Status*

Effect	*df*	*F*	*p*	ϵ_p^2
Solitary TSD				
Solitary TSD	1, 315	6.88	**0.0091**	0.0183
Gender	1, 355.34	14.10	**< 0.001**	0.0355
Relationship	1, 314.95	0.34	0.56	< 0.0001
Solitary TSD × Gender	1, 315.23	0.07	0.79	< 0.0001
Solitary TSD × Relationship	1, 314.95	0.53	0.47	< 0.0001
Gender × Relationship	1, 314.95	2.95	0.09	0.0061
Solitary TSD × Gender × Relationship	1, 315.08	2.02	0.16	0.0032
Dyadic TSD Attractive Person				
Dyadic TSD Attractive Person	1, 315.21	46.80	**< 0.0001**	0.13
Gender	1, 354.77	1.21	0.27	< 0.001
Relationship	1, 315.16	0.13	0.72	< 0.0001
Dyadic TSD Attractive Person × Gender	1, 314.97	7.06	**0.0083**	0.0188
Dyadic TSD Attractive Person × Relationship	1, 315.21	0.08	0.77	< 0.0001
Gender × Relationship	1, 315.06	0.00	0.97	< 0.0001
Dyadic TSD Attractive Person × Gender × Relationship	1, 314.97	0.34	0.56	< 0.0001
Dyadic TSD Partner				
Dyadic TSD Partner	1, 311.9	3.16	0.08	0.0069
Gender	1, 328.45	2.50	0.11	0.0045
Relationship	1, 311.9	0.67	0.41	< 0.0001
Dyadic TSD Partner × Gender	1, 311.98	1.15	0.28	< 0.001
Dyadic TSD Partner × Relationship	1, 311.9	1.37	0.24	0.0012
Gender × Relationship	1, 311.9	8.51	**0.0038**	0.0234
Dyadic TSD Partner × Gender × Relationship	1, 311.94	8.31	**0.0042**	0.0228

Note: Results are type III ANOVA. Solitary TSD: $R^2_{conditional} = 0.72$, $R^2_{marginal} = 0.171$; Dyadic TSD Attractive Person: $R^2_{conditional} = 0.719$, $R^2_{marginal} = 0.225$; Dyadic TSD - Partner: $R^2_{conditional} = 0.719$, $R^2_{marginal} = 0.182$. Gender = participants' gender (women, men); Relationship = relationship type (stable, single). As effect size, we report partial epsilon squared (ϵ_p^2), which provides a less biased estimate than η^2 (see Albers and Lakens, 2018). Significant effects are in bold.

5.6 Figure 3. Hypothesis 3

Simple slopes for the interaction between dimensions of sexual desire and `Stimuli Gender`, by gender, for the three final models for hypothesis 3.

```
# Generate a combined figure for Hypothesis 3
# This figure shows simple slopes for sexual desire dimensions on sexual arousal
ggarrange(
  p_m3a.fin, p_m3b.fin, p_m3c.fin, # Arrange the three model plots
  common.legend = TRUE, # Use a shared legend for all subplots
  legend = "bottom", # Position the legend below the plots
  labels = "auto", # Automatically label subplots as (a), (b), (c)
  nrow = 3, # Arrange in three rows
  ncol = 1 # Single column layout
)
```

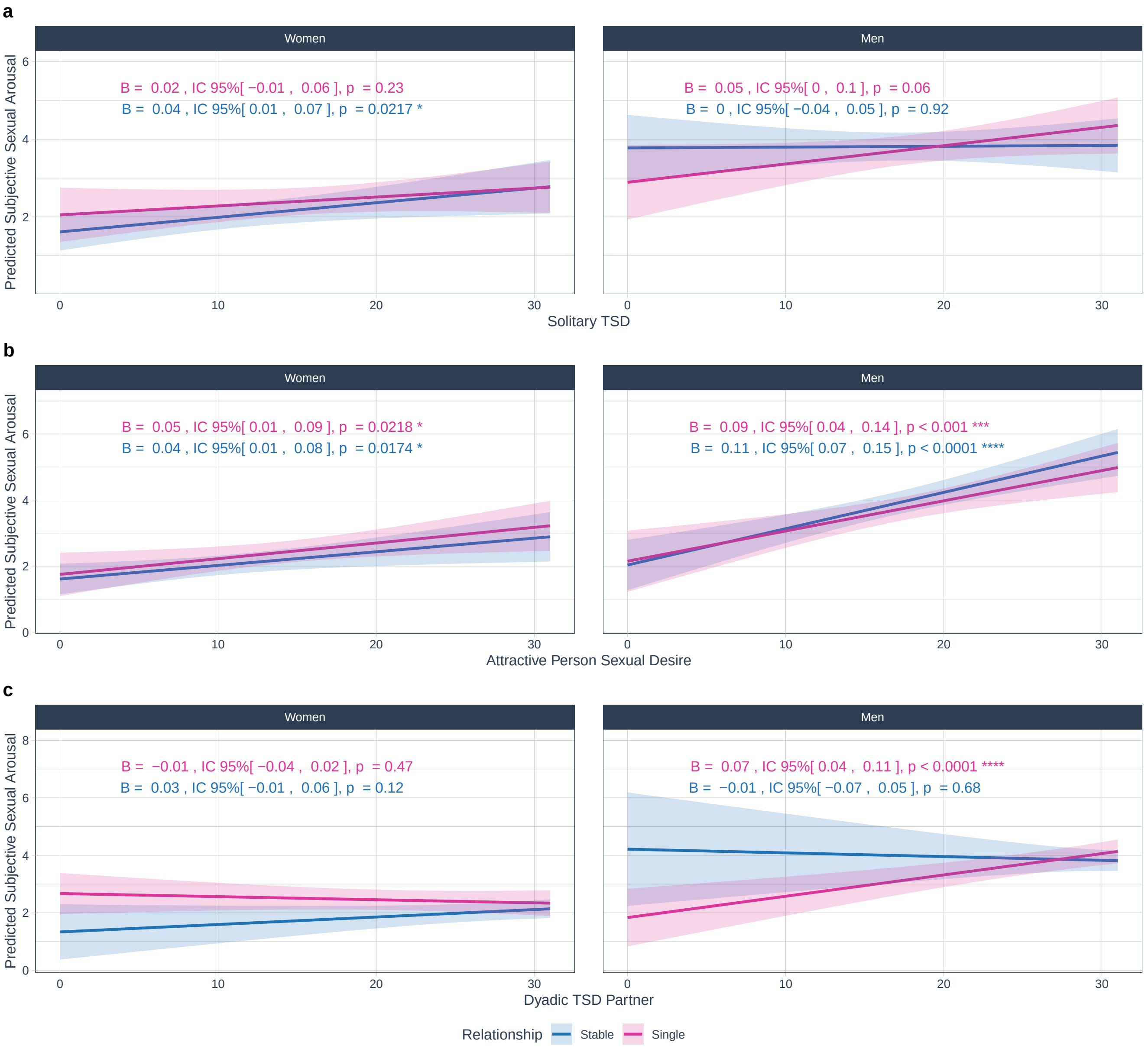

Figure 3. Slopes of Trait Sexual Desire (TSD) dimensions on sexual arousal, by gender and relationship status. **(a)** Solitary TSD; **(b)** Dyadic TSD Attractive Person; **(c)** Dyadic TSD Partner. Lines represent simple slopes and 95% CI. Significant effects are represented with stars alongside slope details: *$p < 0.05$, **$p < 0.01$, ***$p < 0.001$, ****$p < 0.0001$.

---

6 Session info (for reproducibility)

```r
# Display session information for reproducibility
# - Uses `pander()` for better formatting
# - `locale = FALSE` to exclude locale-specific info (reduces clutter)
library(pander)
pander(sessionInfo(), locale = FALSE)
```

R version 4.5.1 (2025-06-13)

Platform: x86_64-pc-linux-gnu

attached base packages: *stats*, *graphics*, *grDevices*, *utils*, *datasets*, *methods* and *base*

other attached packages: *pander(v.0.6.6)*, *Hmisc(v.5.2-3)*, *lubridate(v.1.9.4)*, *forcats(v.1.0.0)*, *stringr(v.1.5.1)*, *dplyr(v.1.1.4)*, *purrr(v.1.1.0)*, *readr(v.2.1.5)*, *tidyr(v.1.3.1)*, *tibble(v.3.3.0)*, *tidyverse(v.2.0.0)*, *interactions(v.1.2.0)*, *ggpubr(v.0.6.1)*, *ggplot2(v.3.5.2)*, *effectsize(v.1.0.1)*, *rstatix(v.0.7.2)*, *bestNormalize(v.1.9.1)*, *berryFunctions(v.1.22.13)*, *emmeans(v.1.11.2)*, *scales(v.1.4.0)*, *psych(v.2.5.6)*, *kableExtra(v.1.4.0)*, *performance(v.0.15.0)*, *PerformanceAnalytics(v.2.0.8)*, *quantmod(v.0.4.28)*, *TTR(v.0.24.4)*, *xts(v.0.14.1)*, *zoo(v.1.8-14)*, *tidyquant(v.1.0.11)*, *car(v.3.1-3)*, *carData(v.3.0-5)*, *ltm(v.1.2-0)*, *polycor(v.0.8-1)*, *msm(v.1.8.2)*, *MASS(v.7.3-65)*, *lmerTest(v.3.1-3)*, *ordinal(v.2023.12-4.1)*, *lme4(v.1.1-37)*, *Matrix(v.1.7-3)*, *readxl(v.1.4.5)* and *knitr(v.1.50)*

loaded via a namespace (and not attached): *RColorBrewer(v.1.1-3)*, *rstudioapi(v.0.17.1)*, *datawizard(v.1.2.0)*, *magrittr(v.2.0.3)*, *TH.data(v.1.1-3)*, *estimability(v.1.5.1)*, *farver(v.2.1.2)*, *nloptr(v.2.2.1)*, *rmarkdown(v.2.29)*, *vctrs(v.0.6.5)*, *minqa(v.1.2.8)*, *base64enc(v.0.1-3)*, *butcher(v.0.3.5)*, *htmltools(v.0.5.8.1)*, *curl(v.6.4.0)*, *broom(v.1.0.9)*, *cellranger(v.1.1.0)*, *Formula(v.1.2-5)*, *parallelly(v.1.45.1)*, *htmlwidgets(v.1.6.4)*, *sandwich(v.3.1-1)*, *admisc(v.0.38)*, *lifecycle(v.1.0.4)*, *iterators(v.1.0.14)*, *pkgconfig(v.2.0.3)*, *R6(v.2.6.1)*, *fastmap(v.1.2.0)*, *rbibutils(v.2.3)*, *future(v.1.67.0)*, *digest(v.0.6.37)*, *numDeriv(v.2016.8-1.1)*, *colorspace(v.2.1-1)*, *furrr(v.0.3.1)*, *textshaping(v.1.0.1)*, *labeling(v.0.4.3)*, *timechange(v.0.3.0)*, *abind(v.1.4-8)*, *compiler(v.4.5.1)*, *rngtools(v.1.5.2)*, *withr(v.3.0.2)*, *doParallel(v.1.0.17)*, *htmlTable(v.2.4.3)*, *backports(v.1.5.0)*, *broom.mixed(v.0.2.9.6)*, *ggsignif(v.0.6.4)*, *lava(v.1.8.1)*, *ucminf(v.1.2.2)*, *tools(v.4.5.1)*, *foreign(v.0.8-90)*, *RobStatTM(v.1.0.11)*, *future.apply(v.1.20.0)*, *nnet(v.7.3-20)*, *glue(v.1.8.0)*, *quadprog(v.1.5-8)*, *nlme(v.3.1-168)*, *grid(v.4.5.1)*, *checkmate(v.2.3.3)*, *cluster(v.2.1.8.1)*, *see(v.0.11.0)*, *generics(v.0.1.4)*, *recipes(v.1.3.1)*, *gtable(v.0.3.6)*, *nortest(v.1.0-4)*, *tzdb(v.0.5.0)*, *class(v.7.3-23)*, *hms(v.1.1.3)*, *data.table(v.1.17.8)*, *xml2(v.1.4.0)*, *foreach(v.1.5.2)*, *pillar(v.1.11.0)*, *splines(v.4.5.1)*, *lattice(v.0.22-7)*, *survival(v.3.8-3)*, *tidyselect(v.1.2.1)*, *gridExtra(v.2.3)*, *reformulas(v.0.4.1)*, *bookdown(v.0.44)*, *svglite(v.2.2.1)*, *xfun(v.0.53)*, *expm(v.1.0-0)*, *hardhat(v.1.4.1)*, *timeDate(v.4041.110)*, *stringi(v.1.8.7)*, *yaml(v.2.3.10)*, *boot(v.1.3-31)*, *evaluate(v.1.0.4)*, *codetools(v.0.2-20)*, *cli(v.3.6.5)*, *rpart(v.4.1.24)*, *xtable(v.1.8-4)*, *parameters(v.0.27.0)*, *systemfonts(v.1.2.3)*, *Rdpack(v.2.6.4)*, *dichromat(v.2.0-0.1)*, *Rcpp(v.1.1.0)*, *globals(v.0.18.0)*, *coda(v.0.19-4.1)*, *parallel(v.4.5.1)*, *gower(v.1.0.2)*, *bayestestR(v.0.16.1)*, *doRNG(v.1.8.6.2)*, *listenv(v.0.9.1)*, *viridisLite(v.0.4.2)*, *mvtnorm(v.1.3-3)*, *ipred(v.0.9-15)*, *prodlim(v.2025.04.28)*, *insight(v.1.3.1)*, *rlang(v.1.1.6)*, *cowplot(v.1.2.0)*, *multcomp(v.1.4-28)*, *mnormt(v.2.1.1)* and *jtools(v.2.3.0)*

---

7 Supplementary references

Albers, C., & Lakens, D. (2018). When power analyses based on pilot data are biased: Inaccurate effect size estimators and follow-up bias. *Journal of Experimental Social Psychology*, *74*, 187–195. https://doi.org/10.1016/j.jesp.2017.09.004

Bates, D., Mächler, M., Bolker, B., & Walker, S. (2015). Fitting linear mixed-effects models using lme4. *Journal of Statistical Software*, *67*(1), 1–48. https://doi.org/10.18637/jss.v067.i01

Christensen, R. H. B. (2023). *Ordinal—regression models for ordinal data* [R package version 2023.12-4.1]. https://CRAN.R-project.org/package=ordinal

Gabry, J., Simpson, D., Vehtari, A., Betancourt, M., & Gelman, A. (2019). Visualization in Bayesian Workflow. *Journal of the Royal Statistical Society Series A: Statistics in Society*, *182*(2), 389–402. https://doi.org/10.1111/rssa.12378

Kuznetsova, A., Brockhoff, P. B., & Christensen, R. H. B. (2017). lmerTest package: Tests in linear mixed effects models. *Journal of Statistical Software*, *82*(13), 1–26. https://doi.org/10.18637/jss.v082.i13

Lenth, R. V. (2023). *Emmeans: Estimated marginal means, aka least-squares means* [R package version 1.8.9]. https://CRAN.R-project.org/package=emmeans

Long, J. A. (2019). *Interactions: Comprehensive, user-friendly toolkit for probing interactions* [R package version 1.1.0]. https://cran.r-project.org/package=interactions

Lüdecke, D., Ben-Shachar, M. S., Patil, I., Waggoner, P., & Makowski, D. (2021). performance: An R package for assessment, comparison and testing of statistical models. *Journal of Open Source Software*, *6*(60), 3139. https://doi.org/10.21105/joss.03139

Peterson, R., A. (2021). Finding Optimal Normalizing Transformations via bestNormalize. *The R Journal*, *13*(1), 310. https://doi.org/10.32614/RJ-2021-041

Peterson, R. A., & Cavanaugh, J. E. (2020). Ordered quantile normalization: A semiparametric transformation built for the cross-validation era. *Journal of Applied Statistics*, *47*(13–15), 2312–2327. https://doi.org/10.1080/02664763.2019.1630372

Rizopoulos, D. (2006). ltm: An R package for latent variable modeling and item response theory analyses. *Journal of Statistical Software*, *17*(5), 1–25. https://doi.org/10.18637/jss.v017.i05

Wickham, H. (2016). *Ggplot2: Elegant graphics for data analysis*. Springer-Verlag New York. https://ggplot2.tidyverse.org

Wickham, H., Averick, M., Bryan, J., Chang, W., McGowan, L. D., François, R., Grolemund, G., Hayes, A., Henry, L., Hester, J., Kuhn, M., Pedersen, T. L., Miller, E., Bache, S. M., Müller, K., Ooms, J., Robinson, D., Seidel, D. P., Spinu, V., … Yutani, H. (2019). Welcome to the tidyverse. *Journal of Open Source Software*, *4*(43), 1686. https://doi.org/10.21105/joss.01686

Wickham, H., François, R., Henry, L., Müller, K., & Vaughan, D. (2023). *Dplyr: A grammar of data manipulation* [R package version 1.1.3]. https://CRAN.R-project.org/package=dplyr

Xie, Y. (2014). Knitr: A comprehensive tool for reproducible research in R [ISBN 978-1466561595]. In V. Stodden, F. Leisch & R. D. Peng (Eds.), *Implementing reproducible computational research*. Chapman and Hall/CRC. https://doi.org/10.1201/9781315373461-1

Zhu, H. (2021). *Kableextra: Construct complex table with 'kable' and pipe syntax* [R package version 1.3.4]. https://CRAN.R-project.org/package=kableExtra
